# Acetaminophen-traces bioremediation with novel phenotypically and genotypically characterized 2 Streptomyces strains using chemo-informatics, in vivo, and in vitro experiments for cytotoxicity and biological activity

**DOI:** 10.1186/s43141-023-00602-w

**Published:** 2023-12-19

**Authors:** Donia H. Embarez, Ahmed S. Abdel Razek, Emad B. Basalious, Magdi Mahmoud, Nadia M. Hamdy

**Affiliations:** 1 Biochemistry Department, Faculty of Science, Ain Shams University, Cairo, 11566 Abassia Egypt; 2 Microbial Chemistry Department, Genetic Engineering and Biotechnology Research Division, National Research Centre, Giza, 12622 Dokki Egypt; 3 Department of Pharmaceutics and Industrial Pharmacy, Faculty of Pharmacy, Cairo University, Cairo, 11562 Al Kasr El-Aini Egypt; 4 Department of Biochemistry, Faculty of Pharmacy, Ain Shams University, Cairo, 11566 Abassia Egypt

**Keywords:** Bioremediation, Bio-degradation, Trace pharmaceuticals, Acetaminophen, Paracetamol, In silico, Cheminformatics, ADMET/PreADMET, *Streptomyces*, 16S rRNA

## Abstract

We isolated two novel bacterial strains, active against the environmental pollutant acetaminophen/Paracetamol®. *Streptomyces chrestomyceticus* (symbol RS2) and *Flavofuscus* (symbol M33) collected from El-Natrun Valley, Egypt—water, sediment, and sand samples, taxonomically characterized using a transmission electron microscope (TEM). Genotypic identification, based on 16S rRNA gene sequence analysis followed by BLAST alignment, were deposited on the NCBI as 2 novel strains https://www.ncbi.nlm.nih.gov/nuccore/OM665324 and https://www.ncbi.nlm.nih.gov/nuccore/OM665325. The phylogenetic tree was constructed. Acetaminophen secondary or intermediate product’s chemical structure was identified by GC/LC MS. Some selected acetaminophen secondary-product extracts and derived compounds were examined against a panel of test micro-organisms and fortunately showed a good anti-microbial effect. In silico chemo-informatics Swiss ADMET evaluation was used in the selected bio-degradation extracts for absorption (gastric), distribution (to CNS), metabolism (hepatic), excretion (renal), and finally not toxic, being non-mutagenic/teratogenic or genotoxic, virtually. Moreover, in vitro cytotoxic activity of these selected bio-degradation secondary products was examined against HepG2 and MCF7 cancer cell lines, where M33 and RS2 extract effects on acetaminophen/paracetamol bio-degradation products were safe, with higher IC50 on HepG2 and MCF7 than the acetaminophen/paracetamol IC50 of 108.5 μg/ml. Moreover, an in vivo oral acute single-dose toxicity experiment was conducted, to confirm these in vitro and in silico lower toxicity (better safety) than acetaminophen/paracetamol*.*

**Supplementary Information:**

The online version contains supplementary material available at 10.1186/s43141-023-00602-w.

## Introduction

### Problem definition

“Emerging pollutants” or “micro-constituents” also known as “trace pharmaceuticals” or “pharmaceuticals-traces” are substances that enter the water supply from human runoff [1]. When people dispose of their extra-unused or expired medications in the sink or toilet, or after frequent self-medication, pharmaceutical waste appears in a significant amount in wastewater, which, unfortunately, would make its way into the water system as well as the soil [2]. Crops irrigated with water containing trace pharmaceuticals, present in one of the most nowadays serious environmental problems, that would impact general health [3].

### Problem statement

The first problem is knowing that pharmaceutical waste and its metabolites are not intended for removal from domestic wastewater by conventional effluent treatments [1, 4]. Second, over time, we are subject to continuous exposure to low trace pharmaceutical concentrations, from drinking water or in crops irrigated with water containing these traces. Third, trace pharmaceuticals accumulate in our tissues causing cellular proliferation inhibition and/or chronic tissue damage from long-term exposure [5], a serious problem we have to face and solve to achieve “better health” as one of the Sustainable Development Goals (SDGs#3). Therefore, some of our “pharmaceutical biochemistry” research should be directed to get rid of these “trace pharmaceuticals” whatever their nature, whether antibiotics or analgesics.

## Background

Acetaminophen (APAP)/paracetamol® is one of the popular on-the-counter (OTC) analgesic, antipyretic, non-steroidal anti-inflammatory drug (NSAID) [1]. APAP is one of the “trace pharmaceuticals” that is present in different environmental domains like sediment, ground, and drinking water [2]. Although considered safe at therapeutic levels (4 g/day or less) [6], unmetabolized acetaminophen/paracetamol proved to inhibit ribonucleotide reductase enzyme leading to chromosomal aberration [7]. This would highlight the mandates for an effective creative method(s) degrading acetaminophen/paracetamol remaining at the domestic drainage [8]. Currently, sophisticated oxidation techniques and membrane technologies are the examined methodologies for the elimination of primary pharmaceutical contaminants, including acetaminophen/paracetamol [9]. Despite these methods being valid for primary waste, it is still not effective for secondary pollutants and trace acetaminophen/paracetamol bio-degradation products as well as their high cost constrain making these technologies unfavorable choices for wide application [10].

Hence, the solution should be green environment-friendly, cost-effective, easy, reliable, sustainable, and readily available. “Bioremediation” [11] presents a readily available option, potentially, fulfilling these mentioned requirements. The biological process of “bioremediation” uses micro-organisms (bacteria or fungi or both) that are already inhabitants in the near soil or water environment, where the micro-organism’s whole cell or their isolated enzymes act upon trace pharmaceuticals converting them into less harmful (harmless) forms. Examples of bacteria that can bio-degrade acetaminophen/paracetamol to produce fewer toxic products or less toxic products are *Corynebacterium nuruki*, *Bacillus drentensis*, *Pseudomonas moorei*, and *Acinetobacter bouvetii* [12]. Such an attempt presents a step-toward recommending “bacterial bioremediation” use at the domestic drainage to guard against adverse impacts from long-term exposure to trace pharmaceuticals.

### Research aim

To isolate and characterize the potential acetaminophen/paracetamol bio-degrading bacterial strain(s) from different Egyptian habitats. Objectives to fulfill the current study aim are first, screening several Egyptian habitats to characterize the successful isolated bacterial strain(s) able to bio-degrade acetaminophen/paracetamol, phenotypically and genotypically. The second objective is the characterization of the acetaminophen/paracetamol bio-degradation secondary/intermediate products either chemically or their biological down-stream targets, and ADME/toxicity predicted in silico and cheminformatics. Finally, the safety of these bio-degradation products will be biologically tested for their potential anti-microbial activity (if any) and tested for safety/toxicity experimentally both in vitro and in vivo.

## Materials

### Drugs, biochemical reagents, chemicals and solvents

Standard acetaminophen/paracetamol® gifted from Faculty of Pharmacy, Cairo University, Pharmaceutics Dept., Trypan blue dye, other chemicals, and reagents were purchased from Sigma-Aldrich Chemical Co. (St. Louis, MO, USA) and were either HPLC or molecular biology grade, per specifications. Dimethyl sulfoxide (DMSO) was used as the vehicle for acetaminophen/paracetamol derivatives per their molecular weight. Trypsin from AMRESCo, USA, and xylene, paraffin, and alcohol were of the highest grade commercially available.

### Media for cell culture

Handling procedure for the culture flask and subculturing RPMI media (Biowhittaker, Belgium) with fetal bovine serum (FBS) (GIBCo, USA), penicillin, and glutamine were all performed according to guidelines and procedures.

### Microbial culture suspension

The mineral salt media (MSM) [13] as cultivation media is used for bacterial growth. MSM is 1.5 agar plate containing per liter of deionized water the following mineral salts, purchased from Techno Pharma (India); 0.5 g of KH_2_PO_4_, 0.5 g K_2_HPO_4_, 0.01 g NaCl, 0.2 g MgCl_2_·6H_2_O, 0.02 g CaCl_2_, 0.339 mg MnSO_4_, 0.428 mg ZnSO_4_, 0.347 mg (NH_4_)_6_Mo_7_O_24_·4H_2_O, 0.4 mg of CoCl_2_·6H_2_O, and 10 mg EDTA. Lysogeny broth (LB) broth/LB agar (nutritionally rich medium for bacterial/fungal growth) from USBiological Life Sciences (USBio, USA) and bacterial culture suspension (co-culture) is supplied by AGP Pharma (Karachi, Pakistan).

## Methodology

### Biodiverse sample collection and processing

A diverse range of 13 samples was collected from different locations in Egypt to increase sample biodiversity and to explore different potential novel environmental microbiota, from October to November 2019. During a period of 14 days, in the morning, samples were collected from different places, with recording samples collecting the nature, map location, and the date of collection, using a sterile spatula; the sediment or sand, and water samples were taken from a depth of 5 to 25 cm and then transferred into sterile plastic bags numbered/coded.

### Fermentation of isolated bacterial strains

Collected samples were air-dried at room temperature for a week, preserved in falcon tubes, and kept in the fridge at 4°C.

To isolate inhabitant bacteria [14], sediment or desert sand samples were suspended in 50 ml sterile water and left for about 30 min at room temperature shaken from time to time. Decantation of the soil and filtration with a 0.45-mm Nalgene filter (Nalgene USA) was done.

Sediment and soil filtrate as well as water samples were diluted 10 folds with sterile distilled water, plated on 1.5% agar MSM media with 100µl of each dilution uniformly distributed on the plates. Plates were incubated at 30°C (Benchtop Laboratory Incubator BJPX-H; Biobase, China) for 48 h and then left at room temp. for 2 weeks to allow different bacterial colonies with different morphological characters to appear on plates (N.B. no colonies appeared in the pilot study done at higher temp.). On a daily basis, plates were inspected for any unwanted gram‐negative bacteria. Full colony appearance on plates occurred on day 7.

### Cultivation and screening of the isolated bacterial strains

Isolated single bacterial colonies from the previous exp. are now re-cultured in LB agar plates and tested for their ability to bio-degrade standard acetaminophen/paracetamol (500 mg/L) added to the media [15] as the sole carbon and nitrogen source.

Control media containing st. bacteria with/out acetaminophen/paracetamol added st. as well as media with added isolated samples with/out acetaminophen/paracetamol st. were run in duplicates parallel to the current work.

Part of the isolated re-cultured potential acetaminophen/paracetamol degrading bacteria was transferred to slant agar and stored at 4℃ for further processing. Another part of these bacterial cultures was stored as frozen stocks in 15% glycerol at − 80℃.

Each bacterial isolate was incubated at 30°C for 7 days in either static (ST) or shaking (SH) conditions, cultures are filtered using 0.45mM Nalgene filters (Nalgene, USA), and the filtrate was extracted repeatedly with an equal volume of ethyl acetate. Organic phases were collected and evaporated giving the minimal volume of “crude extracts” to analyze acetaminophen/paracetamol bio-degraded secondary/intermediate products within.

### Gas chromatography-mass spectrometry (GC–MS)

#### GC/electron ionization (EI)-MS analysis

Performed using a Thermo Scientific, Trace GC Ultra/ISQ Single Quadrupole MS, TG-5MS fused silica capillary column (30 m, 0.251 mm, 0.1-mm film thickness), and ionization energy of 70 eV, helium gas was used as the carrier gas at a constant flow rate of 1mL/min. The injector and MS transfer line temperature was set at 280°C.

Quantification of all the identified intermediate or secondary components investigated in the samples was done in comparison with structures deposited on the National Institute of Standards and Technology (NIST) Mass Spectrum Interpreter ver. 3.4, released Feb., 2019, which connects mass spectral peaks to their probable chemical structure origin (EI and MS/MS, both nominal and accurate mass) (https://www.nist.gov/mml/biomolecular-measurement/mass-spectrometry-data-center and https://chemdata.nist.gov/dokuwiki/doku.php?id=chemdata:start&s[]=gc&s[]=ei&s[]=ms).

### Structural determination (using liquid chromatography–mass spectrometry (LC–MS))

*Electrospray ionization (ESI) MS* [15–17] was used to operate the multiple-reaction monitoring mode of the Waters Xevo TQD LC–MS/MS instrument by Waters Corporation, Milford, MA01757, USA. For ES detection and EI detection, mass spectra were captured using the Schimadzu TripleQuadrupole GC–MS mass Spectrometer and the System mass spectrometer Accuity UPLC BEH C18 (1.7 µm, 2.1 × 50 mm) column, flow rate 0.2 mL/min. at Faculty of Pharmacy, Ain Shams University. Samples were prepared using ethyl acetate extraction and chromatographed by pumping solvent system of gradient (A) water and 0.1% formic acid, (B) methanol, (C) 50%H_2_O + 50%MeOH, and (D) acetonitrile and 0.1% formic acid (50:50, v/v) in an isocratic mode at a flow rate of 0.3 ml/min. Source voltages are 0.14 kV capillary and − 1 V cone. Dissolution temp. is 64°C, source gas flow is 3 L/h for desolvation, cone is 1 L/h, and analyzer collision energy is 2 V.

### Degradative pathways proposed in silico

This is done via Cheminformatics and Bioinformatics tools using the pharmacogenomics knowledge resource PharmGKB® [18] by NIH (https://www.pharmgkb.org/, accessed on November 3rd, 2022) and identified the small organic molecule acetaminophen/paracetamol-waste (APAP; *N*-acetyl-para-aminophenol) chemical structure and how it affects the biological systems.

PubChem [19] (https://pubchem.ncbi.nlm.nih.gov/) is used for acetaminophen/paracetamol bio-degradation product name identification, IUPAC name, molecular formula, weight, chemical structure, and Simplified Molecular-Input Line-Entry System (SMILES) as well as physical properties and biological activities.

EAWAG Bio-catalysis/Bio-degradation Database (BBD) is provided by the NCBI PubChem (USA) for information on microbial enzyme-catalyzed biocatalytic reactions and bio-degradation pathways for acetaminophen/paracetamol or by-products (https://pubchem.ncbi.nlm.nih.gov/source/EAWAG%20Biocatalysis/Biodegradation%20Database, http://eawag-bbd.ethz.ch/index.html and http://eawag-bbd.ethz.ch/predict/ accessed on February 15th, 2023).

### Physicochemical properties and ADMET in silico prediction

SwissADME is from Swiss Institute of Bioinformatics (SIB) (Lausanne/Switzerland) [20] (accessed on January 26th, 2022) using the individual degradation product SMILES notations from the PubChem (https://pubchem.ncbi.nlm.nih.gov/) submitted to the online server (http://www.swissadme.ch/) for calculation and knowledge about structure features: molecular weight (MW g/mol), the logarithm of the partition coefficient (log p), number of hydrogen bond acceptors (HBA), number of hydrogen bond donors (HBD), and the topological polar surface area (TPSA Å^2^), pharmacokinetic properties, and bioavailability of these biodegradation products.

PreADMET is used for drug-likeness prediction via Lipinski’s rule (https://preadmet.webservice.bmdrc.org/druglikeness/) to screen drug candidates of Lipinski’s rule [21] (https://preadmet.webservice.bmdrc.org/druglikeness-2/) “Rule of Five” in comparison to compounds from World Drug Index (WDI) database if the number of hydrogen bond donors is ≤ 5 (the sum of OHs and NHs); the number of hydrogen bond acceptor is ≤ 10 (the sum of Os and Ns); the compound molecular weight is ≤ 500 g/mol, with a lipophilicity consensus Log *P*_o/w_ CLogP ≤ 5 (MlogP ≤ 4.5); and then the compound is having better solubility and permeability (if around this figure will be moderately soluble).

SwissTargetPrediction [22] (http://www.swisstargetprediction.ch/index.php) is used to find out and identify the possible macromolecular downstream target(s) of the acetaminophen/paracetamol bio-degradation products that are assumed to be bioactive.

### Bacterial strains characterization

#### Phenotypic characterization

Samples are imaged using the whole-mount transmission electron microscopy (TEM) JEOL JEM-1010 (https://www.jeol.com/) at the Faculty of Pharmacy, Cairo University. The sample drop was placed on carbon-coated copper grids and left to dry at room temperature. Electron micrographs of chemically fixed and dried specimens were obtained using an accelerating voltage of 100 kV (= wavelengths of 0.0037nm).

#### Phylogenetic characterization

Total microbial genomic DNA was extracted from two promising bacterial strains A1, through E.Z.N.A.®Bacterial DNA Kit (D3350-01, Omega BIO-TEK, USA) according to manufacturer protocol.

PCR Amplification of 16S ribosomal RNA (rRNA) gene of the 2 bacterial strains was done by Dream Taq Green PCR Master Mix (2X) (K1081, Thermo Fisher, USA) according to manufacturer protocol through Creacon (Holland, Inc.).

PCR system cycler uses oligonucleotide universal actinomycete fungal strain primers [23] 27F 5′-AGA GTT TGA TCM TGG CTC AG-3′ and 1492R 5′-TAC GGY TAC CTT GTT ACG ACT T-3′ with target fragment length 1500 bp.

These were aligned together to generate a consensus sequence using DNA Baser Sequence Assembler v. 4.36 (https://www.dnabaser.com/ accessed in January 2022). The obtained aligned sequences were further identified by NIH/NLM Basic Local Alignment Search Tool (BLAST) (https://blast.ncbi.nlm.nih.gov/blast/Blast.cgi?CMD=Web&PAGE_TYPE=BlastHome).

Gel documentation system (Geldoc-it, UVP, England) is applied for data analysis using the image analysis software TotalLab V.1.0.1 (www.totallab.com). Genetic distances and multiple alignment of nucleic acid and protein sequences were computed by Pairwise Distance Method (PDM) using Clustal W2 v.2.1 [24]. Clustal W/X Clustal W/Clustal X Multiple alignment of nucleic acid and protein sequences (http://www.clustal.org/clustal2/) and nucleotide sequences were also compared with panel of st. bacterial isolates sequences available in the NIH genetic sequence database GenBank® [25] (https://www.ncbi.nlm.nih.gov/genbank/ accessed on February 2022).

Phylogenetic tree construction based on 16S rRNA gene region sequence is done using Molecular Evolutionary Genetics Analysis MEGA11 program (https://www.megasoftware.net/ downloaded for windows, accessed on February 2022).

In silico prediction of bacterial enzymatic activity by BacDive database [26] view IDs (by Leibniz-Institut DSMZ-Deutsche Sammlung von Mikroorganismen und Zellkulturen GmbH, Germany) (https://bacdive.dsmz.de/) via BRENDA [27] (https://www.brenda-enzymes.org/index.php) is a structured view of *Streptomyces* sp. enzymes (release 1, 2023 online including 68 new and 479 updated enzyme classes) by Elixir, USA, accessed on February 17th, 2023.

### Biological activity of the acetaminophen/paracetamol bio-degradation products

#### In vitro anti-microbial activity

According to the Broth Microdilution Method [28] in 96-well flat polystyrene plates, 10 µl of acetaminophen/paracetamol bio-degradation products samples was incubated with 80 µl of lysogeny broth media and 10 µl of microbial culture suspension standard solution of the tested microbial strains at 37°C (Benchtop laboratory incubator BJPX-H; Biobase, China) overnight (after 20 h). Bacterial growth was measured as absorbance at OD 600 nm using a Spectro Star Nano Microplate Reader (BMG LABTECH GmbH, Allmendgrun, Germany). Microbial counts were adjusted at 10^7^ colony-forming unit/ml (cfu/ml). The positive anti-microbial effect of the tested acetaminophen/paracetamol bio-degradation products samples monitored as.

$$\mathrm{Within}\;\mathrm{wells}\;\mathit`\mathit`\mathrm{Clearance}\;\mathrm{Zone}\!\mathit"\;\mathrm{in}\;\mathrm{mm}=\mathit\%\;\mathrm{inhibition}\;\mathrm{zone}$$

- According to www.ATCC.org, the current testing included *Escherichia coli* (*E. coli*) (ATCC 25922) and *Salmonella typhi* (*S. typhi*) (ATCC 6539) as Gram-negative bacterial strains. Gram-positive strains used are *Staphylococcus aureus* (*S. aureus*) (ATCC 25923) and *Bacillus subtilus* (*B. subtilus*) (ATCC 6633), multidrug-resistant *Staphylococcus aureus* (*MRSA*) (ATCC 43300), and fungi *Candida albicans (C. albicans)* (ATCC 10231) and *Aspergillus niger (A. niger)* (ATCC 6275).
- Non-selective light yellow solid nutrient agar plates containing peptone, yeast extracts, and sodium chloride, combined with beef extracts to provide the trace ingredients necessary for the growth of non-fastidious bacteria as well as nitrogen compounds, carbon, and vitamins.
- Test standard was suspension of every bacterium and fungus without any sample treatment and was all serially diluted.

In silico PreADMET ver 2.0 by Yonsei University, Yeonsu-gu, Incheon, Korea, by www.Cheminformatics.org accessed on January 26th, 2022, was used to predict pharmacokinetics and bioavailability of ADME parameters predicting small-molecule pharmacokinetic properties using graph-based signatures (pkCSM) [29] (https://biosig.lab.uq.edu.au/pkcsm/) for plasma protein binding, blood–brain barrier (BBB) penetration, skin penetration, and predict % human intestinal absorption (%HIA). Caco-2 cell model and MDCK cell model as reliable in vitro models for prediction of oral drug absorption.

In the in silico rodent oral toxicity prediction and indication of toxicity targets, PreADME/Tox used the PreADMET web server for Ames’ test toxicity prediction (https://preadmet.webservice.bmdrc.org/) as either “positive” (metabolic activation by rat liver 10% homogenate) or “negative” (https://preadmet.webservice.bmdrc.org/toxicity-prediction/).

## Experimental toxicity testing

In vitro 3-(4, 5-di-methylthiazol-2-yl)-2,5-diphenyl tetrazolium bromide MTT cytotoxicity assay to test cell viability or the half maximal inhibitory concentration (IC50) was done using two different cancer cell lines of human hepatocellular carcinoma (HepG2) [HEPG2] (ATCC HB-8065) cell line and human breast cancer cell line (MCF7) (ATCC HTB-22) according to the method described by Mosmann in1983 and Denizot and Lang in 1986 [30, 31].

HepG2 and MCF7 were seeded to 80% of cell confluency on 96 well plate (10 × 10^3^ cells/well) in RPMI medium with 10% fetal bovine serum (FBS), 1% penicillin, and 2 mM glutamine and then incubated at 37°C and 5% CO_2_ for 24 h, and then, cells were treated with either acetaminophen/paracetamol (positive control) or samples of the potential bacterial strain isolate bio-degradation products obtained in the shaking (SH) or the static (ST) states against DMSO non-treated cancer cells (negative control). Serial control or sample concentrations 2.44, 4.88, 9.77, 19.53, 39.06, 78.13, 156.25, 312.50, 625,1250, 2500, and 5000 µg/ml in RPMI serum-free media were used, then incubated for 48 h at 37°C and 5% CO_2_. After the incubation period, media were removed carefully, and 20 μl MTT stain was added in each well (5 mg/ml MTT solution in 1 × PBS) and incubated for 4 h at 37°C in CO_2_ incubator till the formation of MTT formazan crystals. MTT solution is now removed and crystals dissolved with 0.05 ml DMSO for 30 min on a shaker (Staurt, England). Absorbance measured spectrophotometrically at 570 nm with a reference wavelength of 630 nm using ELX800 UV universal microplate reader (BioTek Instruments Inc., VT, Santa Clara, California, USA).

$$\%\mathrm{cell}\;\mathrm{viability}=({\mathrm{mean}\;\mathrm{absorbance}}_{\mathrm{treated}\;\mathrm{sample}}/{\mathrm{mean}\;\;\mathrm{absorbance}}_{\mathrm{negative}\;\mathrm{control}\;\mathrm{sample}})\times100$$

$$\%\mathrm{death}\;\mathrm{rate}=100-(\%\mathrm{cell}\;\mathrm{viability})$$

IC50 was measured from the exponential curve of %cell viability against concentration (dose–response curve) [32] using Master–plex-2010 program.

### In vivo acute single oral toxicity study

Sample size estimation done in favor to ensure using the least sufficient animal number with ARRIVE no-harm to animals’ approach [33]. Considering the current study is superiority one-sided test as well as depending on a pilot/exploratory study done according to the resource approach [34] with an acceptable range of error degrees of freedom (D.F) (10–20 mice), and selecting one-tailed analysis repeated-measures ANOVA using design 3 for group comparison, the study power is set at 0.8, 5% risk type I error, so reducing the number of required animals by 14%.

### Experimental animals

Five- to 7-week-old Swiss Albino female mice weighing 15–20 g was obtained from Nile Co. for Pharmaceutical and Chemical Industries (Egypt). Mice were acclimatized for 1 week under standard laboratory conditions in cages in a room with a 12-h light/dark cycles, at 25 ± 2°C and 55 ± 5% relative humidity, at the Faculty of Pharmacy, Animal house, Ain Shams University, Abassia, Cairo, Egypt. Mice were fed on standard diet pellets purchased from El Nasr Company for Intermediate Chemicals, Giza, Egypt, containing no less than 20% protein, 3.5% fat, 6.5% ash, 5% fiber, and a vitamin mixture. Animals were allowed to a free access to drinking water ad libitum.

### Experimental procedure

Animals were divided randomly into five groups (5 animals per group) at the Animal House facility, Faculty of Pharmacy, Ain Shams University. All mice were made to fast for 24 h and treated once (day zero) with the acute single oral dose, using a gastric gavage. Animals were observed for the first 30 min and 4–5 times at intervals of 48 h to record any signs of abnormality, then carefully monitored for 14 days (end-point). The animals’ body weights (BW) were recorded before the experiment and at the end of the 14 days of observation.

### Experimental design

- Group 1: normal control group; mice received saline orally once.
- Group 2: negative control group; mice received vehicle (DMSO) orally once.
- Group 3: positive control group; mice given the reported published [35] acetaminophen/paracetamol acute oral dose (200 mg/kg BW) once.
- Group 4: received extracted sample 1b obtained from bacterial strain coded M33, while in the static condition, the dose used is the IC50 obtained from the in vitro cancer cell line results.
- Group 5: received extracted sample 1a obtained from bacterial strain coded M33, while in the in shaking condition, the dose used is the IC50 obtained from the in vitro cancer cell line results.

Biochemical analysis was done at the Advanced Biochemistry Research Lab (ABRL), Faculty of Pharmacy, Ain Shams University. Sera were prepared by centrifugation at 4000 rpm for 15 min from mice retro-orbital plexus blood samples collected and kept frozen at − 80°C for liver function tests (AST, ALT, and GGT colorimetrically).

Thereafter, mice were deprived of food overnight, euthanized, and sacrificed by cervical dislocation. Liver tissues were collected, washed with ice-cold saline, and weighed.

One liver tissue part was homogenized and kept frozen at − 80 °C for later analysis of mice IL-6 (iBL-America; ImmunoBiological Laboratories, Inc., Minneapolis, USA) and mice caspase-9 ELISA (Elabscience; CiteAb, USA) in liver tissue homogenate/mgm tissue protein (after total protein estimation in mice liver tissue homogenate colorimetrically by BCA).

Super oxide dismutase (SOD) and catalase (CAT) enzyme activities were estimated in liver tissue homogenate as an index of lipid peroxidation by colorimetric methods using Biodiagnostic kits, in accordance with previously established procedures. Levels of total antioxidant capacity (TAC) and the marker of lipid peroxidation malondialdehyde (MDA) were measured spectrophotometrically. MDA was measured as thiobarbituric acid (TBA) in an acidic medium at a temperature of 95°C for 30 min to form a colored thiobarbituric acid reactive (TBAR) product, and the absorbance of which was measured at 534 nm. The MDA kit was purchased from Biodiagnostics, Cairo, Egypt.

### Histopathological examination

Another liver part was excised, weighed, and fixed in 10% neutral-buffered formaldehyde overnight and then embedded in paraffin, deparaffinized in serial grades of alcohols, cleared in xylene, and was subjected to ultramicrotomy, where 4-μm-thick tissue sections were cut by rotatory microtome. Tissue sections were stained with hematoxylin and eosin (H and E) according *to* Culling [36], for histological examination using a full-HD light microscopic imaging system (Leica Microsystems GmbH, Wetzlar, Germany) [37].

### Statistical analysis

Data are presented as the mean ± SEM. All experiments were performed in triplicate and repeated twice. Multiple comparisons were done using a one-way analysis of variance (ANOVA) test followed by Duncan as a post hoc test. The statistical significance criterion used is the 0.05 level of probability *p*. Statistical analyses were carried out using IBM statistical package for social studies (SPSS) v.25 software (ISI software, San Diego, California, USA). Graphs done using GraphPad prism 8 San Diego, California, USA.

## Results

### Biodiverse collected samples

Thirteen samples of nature, type, and fermentation by isolated bacterial strains and their ability to bio-degrade st. acetaminophen are presented in Table 1, collected from different locations, either marine samples (water and sediment samples) or desert sand samples. Five sediment samples and five water samples were collected from Wadi El Natrun Valley lakes (Naba' El-Hamra, El-Hamra, EL Samaa, and El khadra) and from Faiyum City Qarun lake. Two sand samples were collected from Wadi El Natrun Valley and Al Giza Desert. One soil sample was collected from the First Industrial District, October City, Giza.

**Table 1:**
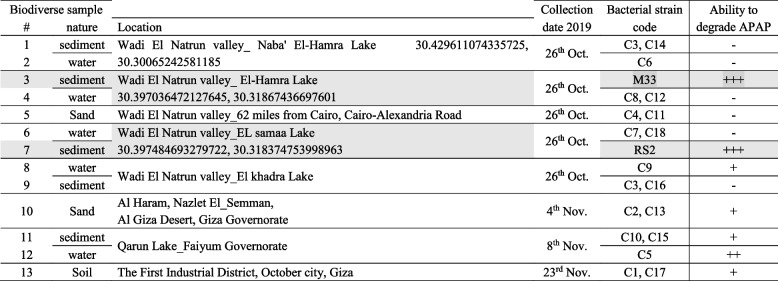
Egyptian biodiverse samples nature, map location, collection date, their ability to bio-degrade acetaminophen/paracetamol

Highlighted rows are the selected sediment samples coded as M33 and RS2 ( −), no ability to degrade acetaminophen (+ + +), high ability to degrade acetaminophen (+ or + +), moderate ability to degrade acetaminophen [APAP acetaminophen/paracetamol]

Two distinct bacterial strains were able to highly bio-degrade acetaminophen/paracetamol added to the media (coded as RS2 and M33) (Table 1). The ability to bio-degrade acetaminophen/paracetamol is measured as the zone of bacterial growth, on the expense of standard acetaminophen/paracetamol added in the bacterial culture media.

### Chemical identification of acetaminophen/paracetamol bio-degradation products

**Fig. 1:**
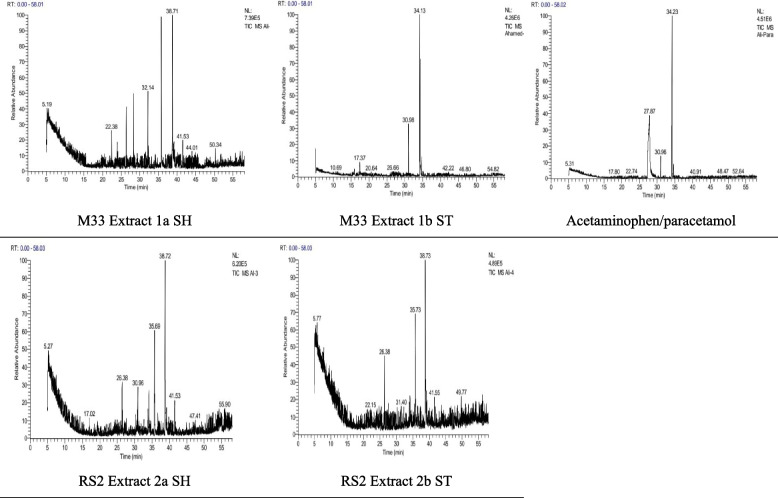
Quantification of 5 samples is done using a % relative peak area (RPA)

Table 2 presents the bio-degradation products obtained after cultivation and screening of the isolated bacterial strains. Quantification of 5 samples is done using a % relative peak area (RPA) in comparison of these compounds relative retention time (RRT) and mass spectrum interpreter software version 2 (https://chemdata.nist.gov/mass-spc/interpreter/) as in Fig. 1 presented in Table 1.

**Table 2:**
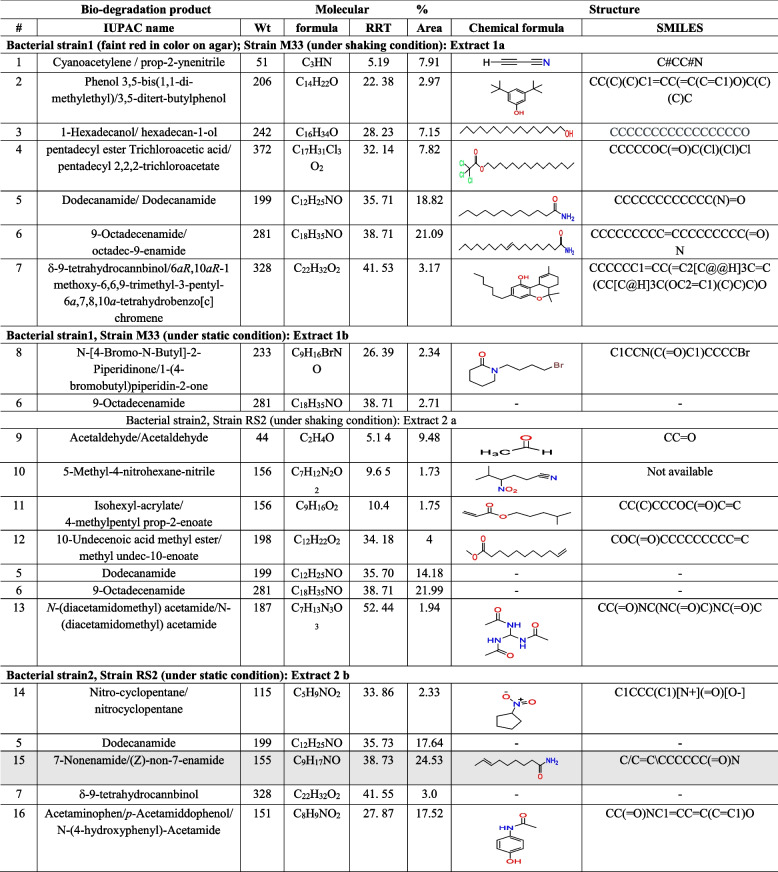
Acetaminophen/paracetamol bio-degradation products chemical identification in filtrates using GC/EI-MS system analysis

GC/EI-MS system analysis for structure via matching retention times and ion spectra with authentic standard and NIST library data search in silico. Mass spectrum interpreter software version 2 (https://chemdata.nist.gov/mass-spc/interpreter/) using the PharmGKB.® database (https://www.pharmgkb.org/chemical/PA448015) for acetaminophen/paracetamol and PubChem (https://pubchem.ncbi.nlm.nih.gov/) for the bio-degradation products (accessed on November 3rd, 2022)

*RRT* relative retention time, *RPA* % relative peak area

Acetaminophen/paracetamol (APAP; *N*-acetyl-para-aminophenol) and its bio-degradation products identity, chemical structure, molecular formula, and molecular weights in the filtrates were done using GC/MS system analysis. When SMILES were identified and used for each bio-degradation product and validation of the obtained chemical structure, molecular weight, via the in silico database PharmGKB® pharmacogenomics. Intermediate metabolites produced by acetaminophen/paracetamol bio-degradation were then examined in a hexane extract by GC–MS and IC. Peaks seen on the gas chromatogram denotes a distinct metabolite eluted closely together having relative retention times of 17.709 to 51.510. The batch culture did not contain any acetaminophen/paracetamol residue, according to the GC–MS data, then the *Streptomyces* strain can use acetaminophen/paracetamol as a source of energy via obtaining it from wastewater and hence, achieving its removal. Based on the sample’s peak and the RRT, acetaminophen/paracetamol catabolic pathway metabolites would raise the possibilities of acetaminophen/paracetamol bio-degradation pathways involving metabolites appearing in Table 2. Acetaminophen/paracetamol bio-degradation products in Table 2 are secondary degradation compounds as we obtained with annotation (https://pubchem.ncbi.nlm.nih.gov/compound/7689#section=Related-Records).

**Fig. 2:**
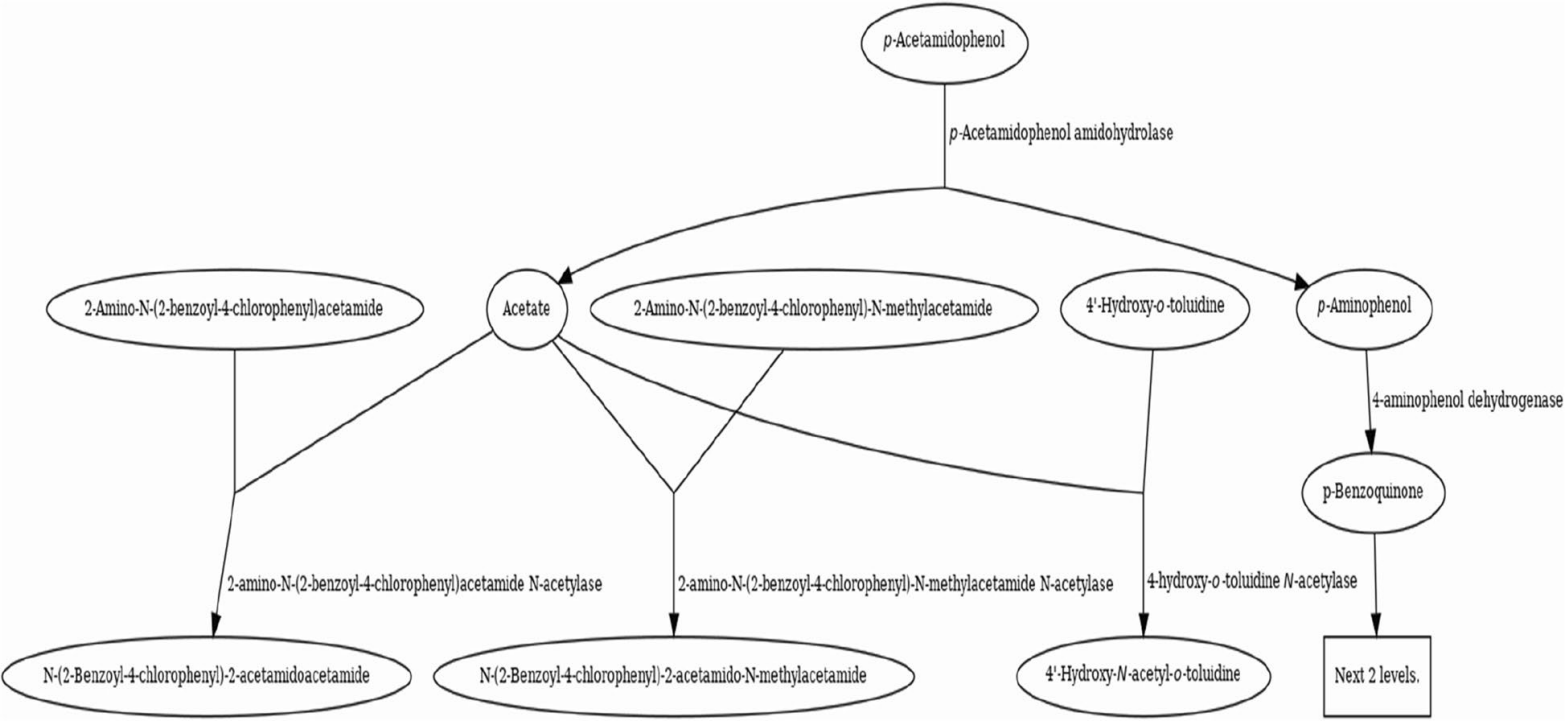
Acetaminophen/paracetamol bio-degradation pathway proposed diagram (first 2 reaction levels) retrieved from EAWAG-BBD pathway map starting with reaction r1629 (accessed on Feb. 16, 2023) (http://eawag-bbd.ethz.ch/servlets/pageservlet?ptype=r&reacID=r1629)

The EAWAG bio-catalysis/bio-degradation database (BBD) *(*http://eawag-bbd.ethz.ch/servlets/dpage?ptype=p&reacID=r1629&max_rows=0&smpth=false*)* is used *for* the *prediction of acetaminophen/paracetamol bio-degradation products pathway(s) and related enzymes involved in its map presented in *Fig. 2* (*p-Acetamidophenol ––- > p-Aminophenol + Acetate (reacID# r1629)).

Supplementary Figure S1 presents the acetaminophen/paracetamol bio-degradation proposed whole pathway/reaction diagram retrieved from the EAWAG-BBD pathway map starting with reaction r1629 (accessed on Feb. 16th, 2023).

It is noteworthy to mention that some of the bio-degradation product pathways/reactions in Table 2 of the EAWAG-PPS do not predict (http://eawag-bbd.ethz.ch/predict/notbepredicted.html#Reactions) that is because they are environmental-degradation reactions or too difficult reactions to predict as detoxification reactions involving conjugation or azo compounds formed from primary amide (-NH_2_) groups as compounds 2, 7, and 13 in Table 2. Moreover, the acetylation of primary amines as compounds 5, 6, and 13 or the formation of intramolecular rings as compounds 7, 8, and 14 is shown in Table 2. Finally, one of the options that make products not predicted and not present on any database is the hydroxylation of aliphatic carbon atoms through environmental non-specific monooxygenases. However, compounds containing acetylene or cyanide (compound #1), acetate (compound #4), deconoate (compound #5), and proponoate (compound #11) in Table 2 are termination compounds (http://eawag-bbd.ethz.ch/servlets/pageservlet?ptype=termcompsview) that are not predicted also.

Reactions begin with 4-acetamidophenol via p-acetamidophenol amidohydrolase enzyme (amidase) (EAWAG-BBD enzyme, enzymeID# e1067) to give acetate and 4-aminophenol. *N*-methyl acetamide *N*-acetylase enzyme (EAWAG-BBD reaction, reacID# r1762) is involved in the bio-degradation of acetate and 4-aminophenol.

### In silico prediction of physicochemical properties (Table 3) (accessed on November 2022)

**Table 3:**
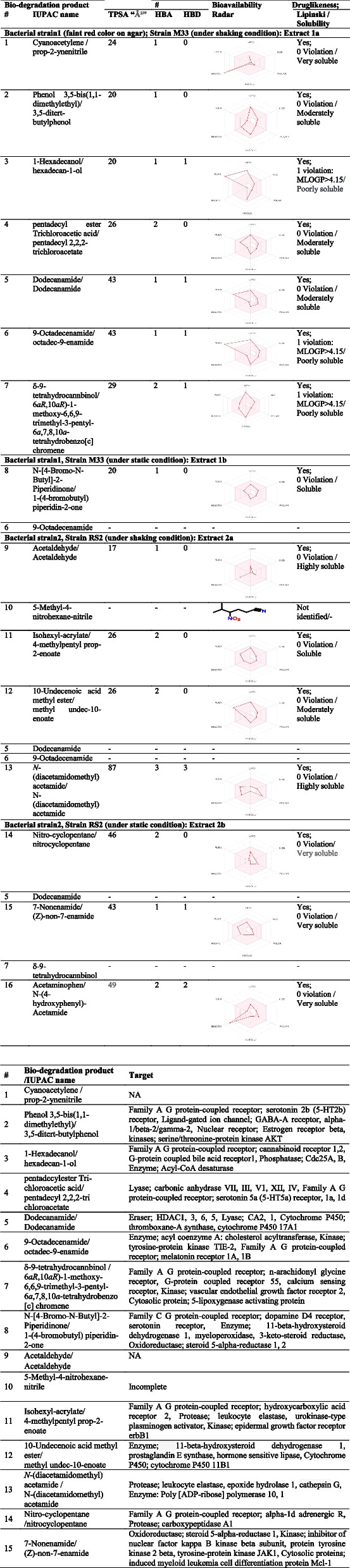
Acetaminophen/paracetamol bio-degradation products physicochemical properties, bioavailability radar charts, lipophilicity, Lipinski rule in silico prediction

Physicochemical properties are hydrogen bond numbers and TPSA, and the Lipinski rule is the solubility/permeability drug-likeness. In silico prediction was done by using either the SwissADME cheminformatics platform or the WDI database. Bioavailability RADAR charts for six important physicochemical properties determined from the SwissADME cheminformatics platform (http://www.swissadme.ch/) and Lipinski’s rule (https://preadmet.webservice.bmdrc.org/druglikeness-2/), “Rule of Five” in comparison to compounds from the WDI database for solubility and permeability determination (accessed in November 2022)

HBA hydrogen bond acceptors, HBD number of hydrogen bond donors, TPSA topological polar surface area, LIPO lipophilicity XLOGP3, SIZE size MW g/mol, POLAR polarity TPSA “Å^2^”, INSOUL solubility log S, INSATU saturation fraction of carbons, FLEX flexibility

Calculation and knowledge about structure features physicochemical properties and pharmacokinetic properties of these bio-degradation products (Table 3) are presented as bioavailability RADAR charts for six important physicochemical properties. The pink area represents the optimal range for each property: lipophilicity (LIPO) XLOGP3 between − 0.7 and + 5.0, size (SIZE) MW between 150 and 500 g/mol, polarity (POLAR) TPSA between 20 and 130 “Å^2^” solubility (INSOLU) log S not higher than 6, saturation (INSATU) fraction of carbons in the sp 3 hybridization not less than 0.25, and flexibility (FLEX) no more than nine rotatable bonds. Bioavailability Radar charts for physicochemical properties determined from the SwissADME cheminformatics platform.

*Drug-likeness prediction by PreADMET cheminformatics database (*Table 3*) (accessed on November 2022). This is according to Lipinski’s rule* (https://preadmet.webservice.bmdrc.org/druglikeness-2/), the “Rule of Five” in comparison to compounds from the WDI database for solubility and permeability determination.

Now, if we assume acetaminophen/paracetamol bio-degradation secondary or intermediate products as bioactive, downstream targets were predicted chemo-informatically using the PreADMET database that identified these target(s) enzymes, receptors, or cytosolic and membrane proteins (Table 4) taking into consideration that the bio-degradation product compounds with less than 5 heavy atoms cannot be submitted for prediction.

**Table 4:** Acetaminophen/paracetamol-bio-degradation products downstream enzymes, receptors, or cytosolic/membrane protein target(s) in silico prediction

#	Bio-degradation product/IUPAC name	Target
1	Cyanoacetylene/prop-2-ynenitrile	NA
2	Phenol 3,5-bis(1,1-dimethylethyl)/3,5-ditert-butylphenol	Family A G-protein-coupled receptor; serotonin 2b (5-HT2b) receptor, ligand-gated ion channel; GABA-A receptor, alpha-1/beta-2/gamma-2, nuclear receptor; estrogen receptor beta, kinases; serine/threonine-protein kinase AKT
3	1-Hexadecanol/hexadecan-1-ol	Family A G-protein-coupled receptor; cannabinoid receptor 1,2, G-protein-coupled bile acid receptor1, phosphatase; Cdc25A, B, enzyme; Acyl-CoA desaturase
4	pentadecylester Tri-chloroacetic acid/pentadecyl 2,2,2-tri chloroacetate	Lyase; carbonic anhydrase VII, III, V1, XII, IV, family A G-protein-coupled receptor; serotonin 5a (5-HT5a) receptor, 1a, 1d
5	Dodecanamide/Dodecanamide	Eraser; HDAC1, 3, 6, 5, lyase; CA2, 1, cytochrome P450; thromboxane-A synthase, cytochrome P450 17A1
6	9-Octadecenamide/octadec-9-enamide	enzyme; acyl-coenzyme A: cholesterol acyltransferase, kinase; tyrosine-protein kinase TIE-2, family A G-protein-coupled receptor; melatonin receptor 1A, 1B
7	δ-9-tetrahydrocannbinol/6*aR*,10*aR*)-1-methoxy-6,6,9-trimethyl-3-pentyl-6*a*,7,8,10*a-*tetrahydrobenzo [c] chromene	Family A G-protein-coupled receptor; n-arachidonyl glycine receptor, G-protein coupled receptor 55, calcium-sensing receptor, kinase; vascular endothelial growth factor receptor 2, cytosolic protein; 5-lipoxygenase activating protein
8	N-[4-Bromo-N-Butyl]-2-Piperidinone/1-(4-bromobutyl) piperidin-2-one	Family C G-protein-coupled receptor; dopamine D4 receptor, serotonin receptor, enzyme; 11-beta-hydroxysteroid dehydrogenase 1, myeloperoxidase, 3-keto-steroid reductase, oxidoreductase; steroid 5-alpha-reductase 1, 2
9	Acetaldehyde/Acetaldehyde	NA
10	5-Methyl-4-nitrohexane-nitrile	Incomplete
11	Isohexyl-acrylate/4-methylpentyl prop-2-enoate	Family A G-protein-coupled receptor; hydroxycarboxylic acid receptor 2, protease; leukocyte elastase, urokinase-type plasminogen activator, kinase; epidermal growth factor receptor erbB1
12	10-Undecenoic acid methyl ester/methyl undec-10-enoate	Enzyme; 11-beta-hydroxysteroid dehydrogenase 1, prostaglandin E synthase, hormone-sensitive lipase, cytochrome P450; cytochrome P450 11B1
13	*N*-(diacetamidomethyl) acetamide/N-(diacetamidomethyl) acetamide	Protease; leukocyte elastase, epoxide hydrolase 1, cathepsin G, enzyme: poly[ADP-ribose] polymerase 10, 1
14	Nitro-cyclopentane /nitrocyclopentane	Family A G-protein-coupled receptor; alpha-1d adrenergic R, protease; carboxypeptidase A1
15	7-Nonenamide/(Z)-non-7-enamide	Oxidoreductase; steroid 5-alpha-reductase 1, kinase; inhibitor of nuclear factor kappa B kinase beta subunit, protein tyrosine kinase 2 beta, tyrosine-protein kinase JAK1, cytosolic proteins; induced myeloid leukemia cell differentiation protein Mcl-1

Using PreADMET cheminformatic database: SwissTargetPrediction (http://www.swisstargetprediction.ch/index.php) (accessed on November 2022)

*NA* not applicable to obtain target as molecules with less than 5 heavy atoms cannot be submitted for prediction

### The most potent bacterial strain(s) characterization

#### Phenotypic characterization (Fig. 3)

**Fig. 3:**
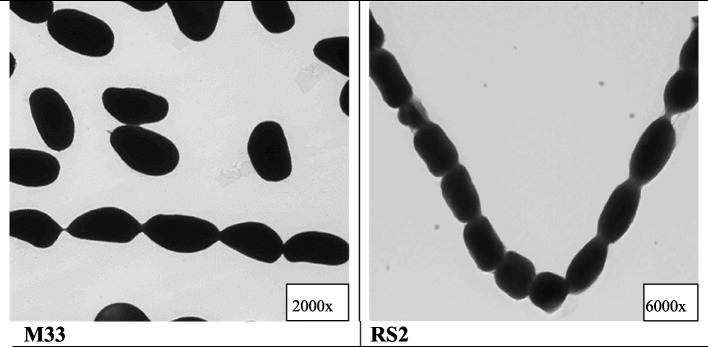
Phenotypic identification of the isolated bacterial strains; M33 and RS2, imaging done by the whole mount TEM, with magnification scale 2000 × and 6000 × , respectively. A spiral smooth spore surface ornamentation/chain type is confirmatory of the bacterial strain. Accelerating voltages = 100 kV. [× , direct magnification], smooth-surfaced long-chained spores of globose shape are confirmatory of *Streptomyces*

Using the TEM whole mount method smooth surfaced long-chain spores of globose shape are confirmatory of *Streptomyces* sp.

### Phylogenetic characterization (Fig. 4)

**Fig. 4:**
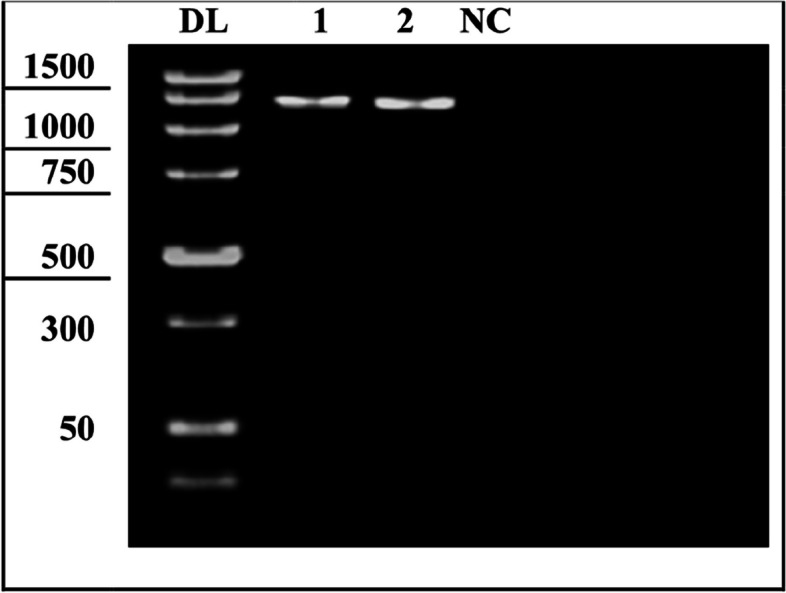
Molecular weight detection of specific amplified products of 16S ribosomal RNA gene region for two samples with approx. 1500 bp [Sample 1 is 1414 bp and sample 2 is 1443 bp]. DL DNA ladder from actinomycetes, NC negative control

Phylogenetic sequence analysis after molecular weight detection of the amplified products of 16S ribosomal RNA gene region for two samples with approx. 1500 bp, sample 1 was 1414 bp and sample 2 was 1443 bp, in comparison to a st. ladder actinomycetes of known Mwt. DNA and the negative control.

Identified 16S ribosomal RNA gene sequences were aligned and analyzed for identity and genetic distances using NCBI BLAST. Table 5 addresses the 16S rRNA gene, partial sequence, and highest similarity % among the 5 samples for M33 and RS2 bacterial strain: Actinomycetota, Streptomycetales, Streptomycetaceae, Streptomyces spore-producing, and antibiotic-producing organisms isolated from the soil. Phylogenetic analysis showed one main clade in which isolate M33 was amongst other Streptomyces strains in the same clade. A similar 16S rRNA gene sequence belonging to Streptomyces flavofuscus strain NRRL B-8036 is under the same category.

**Table 5:** 16S ribosomal RNA gene, partial sequence, and highest similarity % among M33 and RS2 samples *Streptomyces* strains

Characteristics	Bacterial strain isolates	
Our identification code	**RS2**	**M33**
GeneBank or version	OM665324.1	*OM665325.1*
Accession or locus	OM665324	*OM665325*
Current NCBI deposition link	https://www.ncbi.nlm.nih.gov/nuccore/OM665324	https://www.ncbi.nlm.nih.gov/nuccore/OM665325
Previous NCBI deposition links	https://www.ncbi.nlm.nih.gov/nuccore/206581545	https://www.ncbi.nlm.nih.gov/nuccore/DQ026648.1
Strain name	*Streptomyces chrestomyceticus* strain A1	*Streptomyces flavofuscus* strain A2
Source strain	13663Q	NRRL B-8036
Tested sample bases	1 to 1429	1 to 1397
Ref. strain DNA bp	1 to 1499	1 to 1513
Identity %	99.57	99.35
NCBI species taxonomy ID	68,185	332,582

Accession number or version from GeneBank. [Aligned sequences were analyzed on the NCBI website (http://www.ncbi.nlm.nih.gov/webcite) using BLAST to confirm their identity. Genetic distances and multiple alignment sequences were computed by the Pairwise Distance method using Clustal W2 v.2.1 (http://www.clustal.org/clustal2/). The nucleotide sequences were compared with a panel of st. bacterial isolates sequences available in the GenBank.] (accessed on January 2022). Submitted to the NCBI/nucleotide/gene bank on February 13th, 2022

**Fig. 5:**
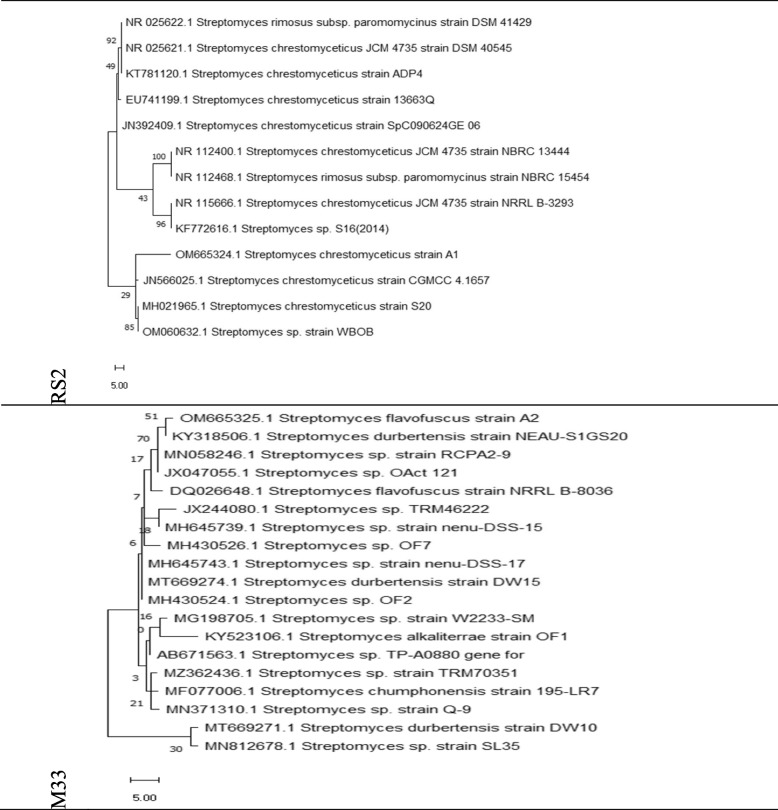
The 2 isolated bacterial stains (RS2 and M33) neighbor-joining (NJ) phylogenetic tree based on 16S rRNA gene region sequence. [NJ-phylogenetic tree drawing was done using MEGA11 program (https://www.megasoftware.net/). Phylogenetic analysis is done using the Clustal W2 program.]. The tree shows the relationship between *Streptomyces* sp. and a number of isolates and closely related type strains of the genus *Streptomyces.* Bootstrap values (calculated as % from 500 replications) are indicated. The tree has been drawn to scale with branch lengths measured in the number of substitutions per site, with a free ratio model

Phylogenetic tree obtained by neighbor-joining (NJ) and unweighted pair group method with arithmetic means (UPGMA) to obtain a tree with a free ratio model. Figure 5 presents the phylogenetic tree for the 2 *Streptomyces* strains based on the previous 16S rRNA gene region sequence from the isolated samples RS2 and M33 using the MEGA11 program (https://www.megasoftware.net/).

#### In silico prediction of the isolated bacterial strain enzymatic activity

*By BacDive database* was accessed on February 12th, 2023 (https://bacdive.dsmz.de/strain/15148). Enzyme activities included acid and alkaline phosphatases, alpha-chymotrypsin and glucosidase, and naphthol-AS-BI-phosphohydrolase.

*Via BRENDA* structured view of 68 new enzymes and 479 updated enzyme classes, accessed on February 17, 2023. *Streptomyces* sp. enzymes are *Streptomyces* aminopeptidase, *Streptomyces* dinuclear aminopeptidase, thermophilic *Streptomyces* serine proteinase, and proteinase (https://www.brenda-enzymes.org/search_result.php?quicksearch=1&noOfResults=10&a=9&W[2]=Streptomyces+&T[2]=2).

Streptomyces chrestomyceticus *enzymes are hydrolase related to hydrolysis of proteins, favoring hydrophobic residues, gelatinase, and urease *(https://www.brenda-enzymes.org/search_result.php?quicksearch=1&noOfResults=10&a=21&W[2]=Streptomyces+chrestomyceticus+&T[2]=1&V[8]=1).

### Isolated acetaminophen/paracetamol bio-degradation products biological activity

#### In vitro anti-microbial activity (Table 6)

**Table 6:** Anti-microbial activity monitored as growth inhibition % of isolated acetaminophen/paracetamol bio-degradation products in static or shaking states for M33 bacterial strain isolate

Microorganisms in culture suspension		M33-tested samples	
		**1a SH**	**1b ST**
Gram negative	*S. Arues*	-ve	71.48
	*E. Coli*	low	82.18
Gram positive	*MRSA*	81.89	low
	*B. Subtills*	-ve	-ve
	*S. Typhi*	-ve	-ve
Fungi	*C. Albicans*	-ve	-ve
	*A. Niger*	70.13	94.66

Values are the average of triplicate experiments

*E. coli*, *Escherichia coli*; *S. typhi*, *Salmonella typhi*; *S. aureus*, *Staphylococcus aureus*; *MRSA*, multidrug-resistant *Staphylococcus aureus*; *B. subtilus*, Bacillus subtilus; *C. albicans*, *Candida albicans*; *A. niger*, *Aspergillus niger*; *1a*, M33 degradation products in shaking flask. *1b*, M33 degradation products in a static flask. *-ve*, no inhibition zone appeared or less than 10% opaque media, if less than 50% inhibition = low activity, inhibition % = positive antimicrobial effect presented as clearance in the wells

The positive anti-microbial effect was observed as clearance in the wells/inhibition zone % vs compounds with no anti-microbial effect the growth media appeared opaque with viable bacteria surviving. The positive anti-microbial effect of the tested acetaminophen/paracetamol bio-degradation products (arising from M33) was observed in the shaken sample products, 1a against *MRSA* and *A. niger*. For the static sample products 1b ST an anti-microbial effect of 71.5%, 82%, and 94.5% growth inhibition against *S. arues*, *E. coli*, and *A. niger*, respectively.

#### ADMET in silico prediction (accessed on January 26th, 2022) (Table 7)

**Table 7:** ADMET parameters or PreADME/Tox prediction for acetaminophen/paracetamol bio-degradation products

Bio-degradation product	Absorption A	Distribution D	Metabolism M	Excretion E	Toxicity T	
	**GI % intestinal human abs**	**(log BB) BBB/CNS permeation**	**CYP2D6 inhibitor**	**Renal OCT2 substrate**	**AMES/skin sensitization**	**Hepatotoxicity/ hERG I inhibitor**
Cyanoacetylene /prop-2-ynenitrile	100/high	− 0.039/no	No	No	No/no	No/No
Phenol 3,5-bis(1,1-dimethylethyl)/ 2,4-ditert-butylphenol	100/high	− 0.039/no	No	No	No/no	No/No
1-Hexadecanol/hexadecan-1-ol	91.6/high	0.408/yes	Yes	No	No/yes	No/No
Pentadecyl ester trichloroacetic acid/pentadecyl 2,2,2-trichloroacetate	89.8/high	0.798/yes	No	No	No/yes	No/No
Dodecanamide/dodecanamide	93/high	0.362/yes	No	No	Yes/yes	No/No
9-Octadecenamide/octadec-9-enamide	92/high	− 0.172/yes	No	No	No/yes	No/No
δ-9-Tetrahydrocannbinol/6aR,10aR)-1-methoxy-6,6,9-trimethyl-3-pentyl-6a,7,8,10a-tetrahydrobenzo[c] chromene	90.2/high	− 0.389/yes	No	No	No/yes	No/No
*N*-[4-Bromo-*N*-Butyl]-2-Piperidinone/1-(4-bromobutyl)piperidin-2-one	93.1/high	0.448/yes	No	No	No/no	No/No
Acetaldehyde/Acetaldehyde	93.2/high	0.58/yes	No	No	Yes/yes	No/No
5-Methyl-4-nitrohexane-nitrile	100/low	− 0.023/no	No	No	No/no	No/No
Isohexyl-acrylate/4-methylpentyl prop-2-enoate	Not identified	NA	NA	NA	NA	NA
10-Undecenoic acid methyl ester/methyl undec-10-enoate	95.4/high	0.464/yes	No	No	No/yes	No/No
*N*-(diacetamidomethyl)acetamide	95.1/high	0.669/yes	No	Yes	No/yes	No/No
Nitrocyclopentane/nitrocyclopentane	100/high	− 0.278/yes	No	No	Yes/yes	No/No
7-Nonenamide/(Z)-non-7-enamide	93.3/high	− 0.011/yes	No	No	No/yes	No/No
Acetaminophen/*N*-(4-hydroxyphenyl)-acetamide	92/high	− 0.219/yes	No	No	No/no	No/No

Using the SwissADME cheminformatics platform and the pkCSM Web tool in silico prediction done using the pkCSM Web tool (https://biosig.lab.uq.edu.au/pkcsm/prediction and http://www.swissadme.ch/) accessed on January 26th, 2022

*BBB* blood–brain barrier, *CNS* central nervous system,*GI* gastrointestinal, *predict %* human intestinal absorption (%HIA), *hERG* human ether-à-go-go-related gene cardiac potassium channel, *NA* not identified

##### In silico ***rodent oral toxicity prediction and indication of toxicity targets***

The SwissADME cheminformatic platform as well as via pkCSM and PreADME/Tox database is used to examine interaction with other body proteins and to ensure that these bio-degradation products are safe, with no carcinogenic effect via in silico to be non-mutagenic according to Ames’ test; furthermore, carcinogenicity in animals in vivo (mice) is expected to be negative with a modest risk according to the human ether-à-go-go-related gene cardiac potassium channel (hERG) ion channel inhibition, which is an important anti-target in drug discovery, associated with potentially fatal heart conditions.

### Toxicity testing

In vitro MTT cytotoxicity assay (Table 8) using HepG2 and MCF7 cancer cell lines treated with acetaminophen/paracetamol or Extract 1a, Extract 1b, Extract 2a, and Extract 2b with different concentration ranges. Cancer cell viability decreased by increasing the concentration for all the bio-degradation product samples. IC50 µg/ml of extracts 1a, 1b, 2a, and 2b were higher than acetaminophen/paracetamol IC50 µg/ml (safer). The average M33 isolate samples (1a and 1b) IC50 were 200 µg/ml.

**Table 8:** IC50 µg/ml identification of acetaminophen/paracetamol bio-degradation products in vitro cytotoxicity MTT assay using HepG2 and MCF7 cancer cell lines

	**Isolates samples**				**Control**	
	**M33**		**RS2**		** + ve**	**-ve**
**Cells**	**1a SH**	**1b ST**	**2a SH**	**2b ST**	**APAP**	**DMSO**
HepG2	192.3	200.6	126.6	119.9	108.6	-
MCF7	441.7	370	285	305	108.4	-

The selected bacterial isolates M33 and RS2 strains, from samples 1a, 1b, 2a, and 2b in vitro cytotoxicity MTT assay against acetaminophen/paracetamol (positive control) using 2 different cancer cell lines (HepG2 and MCF7) as well as a control one

*SH*, shaking state; *ST*, static state; *APAP*, acetaminophen/paracetamol

DMSO is the negative control. % death rate = 100 – (% cell viability), % cell viability = (mean absorbance of treated sample/mean absorbance of negative control sample) × 100, and the inhibitory concentration of 50% (IC50 µg/ml) measured from the exponential curve of viability against concentration (dose–response curve) using Master –plex-2010 program. All experiment conditions were done in triplicates

See Supplementary Table S1 for detailed Table 8 results. HepG2 and MCF7 IHC photo-micrographs are imaged by an inverted microscope (Supplementary Figures S2) (https://drive.google.com/folderview?id=1XYW6tBq3g9Vw78Q7hQlIuK0ZOHDraPBm).

#### In vivo acute single oral toxicity test (Fig. 6)

**Fig. 6:**
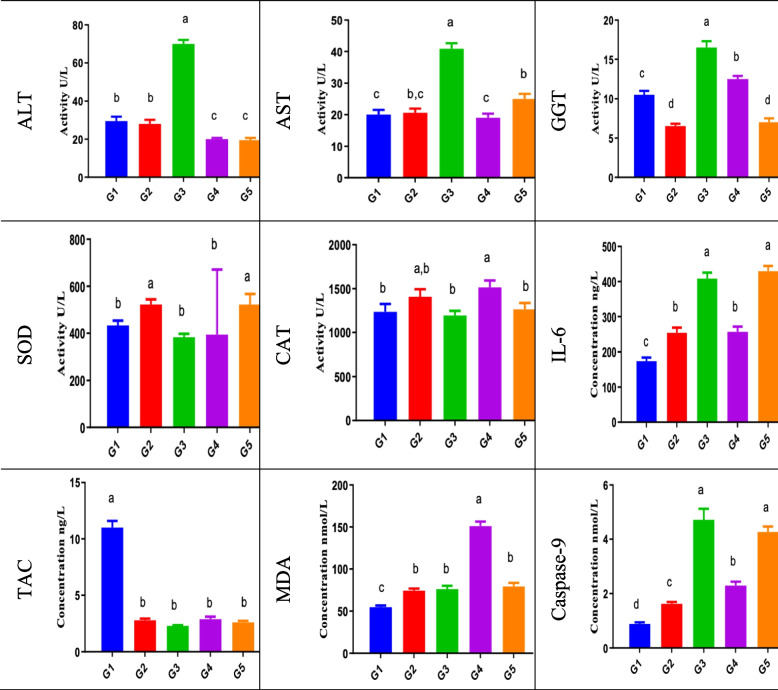
Effect of acute single oral dose in vivo toxicity testing (200 mg/k.g BW) of acetaminophen/paracetamol and the bio-degradation products (IC50 dose) on blood liver function tests, liver tissue oxidative stress markers, antioxidant levels, liver tissue IL-6, and caspase-9

##### N.B.1.

No estimation of acute oral toxicity of the acetaminophen/paracetamol bio-degradation products extracted from the bacterial strain RS2 Extract 2a and 2b in either static or shaking flasks, per the bio-degradation product, was highly similar in structure, and the anti-microbial effect and the ADMET and SwissTox predicted that they are not toxic. This is to obey the OECD Guideline No. 407 and the ARRIVE guidelines (https://arriveguidelines.org/) to use the least number of mice/group for the oral acute single-dose toxicity test.

##### N.B.2.

The objective of the acute oral toxicity study was not LD50 determination. Serum liver function tests (ALT, AST, GGT) showed a significant increase in the acetaminophen/paracetamol group (group 3) compared to the normal control group (group 1) and the negative control group (DMSO) (group 2). Bio-degradation product groups (groups 4 and 5) showed relatively normal serum liver function test values compared to the normal control group (group 1). Liver tissue lipid peroxidation level was expressed as MDA showing a significant increase in group 4 compared to the normal control group (group 1) and a relative increase in the acetaminophen/paracetamol group (group 3) and group 5 compared to the normal control group (group 1).

Liver tissue oxidative stress markers are SOD and MDA, and liver tissue antioxidant levels are TAC and CAT. Data are expressed as mean ± SEM in sera or per mgm tissue protein in the tissue homogenate/total protein conc. (ng/ml). Experiments were performed in triplicate and repeated twice. The significance criterion is set to 0.05 level of probability *p.* a > b > c > d significance obtained by groups mean comparison difference by ANOVA followed by Duncan post hoc test [group 1, normal control; group 2, DMSO negative control; group 3, acetaminophen/paracetamol positive control; group 4, M33 Extract 1b; group 5, M33 Extract 1a].

Liver tissue CAT antioxidant enzyme activity was depressed in the acetaminophen/paracetamol group (group 3). However, group 4 CAT activity was relatively similar to the normal control group activity, while group 4 showed relatively increased CAT activity compared to the normal control group. Liver tissue total antioxidant capacity (TAC) showed similar results to CAT. Liver tissue inflammatory mediator marker IL-6 and the apoptosis initiator enzyme caspase-9 showed a significant increase in acetaminophen/paracetamol group (group 3), group 5, and group 4 in comparison to the normal control group (group 1) or the negative control group (group 2). The liver tissue oxidative stress enzyme, SOD activity, was significantly decreased in the acetaminophen/paracetamol group results (group 3) compared to the normal control group (group 1), while group 4 activity was relatively similar to the normal control group and group 5 SOD showed relative increased activity compared to the normal control group.

### Histopathological examination of liver tissue sections (Fig. 7)

**Fig. 7:**
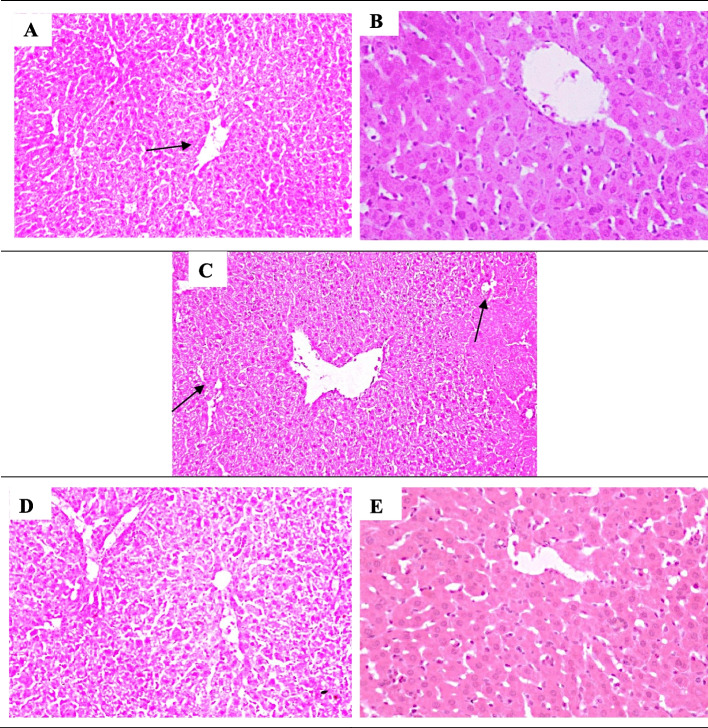
Photo-micrographs of mice liver sections stained by H&E from **A** normal control group 1, demonstrated normal histological structure of organized hepatocytes and lobules (black arrow) (100 ×). **B** DMSO negative control group 2, showed hepatocyte parenchymal features comparable to the normal control (A) (200 ×). **C** Mice treated with acetaminophen/paracetamol as positive control group 3, showing mid-zonal coagulative necrosis of hepatocytes and hyperplasia of Kupffer cells, leukocytic infiltration with intracellular fat droplets arrow (100 ×). **D** mice treated with M33 extract 1b group 4, showed no inflammatory cell infiltrates (100 ×). **E** Mice group treated with M33 extract 1a group 5, demonstrated the normal histological structure of hepatic lobules with mild hepatic sinusoid dilatation (200 ×) [100 × or 200 × magnification]

H&E histopathological examination photo-micrograph of hepatic tissue sections following 14 days assigned for the acute single oral toxicity testing of acetaminophen/paracetamol bio-degradation products. Group 1 (normal control group) and group 2 negative control (DMSO) both showed normal liver cells histological structure (A, B). The positive control group 3 administered the acute oral toxic dose of acetaminophen/paracetamol, revealing moderate dilatation of the hepatic central vein and hepatic sinusoids, in addition to moderate inflammatory changes (grade 2) with lymphocytes and macrophage infiltration and few numbers of neutrophils. Fatty degeneration of the peripheral zone which appeared as intracellular fat droplets. Mid-zonal coagulative necrosis of hepatocytes and hyperplasia of Kupffer cells were all seen (grade IV) (C). In group 4 (M33 extract 1b), there is a mild vascular change compared to the normal group, but not inflammatory (D). Group 5 (M33 extract 1a) showed normal histological structure of hepatic lobules with mild dilatated hepatic sinusoids, but, no markers of inflammation (E).

## Discussion

This study aims to test how to keep the water and crop environment clean from acetaminophen/paracetamol, which may be present in waste water or soil or food/crops nowadays after extensive use recently during the outbreak of COVID-19. This could be done by “Bioremediation” which is an eco-friendly degrading process to deal with toxic chemicals and trace pharmaceuticals/product waste present in the water environment and may have a toxic impact on human beings [2, 3].

Some bacteria have enzymes whose secondary function is to bio-degrade the “trace pharmaceuticals” they are exposed to. This beneficiary criteria is widely used as a “bioremediation” method for getting rid of these trace pharmaceuticals [1]. Bacterial strains are used that have the ability to degrade one of the emerging organic pollutants acetaminophen/paracetamol to release less toxic products domestically at their first release, before traveling through soil or ground to irrigation water [38].

By literature retrieval, amidase or deaminase, and dioxygenase(s) are the main enzymes [39] that degrade acetaminophen/paracetamol by *Streptomyces* bacteria or any other antimicrobial source, to give smaller, simpler metabolites that could be burnt by Kreb’s cycle.

In the present study, *Streptomyces flavofuscus* (sample M33) and *Streptomyces chrestomyceticus* (sample RS2) can utilize standard acetaminophen/paracetamol, as sources of C and N, for growth, into secondary intermediate metabolites, in the culture media or in wastewater or soil used in the current study.

*Streptomyces* is known and used widely for biotechnology of bio-degradation and bio-remediation and drug products [40].

Identification of the 2 selected strains was done phenotypically using TEM which identified the presence of elongated spores as strong evidence for *Actinomycetes* presence [41]. Additionally, molecular identification was then done using 16s rRNA sequencing. The first strain M33 revealed high similarity to *Streptomyces flavofuscus* strain, with 99.35% identity, and the second strain RS2 had a high similarity to *Streptomyces chrestomyceticus*, with 99.57% identity. The identified, aligned strain nucleotide sequences were compared with st. bacterial strains as *Actinomycetes* isolate sequences available at the NIH genetic sequence database GenBank® (Accessed on January 2022) and turned to be new strains, so we deposited them to the NCBI/Nucleotide/Genebank on February 13th, 2022. However, it is important to emphasize that high similarity is good during relating these organisms to specific species, but they may contain different characteristics, depending on other environmental factors and/or bacterial mutations. *It is noteworthy to mention that the best bacterial isolates were from the Wadi El Natrun district in Egypt.*

Bio-degradation secondary products of acetaminophen/paracetamol which were produced in the biological system of M33 and RS2 bacterial strains were identified using GC/Mass, which explained the presence of more than one bio-degradation product related to acetaminophen/paracetamol, cyanoacetylene, hexadecanol, dodecenamide, octadecanamide, tetrahydrocannabinol, undecanoic acid, isohexyl acrylate, and acetamide, and this was done previously using several kinds of bacteria from different sources either water or soil [12] and reported by scientists [42].

After the prediction of bio-degradation product molecular structure, the solubility and absorption, then distribution, metabolism, and excreation (ADME) as well as the cellular, metabolic, and functional toxicity prediction (PreADMET) was done. LC_Mass was done on the bio-degradation products of the M33 bacterial strain to overture the bio-degradation mechanism/reaction pathway, which was predicted computationally.

The rationale for using shaking or static samples is that the bio-degradation/transformation % was increased in the shaken flask runs, with the help of the growth medium formulation’s optimization for C/N ratio and type of nitrogen sources, growth temperature 30°C/not higher and pH 7.2. This shaking state helped to reduce the harmful by-product(s) formation as well as mentioned in the review article by Ferraiuolo et al. in 2021 [40] confirmed by less toxicity prediction as well as in vivo and in vitro tested toxicity studies.

Taking into consideration that the bio-degradation product compounds could be further studied as new treatment modalities, they displayed good acceptable anti-microbial activity and cytotoxic bioactivity as previously reported in other studies [12, 42–44].

The acetaminophen/paracetamol bio-degradation products downstream targets were predicted chemo-informatically using the PreADMET database that identified these target(s) enzymes, receptors, or cytosolic and membrane proteins (Table 4).

Degradation products of the M33 sample bacterial stain were tested for the possible antimicrobial activity in the 2 samples during static and shaking conditions, showing high antibacterial activity on *S. arues*; during the static phase, showing high antibacterial activity against *E. coli*; and during static and shaking phases, showing high antimicrobial on *MRSA* if the bio-degradation product sample was produced with shaking.

Via the chemo-informatics/bioinformatics tool(s), the pharmacogenomics knowledge resource, PharmGKB® [18] (accessed on November 3, 2022), identified the small organic molecule acetaminophen/paracetamol-waste (APAP; *N*-acetyl-para-aminophenol) chemical structure and how it affects biological system(s), followed by the identification of each of the bio-degradation products from various samples.

One of the study objectives is to prove that these bio-degradation products have a lower toxic effect(s) when being compared to acetaminophen/paracetamol after the majority are predicted safe computationally. So, we performed the in vitro MTT cytotoxicity assay using M33 and RS2 bio-degradation products on HepG2 and MCF7 cell lines. There is a significant increase in bio-degradation products IC50 [32] compared to acetaminophen/paracetamol for the 2 cell lines HepG2 and MCF7, which means that acetaminophen/paracetamol has a higher toxicity level than all bio-degradation products on both cell lines (bio-degradation products are safer).

Moreover, in vivo acute toxicity of M33 sample bio-degradation products was tested orally using female Swiss albino with about 20 g body weight and 20 weeks of age CD1 mice, with histopathological confirmation of hepatic tissue preservation after 14 days of the experiment duration.

Acetaminophen/paracetamol acute single-dose oral hepatotoxicity was characterized by OS [45] and decreased antioxidant enzymes SOD and CAT and perturbed TAC in cases of toxicity. Caspase-9 and IL-6 levels in liver tissue were done to monitor apoptosis and inflammation processes, respectively.

Finally, hepatic tissue H&E histological during the acute single-dose oral toxicity testing [46] examinations confirmed LFT’s findings as safe compounds.

It is noteworthy to mention that the current study’s main goal to bio-degrade acetaminophen/paracetamol to non-toxic or less toxic bio-degradation compounds was achieved, using bacteria isolated from the near environment. Moreover, the 2 novel isolated bacterial strains transformed acetaminophen/paracetamol into safe non-toxic secondary intermediate products with potential biological activity (strength and novelty of the study).

## Summary and conclusions

After isolation of several bacterial strains from different Egyptian habitats (desert, lake water, and soil samples), 2 novel strains with the ability to bio-degrade a standard amount of acetaminophen/paracetamol® presenting “trace pharmaceuticals” in our drinking water to less toxic/safe bio-degradation products as well as being potentially biologically active, have good anti-microbial potential that mandates further experimental validation. We deposited these 2 novel strains to the NCBI GeneBank on Feb. 13th, 2022, after characterization and identification.

These bio-degradation product chemical structures and properties were identified and characterized experimentally and by cheminformatics in silico testing.

### Limitation and future perspective

The exact biological mechanism of the novel *Streptomyces* bacterial strains would exhibit more characterization(s) if they could prove chemotherapeutic effects [47] based on the in silico predicted downstream target enzymes or membrane proteins/receptors.

### Recommendation

Identify if these novel *Streptomyces* bacterial stains can degrade estrogenic waste, a study to be conducted on both the cell and target levels.

Therefore, the need for proper bio-degradation of “trace pharmaceuticals” at its first domestic release is mandatory for better health achievement. This would be achieved via a “national task-force initiative” for environment-friendly disposal of unused medication to stay out of our drinking water and/or cultivated crops.

### Supplementary Information

**Additional file 1: Fig. S1.** Acetaminophen/paracetamol bio-degradation proposed whole pathway/reaction diagram retrieved from EAWAG-BBD pathway map starting with reaction r1629 (Accessed Feb. 16^th^, 2023). http://eawag-bbd.ethz.ch/servlets/pageservlet?ptype=r&reacID=r1629. **Fig. S2.** HepG2 and MCF7 IHC photo-micrographs imaged by an Inverted Microscope. Morphological changes visualization in cancer cell lines by Inverted Microscopy (Phase Contrast) to confirm apoptotic cells morphological alterations of shrinkage, nuclear condensation, and fragmentation, and apoptotic bodies formation as well as loss of attachment to neighboring cells, all confirming apoptosis. **Table S2.** Effect of acetaminophen acute single oral dose (200 mg/k.g BW) and the acetaminophen bio-degradation products IC50 on blood liver function tests, liver tissue oxidative stress markers (SOD and MDA), liver tissue antioxidant levels (TAC and CAT) as well as liver IL-6 and caspase-9, as an in vivo acute single oral toxicity test.

## Data Availability

All the source code and data are available within the manuscript. Any further inquiries are to be sent to the corresponding author.

## Abbreviations

*A. niger*: *Aspergillus niger*
ADMET: Absorption, distribution, metabolism, excretion—toxicity
ALT: Alanine aminotransferase
ANOVA: Analysis of variance
APAP: Acetaminophen
AST: Aspartate aminotransferase
*B. subtilus*: *Bacillus subtilus*
BBD: Bio-catalysis/bio-degradation database
BLAST: Basic local alignment search tool
BW: Body weight
*C. albicans*: *Candida albicans*
CAT: Catalase
CNS: Central nervous system
DB: Database
D.F.: Degrees of freedom
DMSO: Dimethyl sulfoxide
DNA: Deoxyribonucleic acid
*E. coli*: *Escherichia coli*
EAWAG: Swiss Federal Institute for Environmental Science and Technology
ELISA: Enzyme-linked immunosorbent assay
ESI: Electrospray ionization
FBS: Fetal bovine serum
FLEX: Flexibility
GC/EI-MS: Gas chromatography/electron ionization/mass spectrometry
GC/MS: Gas chromatography-mass spectrometry
Geldoc-it: Gel documentation system
GGT: Gamma-glutamyl transferase
HBA: Number of hydrogen bond acceptors
HBD: Number of hydrogen bond donors
HepG2: Human liver cancer cell line
hERG: Human ether-à-go-go-related gene cardiac potassium channel
HPLC: High-performance liquid chromatography
IC50: The half maximal inhibitory concentration.
IL6: Interleukin-6
INSATU: Saturation
INSOLU: Solubility
IUPAC: International Union of Pure and Applied Chemistry
LB: Lysogeny broth
LC/MS: Liquid chromatography/mass spectrometry
LIPO: Lipophilicity
log p: The logarithm of the partition coefficient
MCF7: Human breast cancer cell line
MDA: Malondialdehyde
MRSA: Multidrug-resistant *Staphylococcus aureus*
MSM: Mineral salt medium
MTT: 3-(4, 5-Di-MethylThiazol-2-yl)-2,5-diphenyl tetrazolium bromide
MW: Molecular weight
NCBI: National Center for Biotechnology Information
NIH: National Institute of Health
NIST: National Institute of Standards and Technology
NMR: Nuclear magnetic resonance
OTC: Over-the-counter
PCR: Polymerase chain reaction
PDM: Pairwise distance method
PharmGKB: Pharmacogenomics knowledge resource
Ppm: Part per million
RPA: Relative peak area
RPMI medium: Roswell Park Memorial Institute Medium
rRNA: Ribosomal ribonucleic acid.
RRT: Relative retention time
RT: Retention time
*S. typhi*: *Salmonella typhi*
*S. ureus*: *Staphylococcus aureus*
SDGs: Sustainable Development Goals
SH: Shaking condition (shaking flask)
SIB: Swiss Institute of Bioinformatics
SMILES: Simplified Molecular-Input Line-Entry System
SOD: Superoxide dismutase
SPSS: Statistical package for social studies
ST: Static condition (static flask)
TAC: Total antioxidant capacity
TBAR: Thiobarbituric acid reactive
TEM: Transmission electron microscope
TPSA: Topological polar surface area
