# Supplementary material for: Acetaminophen-traces bioremediation with novel phenotypically and genotypically characterized 2 Streptomyces strains using chemo-informatics, in vivo, and in vitro experiments for cytotoxicity and biological activity
Source: J Genet Eng Biotechnol. 2023 Dec 19;21:171. doi: 10.1186/s43141-023-00602-w (PMC10730784; doi:10.1186/s43141-023-00602-w)
Supplement: Supplementary file 1 — Additional file 1: Fig. S1. Acetaminophen/paracetamol bio-degradation proposed whole pathway/reaction diagram retrieved from EAWAG-BBD pathway map starting with reaction r1629 (Accessed Feb. 16th, 2023). http://eawag-bbd.ethz.ch/servlets/pageservlet?ptype=r&reacID=r1629. Fig. S2. HepG2 and MCF7 IHC photo-micrographs imaged by an Inverted Microscope. Morphological changes visualization in cancer cell lines by Inverted Microscopy (Phase Contrast) to confirm apoptotic cells morphological alterations of shrinkage, nuclear condensation, and fragmentation, and apoptotic bodies formation as well as loss of attachment to neighboring cells, all confirming apoptosis. Table S2. Effect of acetaminophen acute single oral dose (200 mg/k.g BW) and the acetaminophen bio-degradation products IC50 on blood liver function tests, liver tissue oxidative stress markers (SOD and MDA), liver tissue antioxidant levels (TAC and CAT) as well as liver IL-6 and caspase-9, as an in vivo acute single oral toxicity test. [file 43141_2023_602_MOESM1_ESM.docx]

**Title:**

**Acetaminophen-traces Bioremediation using Novel Phenotypically/Genotypically-characterized Strains, Chemo-informatics & experiment tested for Cytotoxicity & Biological Activity**

| 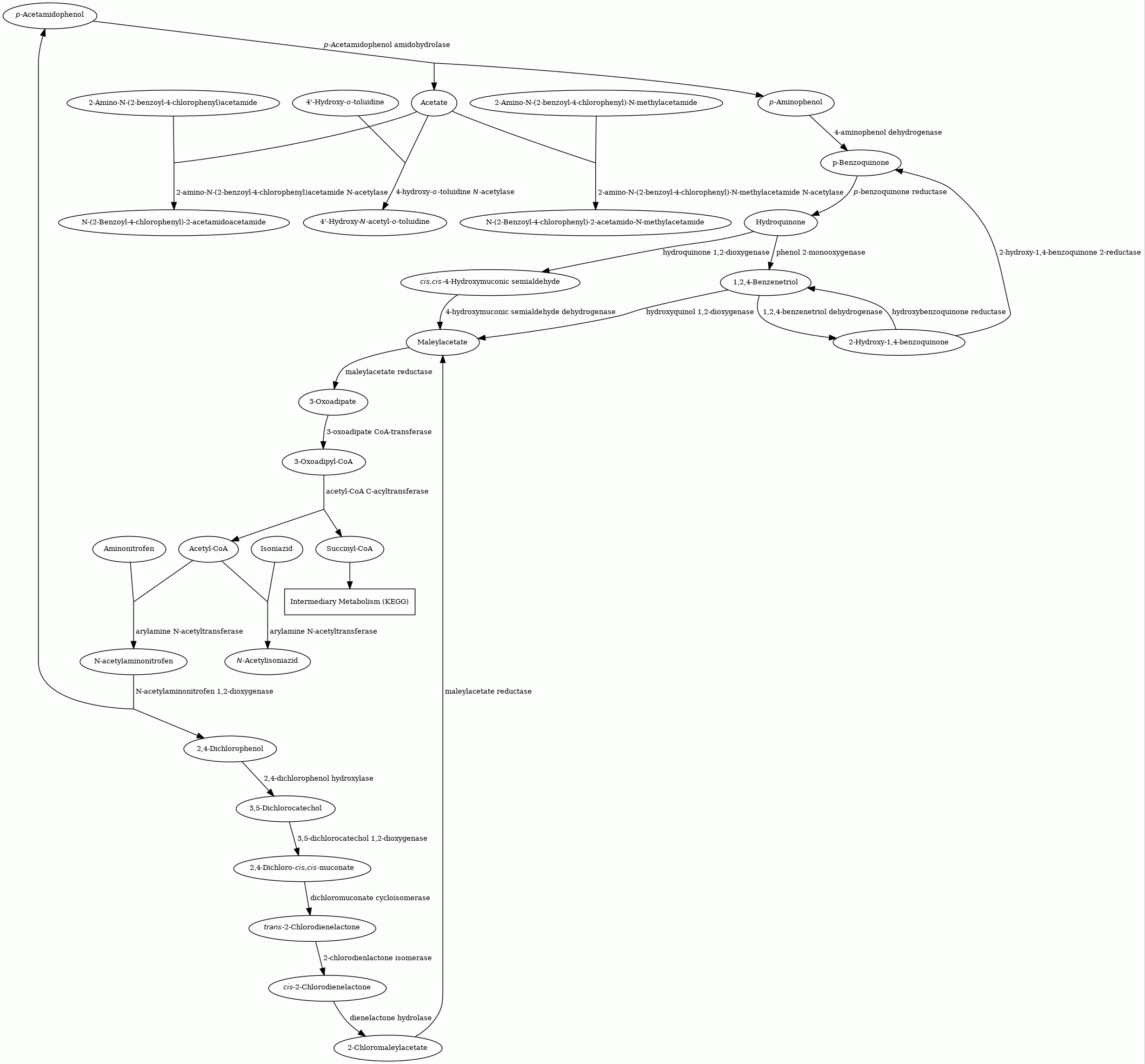 |
| --- |

**Supplementary Figure S1: Acetaminophen/paracetamol bio-degradation proposed whole pathway/reaction diagram retrieved from EAWAG-BBD pathway map starting with reaction r1629** (Accessed Feb. 16^th^, 2023). <http://eawag-bbd.ethz.ch/servlets/pageservlet?ptype=r&reacID=r1629>

**Specific gene detection technique: 16S rRNA gene region of Sample A**


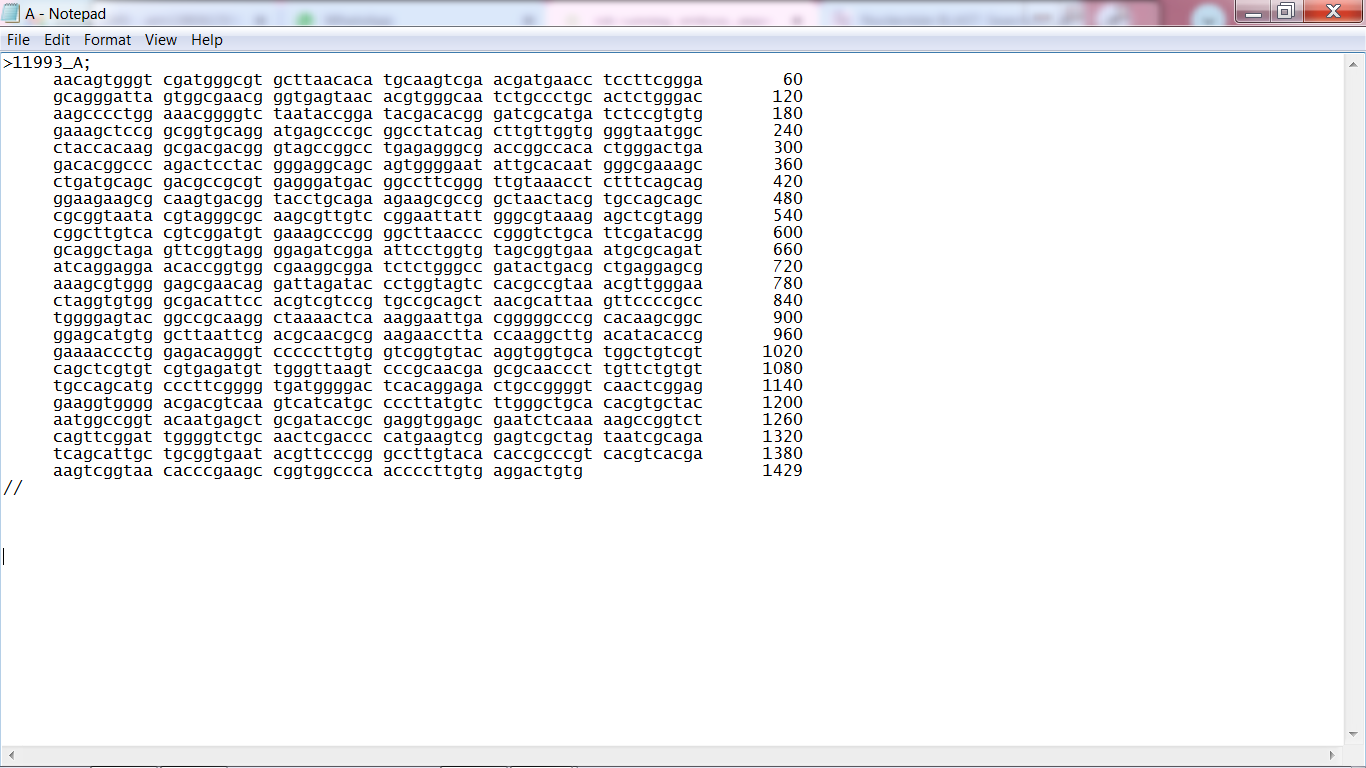


**Alignments data for 16S rRNA gene region sequence of Sample A**


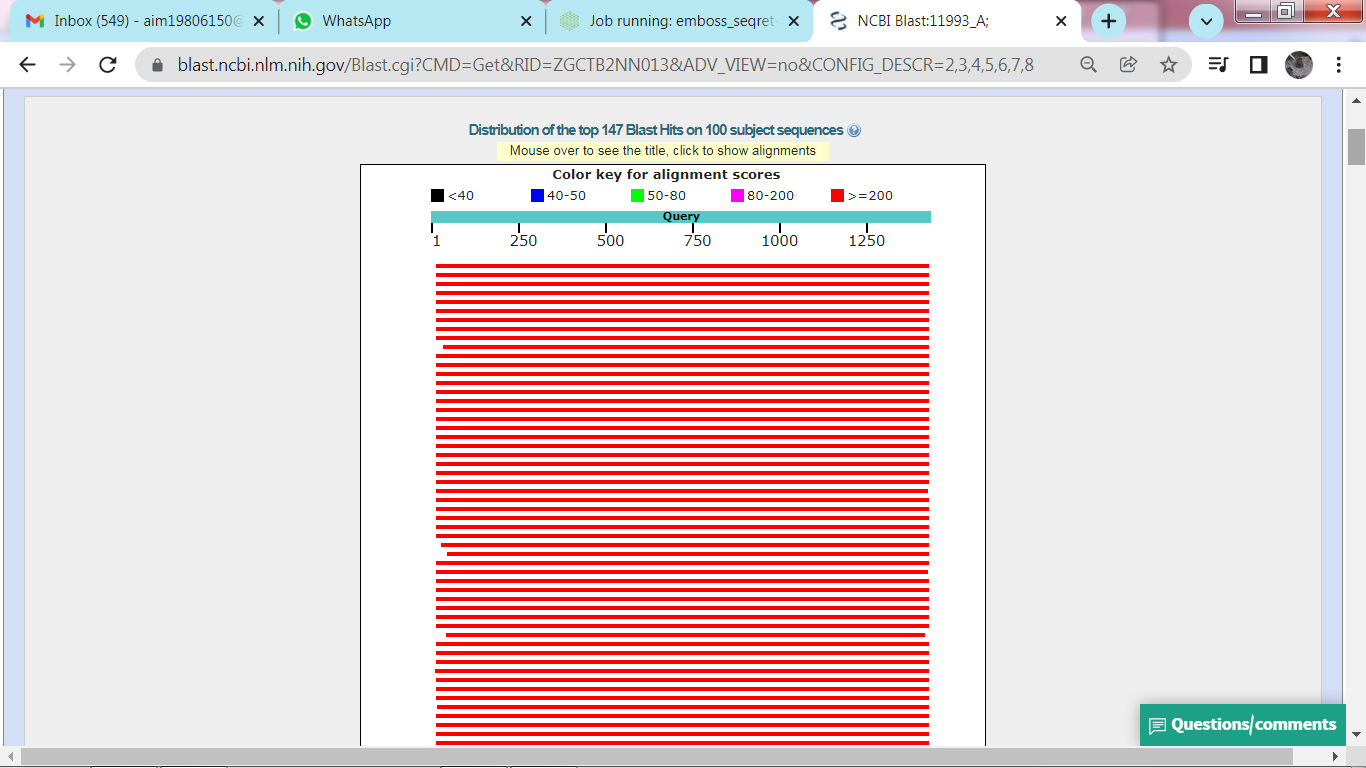


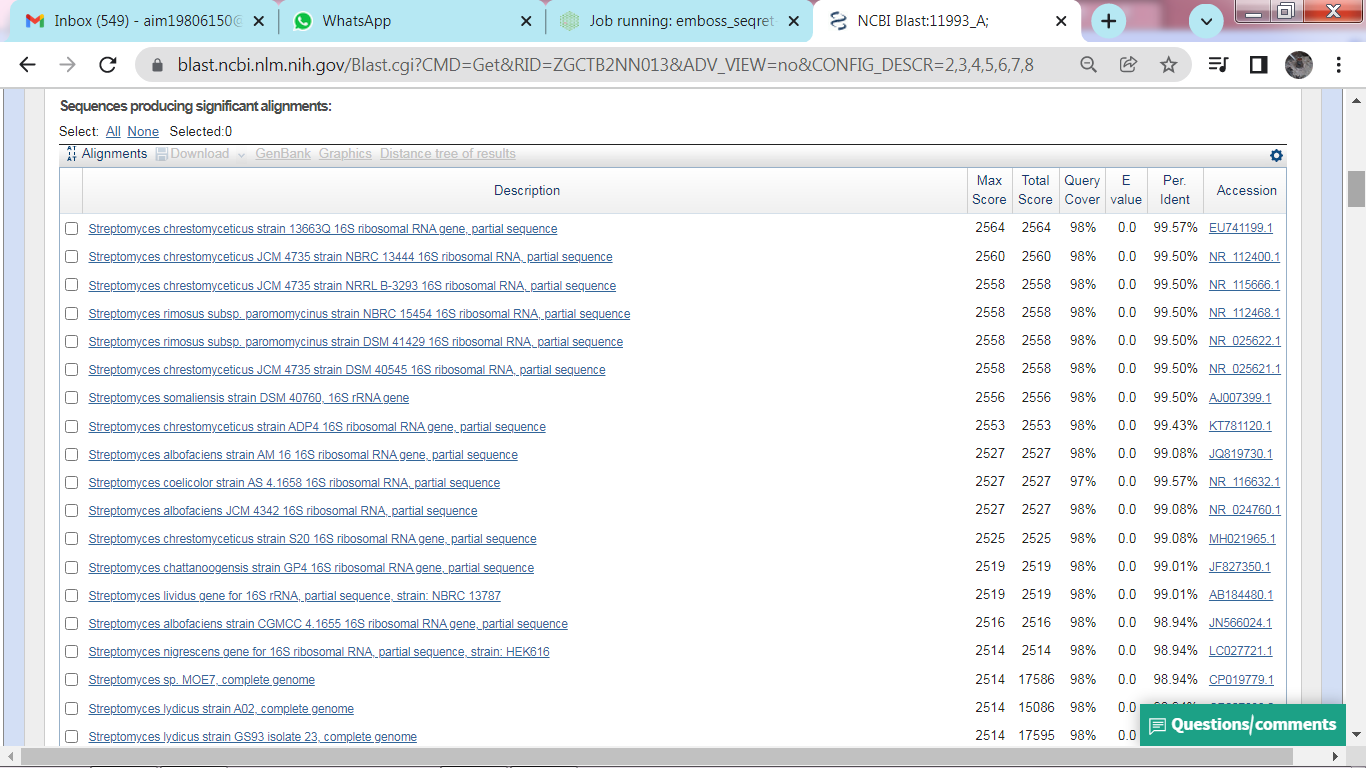


**16S rRNA gene region of Sample B.**


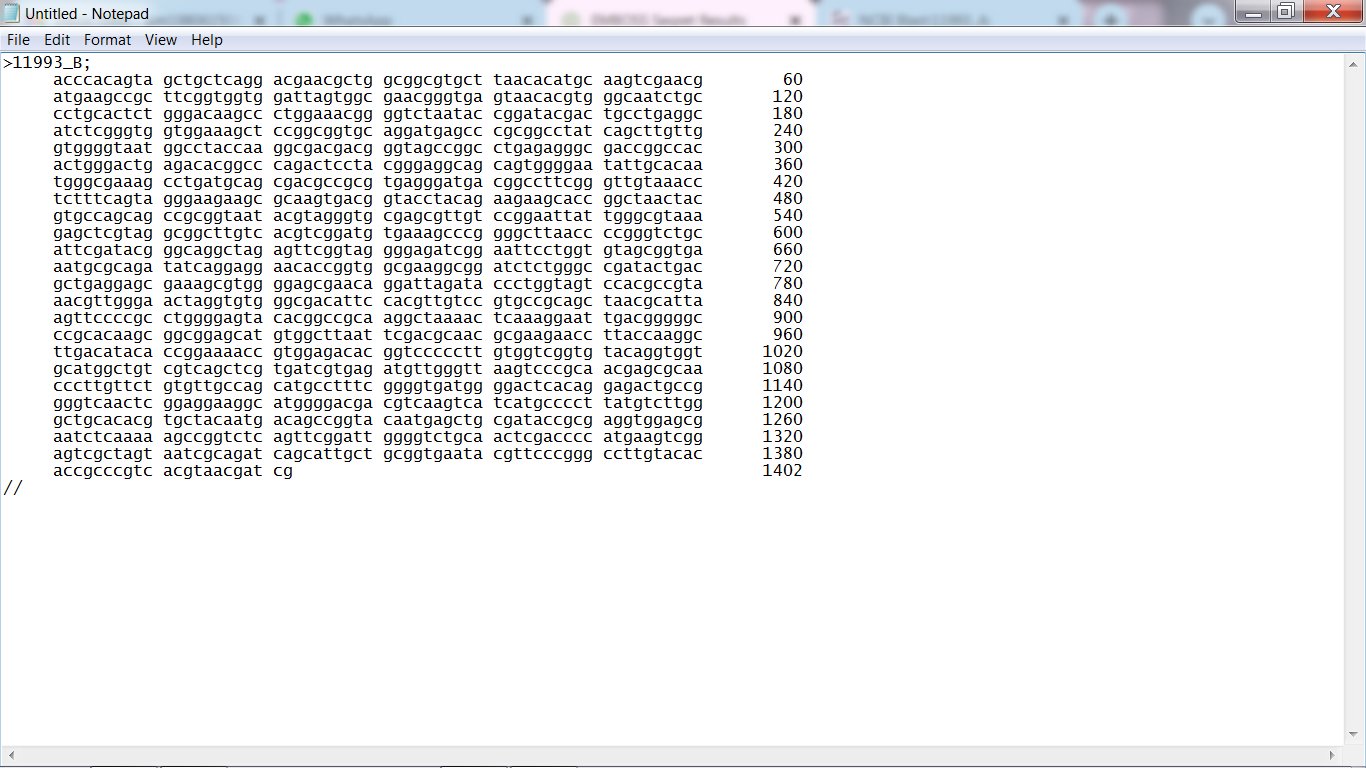


**Alignments data for 16S rRNA gene region sequence of Sample B.**


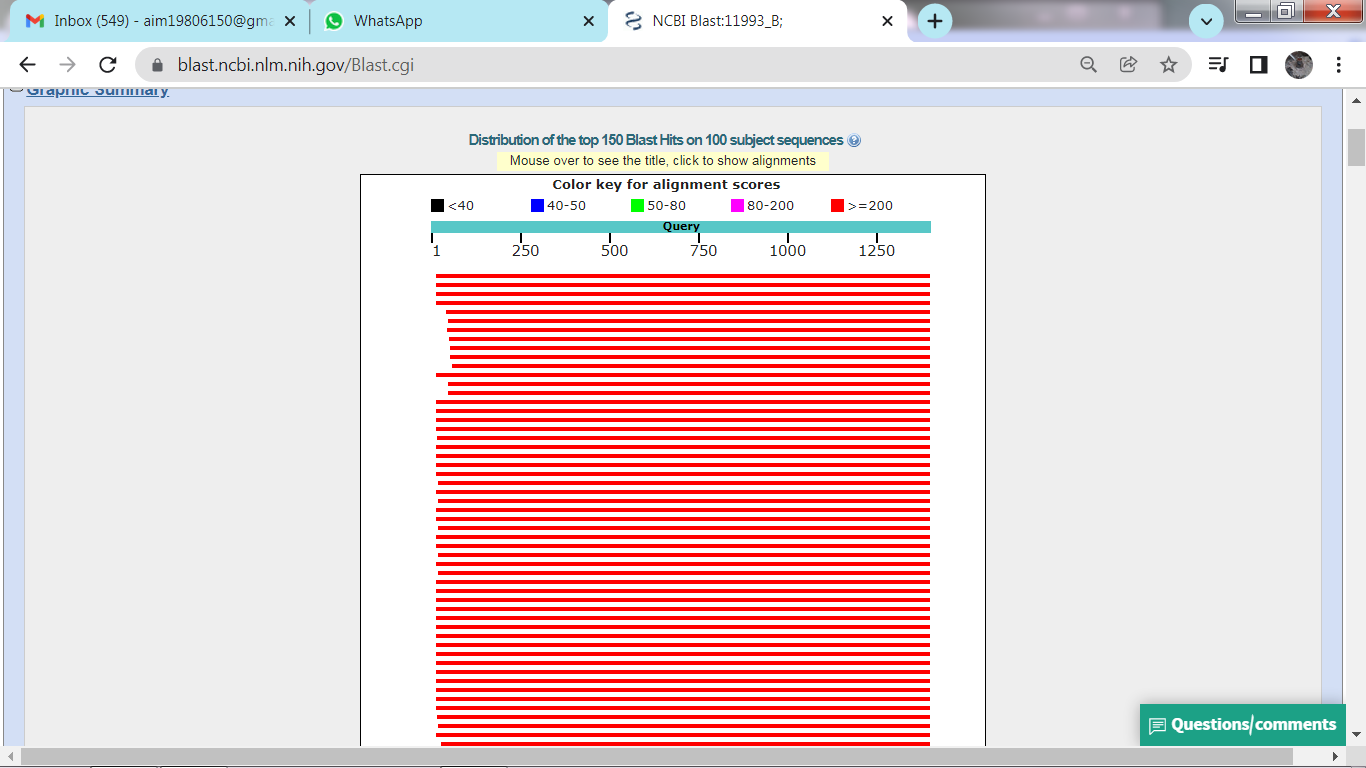


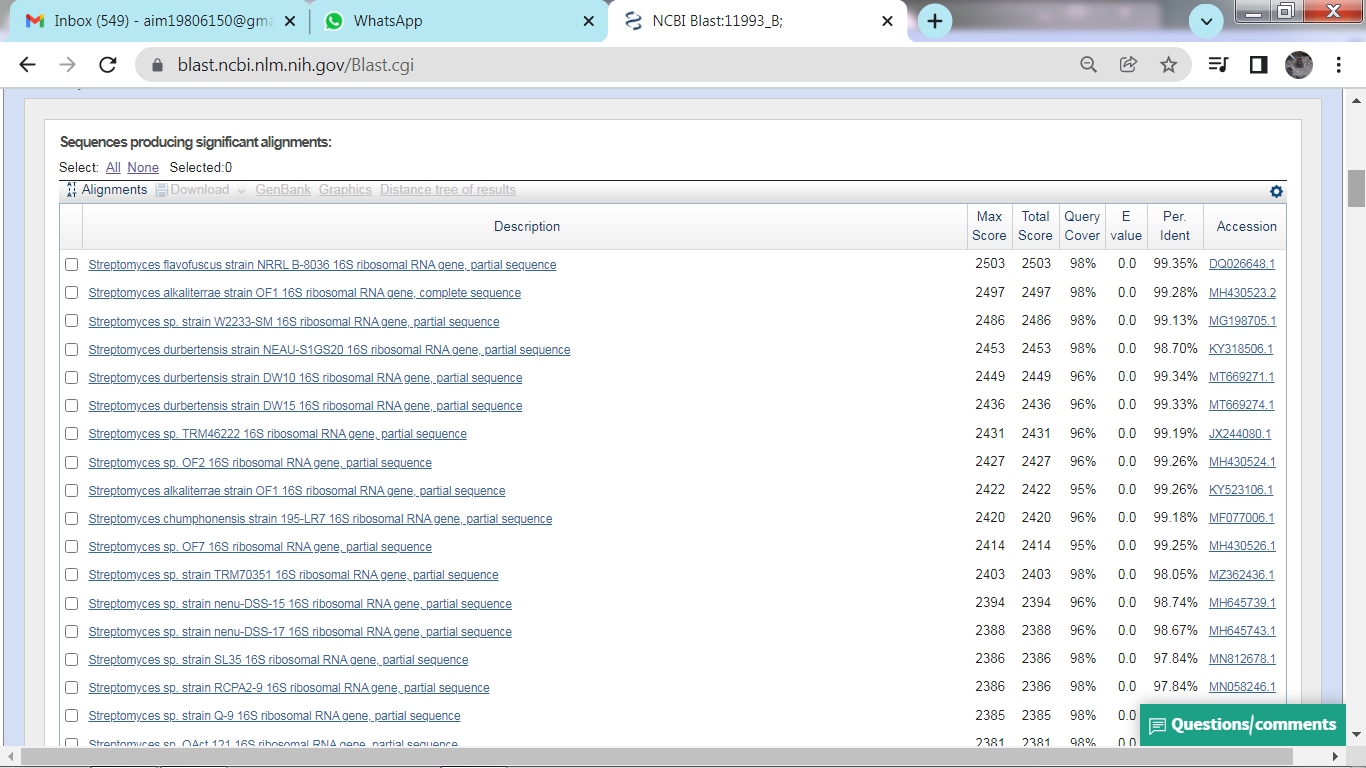


16S rRNA gene region sequence of five Samples..

>11993_B;

acccacagtagctgctcaggacgaacgctggcggcgtgcttaacacatgcaagtcgaacg

atgaagccgcttcggtggtggattagtggcgaacgggtgagtaacacgtgggcaatctgc

cctgcactctgggacaagccctggaaacggggtctaataccggatacgactgcctgaggc

atctcgggtggtggaaagctccggcggtgcaggatgagcccgcggcctatcagcttgttg

gtggggtaatggcctaccaaggcgacgacgggtagccggcctgagagggcgaccggccac

actgggactgagacacggcccagactcctacgggaggcagcagtggggaatattgcacaa

tgggcgaaagcctgatgcagcgacgccgcgtgagggatgacggccttcgggttgtaaacc

tctttcagtagggaagaagcgcaagtgacggtacctacagaagaagcaccggctaactac

gtgccagcagccgcggtaatacgtagggtgcgagcgttgtccggaattattgggcgtaaa

gagctcgtaggcggcttgtcacgtcggatgtgaaagcccggggcttaaccccgggtctgc

attcgatacgggcaggctagagttcggtaggggagatcggaattcctggtgtagcggtga

aatgcgcagatatcaggaggaacaccggtggcgaaggcggatctctgggccgatactgac

gctgaggagcgaaagcgtggggagcgaacaggattagataccctggtagtccacgccgta

aacgttgggaactaggtgtgggcgacattccacgttgtccgtgccgcagctaacgcatta

agttccccgcctggggagtacacggccgcaaggctaaaactcaaaggaattgacgggggc

ccgcacaagcggcggagcatgtggcttaattcgacgcaacgcgaagaaccttaccaaggc

ttgacatacaccggaaaaccgtggagacacggtcccccttgtggtcggtgtacaggtggt

gcatggctgtcgtcagctcgtgatcgtgagatgttgggttaagtcccgcaacgagcgcaa

cccttgttctgtgttgccagcatgcctttcggggtgatggggactcacaggagactgccg

gggtcaactcggaggaaggcatggggacgacgtcaagtcatcatgccccttatgtcttgg

gctgcacacgtgctacaatgacagccggtacaatgagctgcgataccgcgaggtggagcg

aatctcaaaaagccggtctcagttcggattggggtctgcaactcgaccccatgaagtcgg

agtcgctagtaatcgcagatcagcattgctgcggtgaatacgttcccgggccttgtacac

accgcccgtcacgtaacgatcg

>11993_A;

aacagtgggtcgatgggcgtgcttaacacatgcaagtcgaacgatgaacctccttcggga

gcagggattagtggcgaacgggtgagtaacacgtgggcaatctgccctgcactctgggac

aagcccctggaaacggggtctaataccggatacgacacgggatcgcatgatctccgtgtg

gaaagctccggcggtgcaggatgagcccgcggcctatcagcttgttggtggggtaatggc

ctaccacaaggcgacgacgggtagccggcctgagagggcgaccggccacactgggactga

gacacggcccagactcctacgggaggcagcagtggggaatattgcacaatgggcgaaagc

ctgatgcagcgacgccgcgtgagggatgacggccttcgggttgtaaacctctttcagcag

ggaagaagcgcaagtgacggtacctgcagaagaagcgccggctaactacgtgccagcagc

cgcggtaatacgtagggcgcaagcgttgtccggaattattgggcgtaaagagctcgtagg

cggcttgtcacgtcggatgtgaaagcccggggcttaaccccgggtctgcattcgatacgg

gcaggctagagttcggtaggggagatcggaattcctggtgtagcggtgaaatgcgcagat

atcaggaggaacaccggtggcgaaggcggatctctgggccgatactgacgctgaggagcg

aaagcgtggggagcgaacaggattagataccctggtagtccacgccgtaaacgttgggaa

ctaggtgtgggcgacattccacgtcgtccgtgccgcagctaacgcattaagttccccgcc

tggggagtacggccgcaaggctaaaactcaaaggaattgacgggggcccgcacaagcggc

ggagcatgtggcttaattcgacgcaacgcgaagaaccttaccaaggcttgacatacaccg

gaaaaccctggagacagggtcccccttgtggtcggtgtacaggtggtgcatggctgtcgt

cagctcgtgtcgtgagatgttgggttaagtcccgcaacgagcgcaacccttgttctgtgt

tgccagcatgcccttcggggtgatggggactcacaggagactgccggggtcaactcggag

gaaggtggggacgacgtcaagtcatcatgccccttatgtcttgggctgcacacgtgctac

aatggccggtacaatgagctgcgataccgcgaggtggagcgaatctcaaaaagccggtct

cagttcggattggggtctgcaactcgaccccatgaagtcggagtcgctagtaatcgcaga

tcagcattgctgcggtgaatacgttcccgggccttgtacacaccgcccgtcacgtcacga

aagtcggtaacacccgaagccggtggcccaaccccttgtgaggactgtgBacccacagta

gctgctcaggacgaacgctggcggcgtgcttaacacatgcaagtcgaacgatgaagccgc

ttcggtggtggattagtggcgaacgggtgagtaacacgtgggcaatctgccctgcactct

gggacaagccctggaaacggggtctaataccggatacgactgcctgaggcatctcgggtg

gtggaaagctccggcggtgcaggatgagcccgcggcctatcagcttgttggtggggtaat

ggcctaccaaggcgacgacgggtagccggcctgagagggcgaccggccacactgggactg

agacacggcccagactcctacgggaggcagcagtggggaatattgcacaatgggcgaaag

cctgatgcagcgacgccgcgtgagggatgacggccttcgggttgtaaacctctttcagta

gggaagaagcgcaagtgacggtacctacagaagaagcaccggctaactacgtgccagcag

ccgcggtaatacgtagggtgcgagcgttgtccggaattattgggcgtaaagagctcgtag

gcggcttgtcacgtcggatgtgaaagcccggggcttaaccccgggtctgcattcgatacg

ggcaggctagagttcggtaggggagatcggaattcctggtgtagcggtgaaatgcgcaga

tatcaggaggaacaccggtggcgaaggcggatctctgggccgatactgacgctgaggagc

gaaagcgtggggagcgaacaggattagataccctggtagtccacgccgtaaacgttggga

actaggtgtgggcgacattccacgttgtccgtgccgcagctaacgcattaagttccccgc

ctggggagtacacggccgcaaggctaaaactcaaaggaattgacgggggcccgcacaagc

ggcggagcatgtggcttaattcgacgcaacgcgaagaaccttaccaaggcttgacataca

ccggaaaaccgtggagacacggtcccccttgtggtcggtgtacaggtggtgcatggctgt

cgtcagctcgtgatcgtgagatgttgggttaagtcccgcaacgagcgcaacccttgttct

gtgttgccagcatgcctttcggggtgatggggactcacaggagactgccggggtcaactc

ggaggaaggcatggggacgacgtcaagtcatcatgccccttatgtcttgggctgcacacg

tgctacaatgacagccggtacaatgagctgcgataccgcgaggtggagcgaatctcaaaa

agccggtctcagttcggattggggtctgcaactcgaccccatgaagtcggagtcgctagt

aatcgcagatcagcattgctgcggtgaatacgttcccgggccttgtacacaccgcccgtc

acgtaacgatcg

>EU741199.1 Streptomyces chrestomyceticus strain 13663Q 16S ribosomal RNA gene, partial sequence

CTGGCTCAGGACGAACGCTGGCGGCGTGCTTAACACATGCAAGTCGAACGATGAACCTCC

TTCGGGAGGGGATTAGTGGCGAACGGGTGAGTAACACGTGGGCAATCTGCCCTGCACTCT

GGGACAAGCCCTGGAAACGGGGTCTAATACCGGATACGACACGGGATCGCATGATCTCCG

TGTGGAAAGCTCCGGCGGTGCAGGATGAGCCCGCGGCCTATCAGCTTGTTGGTGGGGTAA

TGGCCTACCAAGGCGACGACGGGTAGCCGGCCTGAGAGGGCGACCGGCCACACTGGGACT

GAGACACGGCCCAGACTCCTACGGGAGGCAGCAGTGGGGAATATTGCACAATGGGCGAAA

GCCTGATGCAGCGACGCCGCGTGAGGGATGACGGCCTTCGGGTTGTAAACCTCTTTCAGC

AGGGAAGAAGCGCAAGTGACGGTACCTGCAGAAGAAGCGCCGGCTAACTACGTGCCAGCA

GCCGCGGTAATACGTAGGGCGCAAGCGTTGTCCGGAATTATTGGGCGTAAAGAGCTCGTA

GGCGGCTTGTCACGTCGGATGTGAAAGCCCGGGGCTTAACCCCGGGTCTGCATTCGATAC

GGGCAGGCTAGAGTTCGGTAGGGGAGATCGGAATTCCTGGTGTAGCGGTGAAATGCGCAG

ATATCAGGAGGAACACCGGTGGCGAAGGCGGATCTCTGGGCCGATACTGACGCTGAGGAG

CGAAAGCGTGGGGAGCGAACAGGATTAGATACCCTGGTAGTCCACGCCGTAAACGTTGGG

AACTAGGTGTGGGCGACATTCCACGTCGTCCGTGCCGCAGCTAACGCATTAAGTTCCCCG

CCTGGGGAGTACGGCCGCAAGGCTAAAACTCAAAGGAATTGACGGGGGCCCGCACAAGCG

GCGGAGCATGTGGCTTAATTCGACGCAACGCGAAGAACCTTACCAAGGCTTGACATACAC

CGGAAAACCCTGGAGACAGGGTCCCCCTTGTGGTCGGTGTACAGGTGGTGCATGGCTGTC

GTCAGCTCGTGTCGTGAGATGTTGGGTTAAGTCCCGCAACGAGCGCAACCCTTGTTCTGT

GTTGCCAGCATGCCCTTCGGGGTGATGGGGACTCACAGGAGACTGCCGGGGTCAACTCGG

AGGAAGGTGGGGACGACGTCAAGTCATCATGCCCCTTATGTCTTGGGCTGCACACGTGCT

ACAATGGCCGGTACAATGAGCTGCGATACCGCGAGGTGGAGCGAATCTCAAAAAGCCGGT

CTCAGTTCGGATTGGGGTCTGCAACTCGACCCCATGAAGTCGGAGTCGCTAGTAATCGCA

GATCAGCATTGCTGCGGTGAATACGTTCCCGGGCCTTGTACACACCGCCCGTCACGTCAC

GAAAGTCGGTAACACCCGAAGCCGGTGGCCCAACCCCTTGTGGGAGGGAATCGTCGAAGG

TGGGACTGGCGATTGGGACGAAGTCGTAACAAGGTAGCCGTACCGGAAGGTGCGGCTGG

>NR_112400.1 Streptomyces chrestomyceticus JCM 4735 strain NBRC 13444 16S ribosomal RNA, partial sequence

ACGAACGCTGGCGGCGTGCTTAACACATGCAAGTCGAACGATGAACCTCCTTCGGGAGGG

GATTAGTGGCGAACGGGTGAGTAACACGTGGGCAATCTGCCCTGCACTCTGGGACAAGCC

CTGGAAACGGGGTCTAATACCGGATACGACACGGGATCGCATGATCTCCGTGTGGAAAGC

TCCGGCGGTGCAGGATGAGCCCGCGGCCTATCAGCTTGTTGGTGGGGTAATGGCCTACCA

AGGCGACGACGGGTAGCCGGCCTGAGAGGGCGACCGGCCACACTGGGACTGAGACACGGC

CCAGACTCCTACGGGAGGCAGCAGTGGGGAATATTGCACAATGGGCGNAAGCCTGATGCA

GCGACGCCGCGTGAGGGATGACGGCCTTCGGGTTGTAAACCTCTTTCAGCAGGGAAGAAG

CGCAAGTGACGGTACCTGCAGAAGAAGCGCCGGCTAACTACGTGCCAGCAGCCGCGGTAA

TACGTAGGGCGCAAGCGTTGTCCGGAATTATTGGGCGTAAAGAGCTCGTAGGCGGCTTGT

CACGTCGGATGTGAAAGCCCGGGGCTTAACCCCGGGTCTGCATTCGATACGGGCAGGCTA

GAGTTCGGTAGGGGAGATCGGAATTCCTGGTGTAGCGGTGAAATGCGCAGATATCAGGAG

GAACACCGGTGGCGAAGGCGGATCTCTGGGCCGATACTGACGCTGAGGAGCGAAAGCGTG

GGGAGCGAACAGGATTAGATACCCTGGTAGTCCACGCCGTAAACGTTGGGAACTAGGTGT

GGGCGACATTCCACGTCGTCCGTGCCGCAGCTAACGCATTAAGTTCCCCGCCTGGGGAGT

ACGGCCGCAAGGCTAAAACTCAAAGGAATTGACGGGGGCCCGCACAAGCGGCGGAGCATG

TGGCTTAATTCGACGCAACGCGAAGAACCTTACCAAGGCTTGACATACACCGGAAAACCC

TGGAGACAGGGTCCCCCTTGTGGTCGGTGTACAGGTGGTGCATGGCTGTCGTCAGCTCGT

GTCGTGAGATGTTGGGTTAAGTCCCGCAACGAGCGCAACCCTTGTTCTGTGTTGCCAGCA

TGCCCTTCGGGGTGATGGGGACTCACAGGAGACTGCCGGGGTCAACTCGGAGGAAGGTGG

GGACGACGTCAAGTCATCATGCCCCTTATGTCTTGGGCTGCACACGTGCTACAATGGCCG

GTACAATGAGCTGCGATACCGCGAGGTGGAGCGAATCTCAAAAAGCCGGTCTCAGTTCGG

ATTGGGGTCTGCAACTCGACCCCATGAAGTCGGAGTCGCTAGTAATCGCAGATCAGCATT

GCTGCGGTGAATACGTTCCCGGGCCTTGTACACACCGCCCGTCACGTCACGAAAGTCGGT

AACACCCGAAGCCGGTGGCCCAACCCCTTGTGGGAGGGAATCGTCGAAGGTGGGACTGGC

GATTGGGACGAAGTCGTAACAAGGTAGCCGTACCGGAAGG

>NR_025622.1 Streptomyces rimosus subsp. paromomycinus strain DSM 41429 16S ribosomal RNA, partial sequence

AACGCTGGCGGCGTGCTTAACACATGCAAGTCGAACGATGAACCTCCTTCGGGAGGGGAT

TAGTGGCGAACGGGTGAGTAACACGTGGGCAATCTGCCCTGCACTCTGGGACAAGCCCTG

GAAACGGGGTCTAATACCGGATACGACACGGGATCGCATGATCTCCGTGTGGAAAGCTCC

GGCGGTGCAGGATGAGCCCGCGGCCTATCAGCTTGTTGGTGGGGTAATGGCCTACCAAGG

CGACGACGGGTAGCCGGCCTGAGAGGGCGACCGGCCACACTGGGACTGAGACACGGCCCA

GACTCCTACGGGAGGCAGCAGTGGGGAATATTGCACAATGGGCGCAAGCCTGATGCAGCG

ACGCCGCGTGAGGGATGACGGCCTTCGGGTTGTAAACCTCTTTCAGCAGGGAAGAAGCGC

AAGTGACGGTACCTGCAGAAGAAGCGCCGGCTAACTACGTGCCAGCAGCCGCGGTAATAC

GTAGGGCGCAAGCGTTGTCCGGAATTATTGGGCGTAAAGAGCTCGTAGGCGGCTTGTCAC

GTCGGATGTGAAAGCCCGGGGCTTAACCCCGGGTCTGCATTCGATACGGGCAGGCTAGAG

TTCGGTAGGGGAGATCGGAATTCCTGGTGTAGCGGTGAAATGCGCAGATATCAGGAGGAA

CACCGGTGGCGAAGGCGGATCTCTGGGCCGATACTGACGCTGAGGAGCGAAAGCGTGGGG

AGCGAACAGGATTAGATACCCTGGTAGTCCACGCCGTAAACGTTGGGAACTAGGTGTGGG

CGACATTCCACGTCGTCCGTGCCGCAGCTAACGCATTAAGTTCCCCGCCTGGGGAGTACG

GCCGCAAGGCTAAAACTCAAAGGAATTGACGGGGGCCCGCACAAGCGGCGGAGCATGTGG

CTTAATTCGACGCAACGCGAAGAACCTTACCAAGGCTTGACATACACCGGAAAACCCTGG

AGACAGGGTCCCCCTTGTGGTCGGTGTACAGGTGGTGCATGGCTGTCGTCAGCTCGTGTC

GTGAGATGTTGGGTTAAGTCCCGCAACGAGCGCAACCCTTGTTCTGTGTTGCCAGCATGC

CCTTCGGGGTGATGGGGACTCACAGGAGACTGCCGGGGTCAACTCGGAGGAAGGTGGGGA

CGACGTCAAGTCATCATGCCCCTTATGTCTTGGGCTGCACACGTGCTACAATGGCCGGTA

CAATGAGCTGCGATACCGCGAGGTGGAGCGAATCTCAAAAAGCCGGTCTCAGTTCGGATT

GGGGTCTGCAACTCGACCCCATGAAGTCGGAGTCGCTAGTAATCGCAGATCAGCATTGCT

GCGGTGAATACGTTCCCGGGCCTTGTACACACCGCCCGTCACGTCACGAAAGTCGGTAAC

ACCCGAAGCCGGTGGCCCAACCCCTTGTGGGAGGGAATCGTCGAAGGTGGGACTGGCGAT

TGGGACGAAGTCGTAACAAGGTAGCCGTACCGGAAGGTGCGGCTGGATCACCTCCT

>NR_025621.1 Streptomyces chrestomyceticus JCM 4735 strain DSM 40545 16S ribosomal RNA, partial sequence

AACGCTGGCGGCGTGCTTAACACATGCAAGTCGAACGATGAACCTCCTTCGGGAGGGGAT

TAGTGGCGAACGGGTGAGTAACACGTGGGCAATCTGCCCTGCACTCTGGGACAAGCCCTG

GAAACGGGGTCTAATACCGGATACGACACGGGATCGCATGATCTCCGTGTGGAAAGCTCC

GGCGGTGCAGGATGAGCCCGCGGCCTATCAGCTTGTTGGTGGGGTAATGGCCTACCAAGG

CGACGACGGGTAGCCGGCCTGAGAGGGCGACCGGCCACACTGGGACTGAGACACGGCCCA

GACTCCTACGGGAGGCAGCAGTGGGGAATATTGCACAATGGGCGCAAGCCTGATGCAGCG

ACGCCGCGTGAGGGATGACGGCCTTCGGGTTGTAAACCTCTTTCAGCAGGGAAGAAGCGC

AAGTGACGGTACCTGCAGAAGAAGCGCCGGCTAACTACGTGCCAGCAGCCGCGGTAATAC

GTAGGGCGCAAGCGTTGTCCGGAATTATTGGGCGTAAAGAGCTCGTAGGCGGCTTGTCAC

GTCGGATGTGAAAGCCCGGGGCTTAACCCCGGGTCTGCATTCGATACGGGCAGGCTAGAG

TTCGGTAGGGGAGATCGGAATTCCTGGTGTAGCGGTGAAATGCGCAGATATCAGGAGGAA

CACCGGTGGCGAAGGCGGATCTCTGGGCCGATACTGACGCTGAGGAGCGAAAGCGTGGGG

AGCGAACAGGATTAGATACCCTGGTAGTCCACGCCGTAAACGTTGGGAACTAGGTGTGGG

CGACATTCCACGTCGTCCGTGCCGCAGCTAACGCATTAAGTTCCCCGCCTGGGGAGTACG

GCCGCAAGGCTAAAACTCAAAGGAATTGACGGGGGCCCGCACAAGCGGCGGAGCATGTGG

CTTAATTCGACGCAACGCGAAGAACCTTACCAAGGCTTGACATACACCGGAAAACCCTGG

AGACAGGGTCCCCCTTGTGGTCGGTGTACAGGTGGTGCATGGCTGTCGTCAGCTCGTGTC

GTGAGATGTTGGGTTAAGTCCCGCAACGAGCGCAACCCTTGTTCTGTGTTGCCAGCATGC

CCTTCGGGGTGATGGGGACTCACAGGAGACTGCCGGGGTCAACTCGGAGGAAGGTGGGGA

CGACGTCAAGTCATCATGCCCCTTATGTCTTGGGCTGCACACGTGCTACAATGGCCGGTA

CAATGAGCTGCGATACCGCGAGGTGGAGCGAATCTCAAAAAGCCGGTCTCAGTTCGGATT

GGGGTCTGCAACTCGACCCCATGAAGTCGGAGTCGCTAGTAATCGCAGATCAGCATTGCT

GCGGTGAATACGTTCCCGGGCCTTGTACACACCGCCCGTCACGTCACGAAAGTCGGTAAC

ACCCGAAGCCGGTGGCCCAACCCCTTGTGGGAGGGAATCGTCGAAGGTGGGACTGGCGAT

TGGGACGAAGTCGTAACAAGGTAGCCGTACCGGAAGGTGCGGCTGGATCACCTCCTT

>AJ007399.1 Streptomyces somaliensis strain DSM 40760, 16S rRNA gene

GACGAACGCTGGCGGCGTGCTTAACACATGCAAGTCGAACGATGAACCTCCTTCGGGAGG

GGATTAGTGGCGAACGGGTGAGTAACACGTGGGCAATCTGCCCTGCACTCTGGGACAAGC

CCTGGAAACGGGGTCTAATACCGGATACGACACGGGATCGCATGATCTCCGTGTGGAAAG

CTCCGGCGGTGCAGGATGAGCCCGCGGCCTATCAGCTTGTTGGTGGGGTAATGGCCTACC

AAGGCGACGACGGGTAGCCGGCCTGAGAGGGCGACCGGCCACACTGGGACTGAGACACGG

CCCAGACTCCTACGGGAGGCAGCAGTGGGGAATATTGCACAATGGGCGAAGCCTGATGCA

GCGACGCCGCGTGAGGGATGACGGCCTTCGGGTTGTAAACCTCTTTCAGCAGGGAAGAAG

CGCAAGTGACGGTACCTGCAGAAGAAGCGCCGGCTAACTACGTGCCAGCAGCCGCGGTAA

TACGTAGGGCGCAAGCGTTGTCCGGAATTATTGGGCGTAAAGAGCTCGTAGGCGGCTTGT

CACGTCGGATGTGAAAGCCCGGGGCTTAACCCCGGGTCTGCATTCGATACGGGCAGGCTA

GAGTTCGGTAGGGGAGATCGGAATTCCTGGTGTAGCGGTGAAATGCGCAGATATCAGGAG

GAACACCGGTGGCGAAGGCGGATCTCTGGGCCGATACTGACGCTGAGGAGCGAAAGCGTG

GGGAGCGAACAGGATTAGATACCCTGGTAGTCCACGCCGTAAACGTTGGGAACTAGGTGT

GGGCGACATTCCACGTCGTCCGTGCCGCAGCTAACGCATTAAGTTCCCCGCCTGGGGAGT

ACGGCCGCAAGGCTAAAACTCAAAGGAATTGACGGGGGCCCGCACAAGCGGCGGAGCATG

TGGCTTAATTCGACGCAACGCGAAGAACCTTACCAAGGCTTGACATACACCGGAAAACCC

TGGAGACAGGGTCCCCCTTGTGGTCGGTGTACAGGTGGTGCATGGCTGTCGTCAGCTCGT

GTCGTGAGATGTTGGGTTAAGTCCCGCAACGAGCGCAACCCTTGTTCTGTGTTGCCAGCA

TGCCCTTCGGGGTGATGGGGACTCACAGGAGACTGCCGGGGTCAACTCGGAGGAAGGTGG

GGACGACGTCAAGTCATCATGCCCCTTATGTCTTGGGCTGCACACGTGCTACAATGGCCG

GTACAATGAGCTGCGATACCGCGAGGTGGAGCGAATCTCAAAAAGCCGGTCTCAGTTCGG

ATTGGGGTCTGCAACTCGACCCCATGAAGTCGGAGTCGCTAGTAATCGCAGATCAGCATT

GCTGCGGTGAATACGTTCCCGGGCCTTGTACACACCGCCCGTCACGTCACGAAAGTCGGT

AACACCCGAAGCCGGTGGCCCAACCCCTTGTGGGAGGGAGCTGTCGAAGGTGGGACTGGC

GATTGGGACGAAGTCGTAACAAGGTAGCCGTACCCGAAGGTGC

>KT781120.1 Streptomyces chrestomyceticus strain ADP4 16S ribosomal RNA gene, partial sequence

AACGCTGGCGGCGTGCTTAACACATGCAAGTCGAACGATGAACCTCCTTCGGGAGGGGAT

TAGTGGCGAACGGGTGAGTAACACGTGGGCAATCTGCCCTGCACTCTGGGACAAGCCCTG

GAAACGGGGTCTAATACCGGATACGACACGGGATCGCATGATCTCCGTGTGGAAAGCTCC

GGCGGTGCAGGATGAGCCCGCGGCCTATCAGCTTGTTGGTGGGGTAATGGCCTACCAAGG

CGACGACGGGTAGCCGGCCTGAGAGGGCGACCGGCCACACTGGGACTGAGACACGGCCCA

GACTCCTACGGGAGGCAGCAGTGGGGAATATTGCACAATGGGCGCAAGCCTGATGCAGCG

ACGCCGCGTGAGGGATGACGGCCTTCGGGTTGTAAACCTCTTTCAGCAGGGAAGAAGCGC

AAGTGACGGTACCTGCAGAAGAAGCGCCGGCTAACTACGTGCCAGCAGCCGCGGTAATAC

GTAGGGCGCAAGCGTTGTCCGGAATTATTGGGCGTAAAGAGCTCGTAGGCGGCTTGTCAC

GTCGGATGTGAAAGCCCGGGGCTTAACCCCGGGTCTGCATTCGATACGGGCAGGCTAGAG

TTCGGTAGGGGAGATCGGAATTCCTGGTGTAGCGGTGAAATGCGCAGATATCAGGAGGAA

CACCGGTGGCGAAGGCGGATCTCTGGGCCGATACTGACGCTGAGGAGCGAAAGCGTGGGG

AGCGAACAGGATTAGATACCCTGGTAGTCCACGCCGTAAACGTTGGGAACTAGGTGTGGG

CGACATTCCACGTCGTCCGTGCCGCAGCTAACGCATTAAGTTCCCCGCCTGGGGAGTACG

GCCGCAAGGCTAAAACTCAAAGGAATTGACGGGGGCCCGCACAAGCGGCGGAGCATGTGG

CTTAATTCGACGCAACGCGAAGAACCTTACCAAGGCTTGACATACACCGGAAAACCCTGG

AGACAGGGTCCCCCTTGTGGTCGGTGTACAGGTGGTGCATGGCTGTCGTCAGCTCGTGTC

GTGAGATGTTGGGTTAAGTCCCGCAACGAGCGCAACCCTTGTTCTGTGTTGCCAGCATGC

CCTTCGGGGTGATGGGGACTCACAGGAGACTGCCGGGGTCAACTCGGAGGAAGGTGGGGA

CGACGTCAAGTCATCATGCCCCTTATGTCTTGGGCTGCACACGTGCTACAATGGCCGGTA

CAATGAGCTGCGATACCGCGAGGTGGAGCGAATCTCAAAAAGCCGGTCTCAGTTCGGATT

GGGGTCTGCAACTCGACCCCATGAAGTCGGAGTCGCTAGTAATCGCAGATCAGCATTGCT

GCGGTGAATACGTTCCCGGGCCTTGTACACACCGCCCGTCACGTCACGAAAGTCGGTAAC

ACCCGAAGCCGGTGGCCCAACCCCTTTGTGGGAGGGAATCGTCGAAGGTGGGACTGGCGA

TTGGGACGAAGTCGTAACAAGGTAGCCGTACCGGAAGGTGCGGCTGGATCACCTCCTT

>NR_024760.1 Streptomyces albofaciens JCM 4342 16S ribosomal RNA, partial sequence

GCTCAGGACGAACGCTGGCGGCGTGCTTAACACATGCAAGTCGAACGATGAACCTCCTTC

GGGAGGGGATTAGTGGCGAACGGGTGAGTAACACGTGGGCAATCTGCCCTGCACTCTGGG

ACAAGCCCTGGAAACGGGGTCTAATACCGGATACGACACGGGATCGCATGATCTCCGTGT

GGAAAGCTCCGGCGGTGCAGGATGAGCCCGCGGCCTATCAGCTTGTTGGTGGGGTAATGG

CCTACCAAGGCGACGACGGGTAGCCGGCCTGAGAGGGCGACCGGCCACACTGGGACTGAG

ACACGGCCCAGACTCCTACGGGAGGCAGCAGTGGGGAATATTGCACAATGGGCGCAAGCC

TGATGCAGCGACGCCGCGTGAGGGATGACGGCCTTCGGGTTGTAAACCTCTTTCAGCAGG

GAAGAAGCGCAAGTGACGGTACCTGCAGAAGAAGCGCCGGCTAACTACGTGCCAGCAGCC

GCGGTAATACGTAGGGCGCAAGCGTTGTCCGGAATTATTGGGCGTAAAGAGCTCGTAGGC

GGCTTGTCGCGTCGGATGTGAAAGCCCGGGGCTTAACCCCGGGTCTGCATTCGATACGGG

CAGGCTAGAGTTCGGTAGGGGAGATCGGAATTCCTGGTGTAGCGGTGAAATGCGCAGATA

TCAGGAGGAACACCGGTGGCGAAGGCGGATCTCTGGGCCGATACTGACGCTGAGGAGCGA

AAGCGTGGGGAGCGAACAGGATTAGATACCCTGGTAGTCCACGCCGTAAACGTTGGGAAC

TAGGTGTGGGCGACATTCCACGTCGTCCGTGCCGCAGCTAACGCATTAAGTTCCCCGCCT

GGGGAGTACGGCCGCAAGGCTAAAACTCAAAGGAATTGACGGGGGCCCGCACAAGCGGCG

GAGCATGTGGCTTAATTCGACGCAACGCGAAGAACCTTACCAAGGCTTGACATACACCGG

AAAACCCTGGAGACAGGGTCCCCCTTGTGGTCGGTGTACAGGTGGTGCATGGCTGTCGTC

AGCTCGTGTCGTGAGATGTTGGGTTAAGTCCCGCAACGAGCGCAACCCTTGTTCTGTGTT

GCCAGCACGTCCTTTCGGGGATGGTGGGGACTCACAGGAGACTGCCGGGGTCAACTCGGA

GGAAGGTGGGGACGACGTCAAGTCATCATGCCCCTTATGTCTTGGGCTGCACACGTGCTA

CAATGGCCGGTACAATGAGCTGCGATACCGCGAGGTGGAGCGAATCTCAAAAAGCCGGTC

TCAGTTCGGATTGGGGTCTGCAACTCGACCCCATGAAGTCGGAGTCGCTAGTAATCGCAG

ATCAGCATTGCTGCGGTGAATACGTTCCCGGGCCTTGTACACACCGCCCGTCACGTCACG

AAAGTCGGTAACACCCGAAGCCGGTGGCCCAACCCCTTGTGGGAGGGAATCGTCGAAGGT

GGGACTGGCGATTGGGACGAAGTCGTAACAAGGTAGCCGTACCGGAAGG

>MH021965.1 Streptomyces chrestomyceticus strain S20 16S ribosomal RNA gene, partial sequence

GCTCAGGACGAACGCTGGCGGCGTGCTTAACACATGCAAGTCGAACGATGAACCTCCTTC

GGGAGGGGATTAGTGGCGAACGGGTGAGTAACACGTGGGCAATCTGCCCTGCACTCTGGG

ACAAGCCCTGGAAACGGGGTCTAATACCGGATACGACACGGGATCGCATGGTCTCCGTGT

GGAAAGCTCCGGCGGTGCAGGATGAGCCCGCGGCCTATCAGCTTGTTGGTGGGGTAATGG

CCTACCAAGGCGACGACGGGTAGCCGGCCTGAGAGGGCGACCGGCCACACTGGGACTGAG

ACACGGCCCAGACTCCTACGGGAGGCAGCAGTGGGGAATATTGCACAATGGGCGAAAGCC

TGATGCAGCGACGCCGCGTGAGGGATGACGGCCTTCGGGTTGTAAACCTCTTTCAGCAGG

GAAGAAGCGCAAGTGACGGTACCTGCAGAAGAAGCGCCGGCTAACTACGTGCCAGCAGCC

GCGGTAATACGTAGGGCGCAAGCGTTGTCCGGAATTATTGGGCGTAAAGAGCTCGTAGGC

GGCTTGTCGCGTCGGATGTGAAAGCCCGGGGCTTAACCCCGGGTCTGCATTCGATACGGG

CAGGCTAGAGTTCGGTAGGGGAGATCGGAATTCCTGGTGTAGCGGTGAAATGCGCAGATA

TCAGGAGGAACACCGGTGGCGAAGGCGGATCTCTGGGCCGATACTGACGCTGAGGAGCGA

AAGCGTGGGGAGCGAACAGGATTAGATACCCTGGTAGTCCACGCCGTAAACGTTGGGAAC

TAGGTGTGGGCGACATTCCACGTCGTCCGTGCCGCAGCTAACGCATTAAGTTCCCCGCCT

GGGGAGTACGGCCGCAAGGCTAAAACTCAAAGGAATTGACGGGGGCCCGCACAAGCGGCG

GAGCATGTGGCTTAATTCGACGCAACGCGAAGAACCTTACCAAGGCTTGACATACACCGG

AAACGTCTGGAGACAGGCGCCCCCTTGTGGTCGGTGTACAGGTGGTGCATGGCTGTCGTC

AGCTCGTGTCGTGAGATGTTGGGTTAAGTCCCGCAACGAGCGCAACCCTTGTTCTGTGTT

GCCAGCATGCCCTTCGGGGTGATGGGGACTCACAGGAGACTGCCGGGGTCAACTCGGAGG

AAGGTGGGGACGACGTCAAGTCATCATGCCCCTTATGTCTTGGGCTGCACACGTGCTACA

ATGGCCGGTACAATGAGCTGCGATACCGCGAGGTGGAGCGAATCTCAAAAAGCCGGTCTC

AGTTCGGATTGGGGTCTGCAACTCGACCCCATGAAGTCGGAGTCGCTAGTAATCGCAGAT

CAGCATTGCTGCGGTGAATACGTTCCCGGGCCTTGTACACACCGCCCGTCACGTCACGAA

AGTCGGTAACACCCGAAGCCGGTGGCCCAACCCCTTGTGGGAGGGAATCGTCGAAGGTGG

GACTGGCGATTGGGACGAAGTCGTAACAAGGTA

>JF827350.1 Streptomyces chattanoogensis strain GP4 16S ribosomal RNA gene, partial sequence

AGAGTTTGATCCTGGCTCAGGACGAACGCTGGCGGCGTGCTTAACACATGCAAGTCGAAC

GATGAACCTCCTTCGGGAGGGGATTAGTGGCGAACGGGTGAGTAACACGTGGGCAATCTG

CCCTTCACTCTGGGACAAGCCCTGGAAACGGGGTCTAATACCGGATACGACACGGGGTCG

CATGACCTCCGTGTGGAAAGCTCCGGCGGTGAAGGATGAGCCCGCGGCCTATCAGCTTGT

TGGTGGGGTGATGGCCTACCAAGGCGACGACGGGTAGCCGGCCTGAGAGGGCGACCGGCC

ACACTGGGACTGAGACACGGCCCAGACTCCTACGGGAGGCAGCAGTGGGGAATATTGCAC

AATGGGCGAAAGCCTGATGCAGCGACGCCGCGTGAGGGATGACGGCCTTCGGGTTGTAAA

CCTCTTTCAGCAGGGAAGAAGCGAGAGTGACGGTACCTGCAGAAGAAGCGCCGGCTAACT

ACGTGCCAGCAGCCGCGGTAATACGTAGGGCGCAAGCGTTGTCCGGAATTATTGGGCGTA

AAGAGCTCGTAGGCGGCTTGTCACGTCGGATGTGAAAGCCCGGGGCTTAACCCCGGGTCT

GCATTCGATACGGGCAGGCTAGAGTTCGGTAGGGGAGATCGGAATTCCTGGTGTAGCGGT

GAAATGCGCAGATATCAGGAGGAACACCGGTGGCGAAGGCGGATCTCTGGGCCGATACTG

ACGCTGAGGAGCGAAAGCGTGGGGAGCGAACAGGATTAGATACCCTGGTAGTCCACGCCG

TAAACGTTGGGAACTAGGTGTGGGCGACATTCCACGTCGTCCGTGCCGCAGCTAACGCAT

TAAGTTCCCCGCCTGGGGAGTACGGCCGCAAGGCTAAAACTCAAAGGAATTGACGGGGGC

CCGCACAAGCAGCGGAGCATGTGGCTTAATTCGACGCAACGCGAAGAACCTTACCAAGGC

TTGACATACACCGGAAAACCCTGGAGACAGGGTCCCCCTTGTGGTCGGTGTACAGGTGGT

GCATGGCTGTCGTCAGCTCGTGTCGTGAGATGTTGGGTTAAGTCCCGCAACGAGCGCAAC

CCTTGTTCTGTGTTGCCAGCATGCCCTTCGGGGTGATGGGGACTCACAGGAGACTGCCGG

GGTCAACTCGGAGGAAGGTGGGGACGACGTCAAGTCATCATGCCCCTTATGTCTTGGGCT

GCACACGTGCTACAATGGCCGGTACAATGAGCTGCGATACCGCGAGGTGGAGCGAATCTC

AAAAAGCCGGTCTCAGTTCGGATTGGGGTCTGCAACTCGACCCCATGAAGTCGGAGTCGC

TAGTAATCGCAGATCAGCATTGCTGCGGTGAATACGTTCCCGGGCCTTGTACACACCGCC

CGTCACGTCACGAAAGTCGGTAACACCCGAAGCCGGTGGCCCAACCCCTTGTGGGAGGGA

ATCGTCGAAGGTGGGACTGGCGATTGGGACGAAGTCGTAACAAGGTA

>AB184480.1 Streptomyces lividus gene for 16S rRNA, partial sequence, strain: NBRC 13787

ACGAACGCTGGCGGCGTGCTTAACACATGCAAGTCGAACGATGAACCTCCTTCGGGAGGG

GATTAGTGGCGAACGGGTGAGTAACACGTGGGCAATCTGCCCTGCACTCTGGGACAAGCC

CTGGAAACGGGGTCTAATACCGGATACGACACACGACCGCATGGTCTGTGTGTGGAAAGC

TCCGGCGGTGCAGGATGAGCCCGCGGCCTATCAGCTTGTTGGTGGGGTAATGGCCTACCA

AGGCGACGACGGGTAGCCGGCCTGAGAGGGCGACCGGCCACACTGGGACTGAGACACGGC

CCAGACTCCTACGGGAGGCAGCAGTGGGGAATATTGCACAATGGGCGCAAGCCTGATGCA

GCGACGCCGCGTGAGGGATGACGGCCTTCGGGTTGTAAACCTCTTTCAGCAGGGAAGAAG

CGCAAGTGACGGTACCTGCAGAAGAAGCGCCGGCTAACTACGTGCCAGCAGCCGCGGTAA

TACGTAGGGCGCAAGCGTTGTCCGGAATTATTGGGCGTAAAGAGCTCGTAGGCGGCCTGT

CACGTCGGATGTGAAAGCCCGGGGCTTAACCCCGGGTCTGCATTCGATACGGGCAGGCTA

GAGTTCGGTAGGGGAGATCGGAATTCCTGGTGTAGCGGTGAAATGCGCAGATATCAGGAG

GAACACCGGTGGCGAAGGCGGATCTCTGGGCCGATACTGACGCTGAGGAGCGAAAGCGTG

GGGAGCGAACAGGATTAGATACCCTGGTAGTCCACGCCGTAAACGTTGGGAACTAGGTGT

GGGCGACATTCCACGTCGTCCGTGCCGCAGCTAACGCATTAAGTTCCCCGCCTGGGGAGT

ACGGCCGCAAGGCTAAAACTCAAAGGAATTGACGGGGGCCCGCACAAGCGGCGGAGCATG

TGGCTTAATTCGACGCAACGCGAAGAACCTTACCAAGGCTTGACATACACCGGAAAACCC

TGGAGACAGGGTCCCCCTTGTGGTCGGTGTACAGGTGGTGCATGGCTGTCGTCAGCTCGT

GTCGTGAGATGTTGGGTTAAGTCCCGCAACGAGCGCAACCCTTGTTCTGTGTTGCCAGCA

TGCCCTTCGGGGTGATGGGGACTCACAGGAGACTGCCGGGGTCAACTCGGAGGAAGGTGG

GGACGACGTCAAGTCATCATGCCCCTTATGTCTTGGGCTGCACACGTGCTACAATGGCCG

GTACAATGAGCTGCGATACCGCGAGGTGGAGCGAATCTCAAAAAGCCGGTCTCAGTTCGG

ATTGGGGTCTGCAACTCGACCCCATGAAGTCGGAGTCGCTAGTAATCGCAGATCAGCATT

GCTGCGGTGAATACGTTCCCGGGCCTTGTACACACCGCCCGTCACGTCACGAAAGTCGGT

AACACCCGAAGCCGGTGGCCCAACCCCTTGTGGGAGGGAATCGTCGAAGGTGGGACTGGC

GATTGGGACGAAGTCGTAACAAGGTAGCCGTACCGGAAG

>EU603342.1 Streptomyces sp. MJM3479 16S ribosomal RNA gene, partial sequence

ATGGCGGCGTGCTTACCATGCAAGTCGAACGATGAACCTCCTTCGGGAGGGGATTAGTGG

CGAACGGGTGAGTAACACGTGGGCAATCTGCCCTTCACTCTGGGACAAGCCCTGGAAACG

GGGTCTAATACCGGATACGACACGGGGTCGCATGACCTCCGTGTGGAAAGCTCCGGCGGT

GAAGGATGAGCCCGCGGCCTATCAGCTTGTTGGTGGGGTGATGGCCTACCAAGGCGACGA

CGGGTAGCCGGCCTGAGAGGGCGACCGGCCACACTGGGACTGAGACACGGCCCAGACTCC

TACGGGAGGCAGCAGTGGGGAATATTGCACAATGGGCGAAAGCCTGATGCAGCGACGCCG

CGTGAGGGATGACGGCCTTCGGGTTGTAAACCTCTTTCAGCAGGGAAGAAGCGAGAGTGA

CGGTACCTGCAGAAGAAGCGCCGGCTAACTACGTGCCAGCAGCCGCGGTAATACGTAGGG

CGCAAGCGTTGTCCGGAATTATTGGGCGTAAAGAGCTCGTAGGCGGCTTGTCACGTCGGA

TGTGAAAGCCCGGGGCTTAACCCCGGGTCTGCATTCGATACGGGCAGGCTAGAGTTCGGT

AGGGGAGATCGGAATTCCTGGTGTAGCGGTGAAATGCGCAGATATCAGGAGGAACACCGG

TGGCGAAGGCGGATCTCTGGGCCGATACTGACGCTGAGGAGCGAAAGCGTGGGGAGCGAA

CAGGATTAGATACCCTGGTAGTCCACGCCGTAAACGTTGGGAACTAGGTGTGGGCGACAT

TCCACGTCGTCCGTGCCGCAGCTAACGCATTAAGTTCCCCGCCTGGGGAGTACGGCCGCA

AGGCTAAAACTCAAAGGAATTGACGGGGGCCCGCACAAGCAGCGGAGCATGTGGCTTAAT

TCGACGCAACGCGAAGAACCTTACCAAGGCTTGACATACACCGGAAAACCCTGGAGACAG

GGTCCCCCTTGTGGTCGGTGTACAGGTGGTGCATGGCTGTCGTCAGCTCGTGTCGTGAGA

TGTTGGGTTAAGTCCCGCAACGAGCGCAACCCTTGTTCTGTGTTGCCAGCATGCCCTTCG

GGGTGATGGGGACTCACAGGAGACTGCCGGGGTCAACTCGGAGGAAGGTGGGGACGACGT

CAAGTCATCATGCCCCTTATGTCTTGGGCTGCACACGTGCTACAATGGCCGGTACAATGA

GCTGCGATACCGCGAGGTGGAGCGAATCTCAAAAAGCCGGTCTCAGTTCGGATTGGGGTC

TGCAACTCGACCCCATGAAGTCGGAGTTGCTAGTAATCGCAGATCAGCATTGCTGCGGTG

AATACGTTCCCGGGCCTTGTACACACCGCCCGTCACGTCACGAAAGTCGGTAACACCCGA

AGCCGGTGGCCCAACCCCTTGTGGGAGGGAATCGTCGAAGGTGAC

>NR_112352.1 Streptomyces lydicus strain NBRC 13058 16S ribosomal RNA, partial sequence

GACGAACGCTGGCGGCGTGCTTAACACATGCAAGTCGAACGATGAACCTCCTTCGGGGAG

GGGATTAGTGGCGAACGGGTGAGTAACACGTGGGCAATCTGCCCTTCACTCTGGGACAAG

CCCTGGAAACGGGGTCTAATACCGGATACGACACGGGGTCGCATGACCTCCGTGTGGAAA

GCTCCGGCGGTGAAGGATGAGCCCGCGGCCTATCAGCTTGTTGGTGGGGTGATGGCCTAC

CAAGGCGACGACGGGTAGCCGGCCTGAGAGGGCGACCGGCCACACTGGGACTGAGACACG

GCCCAGACTCCTACGGGAGGCAGCAGTGGGGAATATTGCACAATGGGCGAAAGCCTGATG

CAGCGACGCCGCGTGAGGGATGACGGCCTTCGGGTTGTAAACCTCTTTCAGCAGGGAAGA

AGCGAGAGTGACGGTACCTGCAGAAGAAGCGCCGGCTAACTACGTGCCAGCAGCCGCGGT

AATACGTAGGGCGCAAGCGTTGTCCGGAATTATTGGGCGTAAAGAGCTCGTAGGCGGCTT

GTCACGTCGGATGTGAAAGCCCGGGGCTTAACCCCGGGTCTGCATTCGATACGGGCAGGC

TAGAGTTCGGTAGGGGAGATCGGAATTCCTGGTGTAGCGGTGAAATGCGCAGATATCAGG

AGGAACACCGGTGGCGAAGGCGGATCTCTGGGCCGATACTGACGCTGAGGAGCGAAAGCG

TGGGGAGCGAACAGGATTAGATACCCTGGTAGTCCACGCCGTAAACGTTGGGAACTAGGT

GTGGGCGACATTCCACGTCGTCCGTGCCGCAGCTAACGCATTAAGTTCCCCGCCTGGGGA

GTACGGCCGCAAGGCTAAAACTCAAAGGAATTGACGGGGGCCCGCACAAGCAGCGGAGCA

TGTGGCTTAATTCGACGCAACGCGAAGAACCTTACCAAGGCTTG

>DQ026648.1 Streptomyces flavofuscus strain NRRL B-8036 16S ribosomal RNA gene, partial sequence

ACCTCCTTTCTGAGTTTGATCCTGGCTCAGGACGAACGCTGGCGGCGTGCTTAACACATG

CAAGTCGAACGATGAAGCCGCTTCGGTGGTGGATTAGTGGCGAACGGGTGAGTAACACGT

GGGCAATCTGCCCTGCACTCTGGGACAAGCCCTGGAAACGGGGTCTAATACCGGATACGA

CTGCCTGAGGCATCTCGGGTGGTGGAAAGCTCCGGCGGTGCAGGATGAGCCCGCGGCCTA

TCAGCTTGTTGGTGGGGTAATGGCCTACCAAGGCGACGACGGGTAGCCGGCCTGAGAGGG

CGACCGGCCACACTGGGACTGAGACACGGCCCAGACTCCTACGGGAGGCAGCAGTGGGGA

ATATTGCACAATGGGCGAAAGCCTGATGCAGCGACGCCGCGTGAGGGATGACGGCCTTCG

GGTTGTAAACCTCTTTCAGTAGGGAAGAAGCGCAAGTGACGGTACCTACAGAAGAAGCAC

CGGCTAACTACGTGCCAGCAGCCGCGGTAATACGTAGGGTGCGAGCGTTGTCCGGAATTA

TTGGGCGTAAAGAGCTCGTAGGCGGCTTGTCACGTCGGATGTGAAAGCCCGGGGCTTAAC

CCCGGGTCTGCATTCGATACGGGCAGGCTAGAGTTCGGTAGGGGAGATCGGAATTCCTGG

TGTAGCGGTGAAATGCGCAGATATCAGGAGGAACACCGGTGGCGAAGGCGGATCTCTGGG

CCGATACTGACGCTGAGGAGCGAAAGCGTGGGGAGCGAACAGGATTAGATACCCTGGTAG

TCCACGCCGTAAACGTTGGGAACTAGGTGTGGGCGACATTCCACGTTGTCCGTGCCGCAG

CTAACGCATTAAGTTCCCCGCCTGGGGAGTACGGCCGCAAGGCTAAAACTCAAAGGAATT

GACGGGGGCCCGCACAAGCGGCGGAGCATGTGGCTTAATTCGACGCAACGCGAAGAACCT

TACCAAGGCTTGACATACACCGGAAAACCGTGGAGACACGGTCCCCCTTGTGGTCGGTGT

ACAGGTGGTGCATGGCTGTCGTCAGCTCGTGTCGTGAGATGTTGGGTTAAGTCCCGCAAC

GAGCGCAACCCTTGTTCTGTGTTGCCAGCATGCCTTTCGGGGTGATGGGGACTCACAGGA

GACTGCCGGGGTCAACTCGGAGGAAGGTGGGGACGACGTCAAGTCATCATGCCCCTTATG

TCTTGGGCTGCACACGTGCTACAATGGCCGGTACAATGAGCTGCGATACCGCGAGGTGGA

GCGAATCTCAAAAAGCCGGTCTCAGTTCGGATTGGGGTCTGCAACTCGACCCCATGAAGT

CGGAGTCGCTAGTAATCGCAGATCAGCATTGCTGCGGTGAATACGTTCCCGGGCCTTGTA

CACACCGCCCGTCACGTCACGAAAGTCGGTAACACCCGAAGCCGGTGGCCCAACCCCTTG

TGGGAGGGAATCGTCGAAGGTGGGACTGGCGATTGGGACGAAGTCGTAACAAGGTAGCCG

TACCGGAAGGTGC

>MH430523.2 Streptomyces alkaliterrae strain OF1 16S ribosomal RNA gene, complete sequence

GCATTCATGGAGAGTTTGATCCTGGCTCAGGACGAACGCTGGCGGCGTGCTTAACACATG

CAAGTCGAACGATGAAGCCGCTTCGGTGGTGGATTAGTGGCGAACGGGTGAGTAACACGT

GGGCAATCTGCCCTGCACTCTGGGACAAGCCCTGGAAACGGGGTCTAATACCGGATACGA

CTGCCTGAGGCATCTCGGGTGGTGGAAAGCTCCGGCGGTGCAGGATGAGCCCGCGGCCTA

TCAGCTTGTTGGTGGGGTAATGGCCTACCAAGGCGACGACGGGTAGCCGGCCTGAGAGGG

CGACCGGCCACACTGGGACTGAGACACGGCCCAGACTCCTACGGGAGGCAGCAGTGGGGA

ATATTGCACAATGGGCGAAAGCCTGATGCAGCGACGCCGCGTGAGGGATGACGGCCTTCG

GGTTGTAAACCTCTTTCAGTAGGGAAGAAGCGCAAGTGACGGTACCTACAGAAGAAGCAC

CGGCTAACTACGTGCCAGCAGCCGCGGTAATACGTAGGGTGCGAGCGTTGTCCGGAATTA

TTGGGCGTAAAGAGCTCGTAGGCGGCTTGTCACGTCGGATGTGAAAGCCCGGGGCTTAAC

CCCGGGTCTGCATTCGATACGGGCAGGCTAGAGTTCGGTAGGGGAGATCGGAATTCCTGG

TGTAGCGGTGAAATGCGCAGATATCAGGAGGAACACCGGTGGCGAAGGCGGATCTCTGGG

CCGATACTGACGCTGAGGAGCGAAAGCGTGGGGAGCGAACAGGATTAGATACCCTGGTAG

TCCACGCCGTAAACGTTGGGAACTAGGTGTGGGCGACATTCCACGTTGTCCGTGCCGCAG

CTAACGCATTAAGTTCCCCGCCTGGGGAGTACGGCCGCAAGGCTAAAACTCAAAGGAATT

GACGGGGGCCCGCACAAGCGGCGGAGCATGTGGCTTAATTCGACGCAACGCGAAGAACCT

TACCAAGGCTTGACATACACCGGAAAACCGTGGAGACACGGTCCCCCTTGTGGTCGGTGT

ACAGGTGGTGCATGGCTGTCGTCAGCTCGTGTCGTGAGATGTTGGGTTAAGTCCCGCAAC

GAGCGCAACCCTTGTTCTGTGTTGCCAGCATGCCTTTCGGGGTGATGGGGACTCACAGGA

GACTGCCGGGGTCAACTCGGAGGAAGGTGGGGACGACGTCAAGTCATCATGCCCCTTATG

TCTTGGGCTGCACACGTGCTACAATGGCCGGTACAATGAGCTGCGATACCGTGAGGTGGA

GCGAATCTCAAAAAGCCGGTCTCAGTTCGGATTGGGGTCTGCAACTCGACCCCATGAAGT

CGGAGTCGCTAGTAATCGCAGATCAGCATTGCTGCGGTGAATACGTTCCCGGGCCTTGTA

CACACCGCCCGTCACGTCACGAAAGTCGGTAACACCCGAAGCCGGTGGCCCAACCCCTTG

TGGGAGGGAATCGTCGAAGGTGGGACTGGCGATTGGGACGAAGTCGTAACAAGGTAGCCG

TACCGGAAGGTGCGGCTGGATCACCTCCTTTCTA

>MG198705.1 Streptomyces sp. strain W2233-SM 16S ribosomal RNA gene, partial sequence

TTTTGAGTTTTGGGTTCGGCTCAGGACGAACGCTGGCGGCGTGCTTAACACATGCAAGTC

GAACGATGAAGCCGCTTCGGTGGTGGATTAGTGGCGAACGGGTGAGTAACACGTGGGCAA

TCTGCCCTGCACTCTGGGACAAGCCCTGGAAACGGGGTCTAATACCGGATACGACTGTCT

GAGGCATCTTGGATGGTGGAAAGCTCCGGCGGTGCAGGATGAGCCCGCGGCCTATCAGCT

TGTTGGTGGGGTAATGGCCTACCAAGGCGACGACGGGTAGCCGGCCTGAGAGGGCGACCG

GCCACACTGGGACTGAGACACGGCCCAGACTCCTACGGGAGGCAGCAGTGGGGAATATTG

CACAATGGGCGAAAGCCTGATGCAGCGACGCCGCGTGAGGGATGACGGCCTTCGGGTTGT

AAACCTCTTTCAGTAGGGAAGAAGCGCAAGTGACGGTACCTACAGAAGAAGCACCGGCTA

ACTACGTGCCAGCAGCCGCGGTAATACGTAGGGTGCGAGCGTTGTCCGGAATTATTGGGC

GTAAAGAGCTCGTAGGCGGCTTGTCACGTCGGATGTGAAAGCCCGGGGCTTAACCCCGGG

TCTGCATTCGATACGGGCAGGCTAGAGTTCGGTAGGGGAGATCGGAATTCCTGGTGTAGC

GGTGAAATGCGCAGATATCAGGAGGAACACCGGTGGCGAAGGCGGATCTCTGGGCCGATA

CTGACGCTGAGGAGCGAAAGCGTGGGGAGCGAACAGGATTAGATACCCTGGTAGTCCACG

CCGTAAACGTTGGGAACTAGGTGTGGGCGACATTCCACGTTGTCCGTGCCGCAGCTAACG

CATTAAGTTCCCCGCCTGGGGAGTACGGCCGCAAGGCTAAAACTCAAAGGAATTGACGGG

GGCCCGCACAAGCGGCGGAGCATGTGGCTTAATTCGACGCAACGCGAAGAACCTTACCAA

GGCTTGACATACACCGGAAAACCGTGGAGACACGGTCCCCCTTGTGGTCGGTGTACAGGT

GGTGCATGGCTGTCGTCAGCTCGTGTCGTGAGATGTTGGGTTAAGTCCCGCAACGAGCGC

AACCCTTGTTCTGTGTTGCCAGCATGCCTTTCGGGGTGATGGGGACTCACAGGAGACTGC

CGGGGTCAACTCGGAGGAAGGTGGGGACGACGTCAAGTCATCATGCCCCTTATGTCTTGG

GCTGCACACGTGCTACAATGGCCGGTACAATGAGCTGCGATACCGCGAGGTGGAGCGAAT

CTCAAAAAGCCGGTCTCAGTTCGGATTGGGGTCTGCAACTCGACCCCATGAAGTCGGAGT

CGCTAGTAATCGCAGATCAGCATTGCTGCGGTGAATACGTTCCCGGGCCTTGTACACACC

GCCCGTCACGTCACGAAAGTCGGTAACACCCGAAGCCGGTGGCCAACCCTCGGGACGGAT

GTCGAGTGAGCTT

>KY318506.1 Streptomyces durbertensis strain NEAU-S1GS20 16S ribosomal RNA gene, partial sequence

AGAGTTTGATCCTGGCTCAGGACGAACGCTGGCGGCGTGCTTAACACATGCAAGTCGAAC

GATGAAGCCGCTTCGGTGGTGGATTAGTGGCGAACGGGTGAGTAACACGTGGGCAATCTG

CCCTGCACTCTGGGACAAGCCCTGGAAACGGGGTCTAGTACCGGATACGACCATCTGAGG

CATCTTGGATGGTGGAAAGCTCCGGCGGTGCAGGATGAGCCCGCGGCCTATCAGCTTGTT

GGTGGGGTAATGGCCTACCAAGGCGACGACGGGTAGCCGGCCTGAGAGGGCGACCGGCCA

CACTGGGACTGAGACACGGCCCAGACTCCTACGGGAGGCAGCAGTGGGGAATATTGCACA

ATGGGCGAAAGCCTGATGCAGCGACGCCGCGTGAGGGATGACGGCCTTCGGGTTGTAAAC

CTCTTTCAGTAGGGAAGAAGCGCAAGTGACGGTACCTACAGAAGAAGCACCGGCTAACTA

CGTGCCAGCAGCCGCGGTAATACGTAGGGTGCGAGCGTTGTCCGGAATTATTGGGCGTAA

AGAGCTCGTAGGCGGCTTGTCACGTCGGATGTGAAAGCCCGGGGCTTAACCCCGGGTCTG

CATTCGATACGGGCAGGCTAGAGTTCGGTAGGGGAGATCGGAATTCCTGGTGTAGCGGTG

AAATGCGCAGATATCAGGAGGAACACCGGTGGCGAAGGCGGACCTCTGGGCCGATACTGA

CGCTGAGGAGCGAAAGCGTGGGGAGCGAACAGGATTAGATACCCTGGTAGTCCACGCCGT

AAACGTTGGGAACTAGGTGTGGGCGACATTCCACGTCGTCCGTGCCGCAGCTAACGCATT

AAGTTCCCCGCCTGGGGAGTACGGCCGCAAGGCTAAAACTCAAAGGAATTGACGGGGGCC

CGCACAAGCGGCGGAGCATGTGGCTTAATTCGACGCAACGCGAAGAACCTTACCAAGGCT

TGACATACACCGGAAAACCGTGGAGACACGGTCCCCCTTGTGGTCGGTGTACAGGTGGTG

CATGGCTGTCGTCAGCTCGTGTCGTGAGATGTTGGGTTAAGTCCCGCAACGAGCGCAACC

CTTGTTCTGTGTTGCCAGCATGCCCTTCGGGGTGATGGGGACTCACAGGAGACTGCCGGG

GTCAACTCGGAGGAAGGTGGGGACGACGTCAAGTCATCATGCCCCTTATGTCTTGGGCTG

CACACGTGCTACAATGGCCGGTACAATGAGCTGCGATACCGCGAGGTGGAGCGAATCTCA

AAAAGCCGGTCTCAGTTCGGATTGGGGTCTGCAACTCGACCCCATGAAGTCGGAGTCGCT

AGTAATCGCAGATCAGCATTGCTGCGGTGAATACGTTCCCGGGCCTTGTACACACCGCCC

GTCACGTCACGAAAGTCGGTAACACCCGAAGCCGGTGGCCCAACCCCTTGTGGGAGGGAA

TCGTCGAAGGTGGGACTGGCGATTGGGACGAAGTCGTAACAAGGTAGCCGTACCGGAAGG

TGCGGCTGGATCACCTCCTT

>MT669274.1 Streptomyces durbertensis strain DW15 16S ribosomal RNA gene, partial sequence

CAAGTCGAACGATGAAGCCGCTTCGGTGGTGGATTAGTGGCGAACGGGTGAGTAACACGT

GGGCAATCTGCCCTGCACTCTGGGACAAGCCCTGGAAACGGGGTCTAATACCGGATACGA

CTGCCTGAGGCATCTCGGGTGGTGGAAAGCTCCGGCGGTGCAGGATGAGCCCGCGGCCTA

TCAGCTTGTTGGTGGGGTAATGGCCTACCAAGGCGACGACGGGTAGCCGGCCTGAGAGGG

CGACCGGCCACACTGGGACTGAGACACGGCCCAGACTCCTACGGGAGGCAGCAGTGGGGA

ATATTGCACAATGGGCGAAAGCCTGATGCAGCGACGCCGCGTGAGGGATGACGGCCTTCG

GGTTGTAAACCTCTTTCAGTAGGGAAGAAGCGCAAGTGACGGTACCTACAGAAGAAGCAC

CGGCTAACTACGTGCCAGCAGCCGCGGTAATACGTAGGGTGCGAGCGTTGTCCGGAATTA

TTGGGCGTAAAGAGCTCGTAGGCGGCTTGTCACGTCGGATGTGAAAGCCCGGGGCTTAAC

CCCGGGTCTGCATTCGATACGGGCAGGCTAGAGTTCGGTAGGGGAGATCGGAATTCCTGG

TGTAGCGGTGAAATGCGCAGATATCAGGAGGAACACCGGTGGCGAAGGCGGATCTCTGGG

CCGATACTGACGCTGAGGAGCGAAAGCGTGGGGAGCGAACAGGATTAGATACCCTGGTAG

TCCACGCCGTAAACGTTGGGAACTAGGTGTGGGCGACATTCCACGTTGTCCGTGCCGCAG

CTAACGCATTAAGTTCCCCGCCTGGGGAGTACGGCCGCAAGGCTAAAACTCAAAGGAATT

GACGGGGGCCCGCACAAGCGGCGGAGCATGTGGCTTAATTCGACGCAACGCGAAGAACCT

TACCAAGGCTTGACATACACCGGAAAACCGTGGAGACACGGTCCCCCTTGTGGTCGGTGT

ACAGGTGGTGCATGGCTGTCGTCAGCTCGTGTCGTGAGATGTTGGGTTAAGTCCCGCAAC

GAGCGCAACCCTTGTTCTGTGTTGCCAGCATGCCTTTCGGGGTGATGGGGACTCACAGGA

GACTGCCGGGGTCAACTCGGAGGAAGGTGGGGACGACGTCAAGTCATCATGCCCCTTATG

TCTTGGGCTGCACACGTGCTACAATGGCCGGTACAATGAGCTGCGATACCGCGAGGTGGA

GCGAATCTCAAAAAGCCGGTCTCAGTTCGGATTGGGGTCTGCAACTCGACCCCATGAAGT

CGGAGTCGCTAGTAATCGCAGATCAGCATTGCTGCGGTGAATACGTTCCCGGGCCTTGTA

CACACCGCCCGTCACGTCACGAAAGTCGGTAACACCCGAAGCCGGTGGCCCAACCCCTTG

TGGGAGGGAATCGTCG

>KY523106.1 Streptomyces alkaliterrae strain OF1 16S ribosomal RNA gene, partial sequence

CGAACGATGAAGCCGCTTCGGTGGTGGATTAGTGGCGAACGGGTGAGTAACACGTGGGCA

ATCTGCCCTGCACTCTGGGACAAGCCCTGGAAACGGGGTCTAATACCGGATACGACTGCC

TGAGGCATCTCGGGTGGTGGAAAGCTCCGGCGGTGCAGGATGAGCCCGCGGCCTATCAGC

TTGTTGGTGGGGTAATGGCCTACCAAGGCGACGACGGGTAGCCGGCCTGAGAGGGCGACC

GGCCACACTGGGACTGAGACACGGCCCAGACTCCTACGGGAGGCAGCAGTGGGGAATATT

GCACAATGGGCGAAAGCCTGATGCAGCGACGCCGCGTGAGGGATGACGGCCTTCGGGTTG

TAAACCTCTTTCAGTAGGGAAGAAGCGCAAGTGACGGTACCTACAGAAGAAGCACCGGCT

AACTACGTGCCAGCAGCCGCGGTAATACGTAGGGTGCGAGCGTTGTCCGGAATTATTGGG

CGTAAAGAGCTCGTAGGCGGCTTGTCACGTCGGATGTGAAAGCCCGGGGCTTAACCCCGG

GTCTGCATTCGATACGGGCAGGCTAGAGTTCGGTAGGGGAGATCGGAATTCCTGGTGTAG

CGGTGAAATGCGCAGATATCAGGAGGAACACCGGTGGCGAAGGCGGATCTCTGGGCCGAT

ACTGACGCTGAGGAGCGAAAGCGTGGGGAGCGAACAGGATTAGATACCCTGGTAGTCCAC

GCCGTAAACGTTGGGAACTAGGTGTGGGCGACATTCCACGTTGTCCGTGCCGCAGCTAAC

GCATTAAGTTCCCCGCCTGGGGAGTACGGCCGCAAGGCTAAAACTCAAAGGAATTGACGG

GGGCCCGCACAAGCGGCGGAGCATGTGGCTTAATTCGACGCAACGCGAAGAACCTTACCA

AGGCTTGACATACACCGGAAAACCGTGGAGACACGGTCCCCCTTGTGGTCGGTGTACAGG

TGGTGCATGGCTGTCGTCAGCTCGTGTCGTGAGATGTTGGGTTAAGTCCCGCAACGAGCG

CAACCCTTGTTCTGTGTTGCCAGCATGCCTTTCGGGGTGATGGGGACTCACAGGAGACTG

CCGGGGTCAACTCGGAGGAAGGTGGGGACGACGTCAAGTCATCATGCCCCTTATGTCTTG

GGCTGCACACGTGCTACAATGGCCGGTACAATGAGCTGCGATACCGTGAGGTGGAGCGAA

TCTCAAAAAGCCGGTCTCAGTTCGGATTGGGGTCTGCAACTCGACCCCATGAAGTCGGAG

TCGCTAGTAATCGCAGATCAGCATTGCTGCGGTGAATACGTTCCCGGGCCTTGTACACAC

CGCCCGTCACGTCACGAAAGTCGGTAACACCCGAAGCCGGTGGCCCAACCCCTTGTGGGA

GGGAATCGTCGAAGGTGGGACTGGCGATTGGACG

>MF077006.1 Streptomyces chumphonensis strain 195-LR7 16S ribosomal RNA gene, partial sequence

GTCGAACGATGAAGCCGCTTCGGTGGTGGATTAGTGGCGAACGGGTGCGTAACACGTGGG

CAATCTGCCCTGCACTCTGGGACAAGCCCTGGAAACGGGGTCTAATACCGGATACGACTG

CCTGAGGCATCTCGGGTGGTGGAAAGCTCCGGCGGTGCAGGATGAGCCCGCGGCCTATCA

GCTTGTTGGTGGGGTAATGGCCTACCAAGGCGACGACGGGTAGCCGGCCTGAGAGGGCGA

CCGGCCACACTGGGACTGAGACACGGCCCAGACTCCTACGGGAGGCAGCAGTGGGGAATA

TTGCACAATGGGCGAAAGCCTGATGCAGCGACGCCGCGTGAGGGATGACGGCCTTCGGGT

TGTAAACCTCTTTCAGTAGGGAAGAAGCGCAAGTGACGGTACCTACAGAAGAAGCACCGG

CTAACTACGTGCCAGCAGCCGCGGTAATACGTAGGGTGCGAGCGTTGTCCGGAATTATTG

GGCGTAAAGAGCTCGTAGGCGGCTTGTCACGTCGGATGTGAAAGCCCGGGGCTTAACCCC

GGGTCTGCATTCGATACGGGCAGGCTAGAGTTCGGTAGGGGAGATCGGAATTCCTGGTGT

AGCGGTGAAATGCGCAGATATCAGGAGGAACACCGGTGGCGAAGGCGGATCTCTGGGCCG

ATACTGACGCTGAGGAGCGAAAGCGTGGGGAGCGAACAGGATTAGATACCCTGGTAGTCC

ACGCCGTAAACGTTGGGAACTAGGTGTGGGCGACATTCCACGTTGTCCGTGCCGCAGCTA

ACGCATTAAGTTCCCCGCCTGGGGAGTACGGCCGCAAGGCTAAAACTCAAAGGAATTGAC

GGGGGCCCGCACAAGCGGCGGAGCATGTGGCTTAATTCGACGCAACGCGAAGAACCTTAC

CAAGGCTTGACATACACCGG

CLUSTAL O (1.2.4) multiple sequence alignment

11993_B; ------------------------------------------------------------ 0

11993_A; ----------------------------aacagtgggtcgatgggcgtgcttaacacatg 32

KY318506.1 ----------AGAGTTTGATCCTGGCTCAGGACGAACGCTGGCGGCGTGCTTAACACATG 50

MG198705.1 ------TTTTGAGTTTTGGGTTCGGCTCAGGACGAACGCTGGCGGCGTGCTTAACACATG 54

KY523106.1 ------------------------------------------------------------ 0

MH430523.2 GCATTCATGGAGAGTTTGATCCTGGCTCAGGACGAACGCTGGCGGCGTGCTTAACACATG 60

DQ026648.1 ACCTCCTTTCTGAGTTTGATCCTGGCTCAGGACGAACGCTGGCGGCGTGCTTAACACATG 60

MT669274.1 ------------------------------------------------------------ 0

MF077006.1 ------------------------------------------------------------ 0

EU603342.1 --------------------------------------ATGGCG--GCGTGCTTACCATG 20

JF827350.1 ----------AGAGTTTGATCCTGGCTCAGGACGAACGCTGGCGGCGTGCTTAACACATG 50

NR_112352.1 ------------------------------GACGAACGCTGGCGGCGTGCTTAACACATG 30

NR_024760.1 ------------------------GCTCAGGACGAACGCTGGCGGCGTGCTTAACACATG 36

AJ007399.1 ------------------------------GACGAACGCTGGCGGCGTGCTTAACACATG 30

AB184480.1 -------------------------------ACGAACGCTGGCGGCGTGCTTAACACATG 29

MH021965.1 ------------------------GCTCAGGACGAACGCTGGCGGCGTGCTTAACACATG 36

KT781120.1 ----------------------------------AACGCTGGCGGCGTGCTTAACACATG 26

NR_025622.1 ----------------------------------AACGCTGGCGGCGTGCTTAACACATG 26

NR_025621.1 ----------------------------------AACGCTGGCGGCGTGCTTAACACATG 26

EU741199.1 ---------------------CTGGCTCAGGACGAACGCTGGCGGCGTGCTTAACACATG 39

NR_112400.1 -------------------------------ACGAACGCTGGCGGCGTGCTTAACACATG 29

11993_B; ------------------------------------------------------------ 0

11993_A; caagtcgaacgatgaacctccttcgggagcagggattagtggcgaacgggtgagtaacac 92

KY318506.1 CAAGTCGAACGATGAAGCCGCTTCGG--TGGTGGATTAGTGGCGAACGGGTGAGTAACAC 108

MG198705.1 CAAGTCGAACGATGAAGCCGCTTCGG--TGGTGGATTAGTGGCGAACGGGTGAGTAACAC 112

KY523106.1 -----CGAACGATGAAGCCGCTTCGG--TGGTGGATTAGTGGCGAACGGGTGAGTAACAC 53

MH430523.2 CAAGTCGAACGATGAAGCCGCTTCGG--TGGTGGATTAGTGGCGAACGGGTGAGTAACAC 118

DQ026648.1 CAAGTCGAACGATGAAGCCGCTTCGG--TGGTGGATTAGTGGCGAACGGGTGAGTAACAC 118

MT669274.1 CAAGTCGAACGATGAAGCCGCTTCGG--TGGTGGATTAGTGGCGAACGGGTGAGTAACAC 58

MF077006.1 ---GTCGAACGATGAAGCCGCTTCGG--TGGTGGATTAGTGGCGAACGGGTGCGTAACAC 55

EU603342.1 CAAGTCGAACGATGAACCTCC-TTCGG-GAGGGGATTAGTGGCGAACGGGTGAGTAACAC 78

JF827350.1 CAAGTCGAACGATGAACCTCCTTCGG--GAGGGGATTAGTGGCGAACGGGTGAGTAACAC 108

NR_112352.1 CAAGTCGAACGATGAACCTCCTTCGGG-GAGGGGATTAGTGGCGAACGGGTGAGTAACAC 89

NR_024760.1 CAAGTCGAACGATGAACCTCCTTC-GG-GAGGGGATTAGTGGCGAACGGGTGAGTAACAC 94

AJ007399.1 CAAGTCGAACGATGAACCTCCTTC-GG-GAGGGGATTAGTGGCGAACGGGTGAGTAACAC 88

AB184480.1 CAAGTCGAACGATGAACCTCCTTC-GG-GAGGGGATTAGTGGCGAACGGGTGAGTAACAC 87

MH021965.1 CAAGTCGAACGATGAACCTCCTTC-GG-GAGGGGATTAGTGGCGAACGGGTGAGTAACAC 94

KT781120.1 CAAGTCGAACGATGAACCTCCTTC-GG-GAGGGGATTAGTGGCGAACGGGTGAGTAACAC 84

NR_025622.1 CAAGTCGAACGATGAACCTCCTTC-GG-GAGGGGATTAGTGGCGAACGGGTGAGTAACAC 84

NR_025621.1 CAAGTCGAACGATGAACCTCCTTC-GG-GAGGGGATTAGTGGCGAACGGGTGAGTAACAC 84

EU741199.1 CAAGTCGAACGATGAACCTCCTTC-GG-GAGGGGATTAGTGGCGAACGGGTGAGTAACAC 97

NR_112400.1 CAAGTCGAACGATGAACCTCCTTC-GG-GAGGGGATTAGTGGCGAACGGGTGAGTAACAC 87

11993_B; ------------------------------------------------------------ 0

11993_A; gtgggcaatctgccctgcactctgggacaagcccctggaaacggggtctaataccggata 152

KY318506.1 GTGGGCAATCTGCCCTGCACTCTGGGACAAGCCCTGG-AAACGGGGTCTAGTACCGGATA 167

MG198705.1 GTGGGCAATCTGCCCTGCACTCTGGGACAAGCCCTGG-AAACGGGGTCTAATACCGGATA 171

KY523106.1 GTGGGCAATCTGCCCTGCACTCTGGGACAAGCCCTGG-AAACGGGGTCTAATACCGGATA 112

MH430523.2 GTGGGCAATCTGCCCTGCACTCTGGGACAAGCCCTGG-AAACGGGGTCTAATACCGGATA 177

DQ026648.1 GTGGGCAATCTGCCCTGCACTCTGGGACAAGCCCTGG-AAACGGGGTCTAATACCGGATA 177

MT669274.1 GTGGGCAATCTGCCCTGCACTCTGGGACAAGCCCTGG-AAACGGGGTCTAATACCGGATA 117

MF077006.1 GTGGGCAATCTGCCCTGCACTCTGGGACAAGCCCTGG-AAACGGGGTCTAATACCGGATA 114

EU603342.1 GTGGGCAATCTGCCCTTCACTCTGGGACAAGCCCTGGA-AACGGGGTCTAATACCGGATA 137

JF827350.1 GTGGGCAATCTGCCCTTCACTCTGGGACAAGCCCTGGA-AACGGGGTCTAATACCGGATA 167

NR_112352.1 GTGGGCAATCTGCCCTTCACTCTGGGACAAGCCCTGGA-AACGGGGTCTAATACCGGATA 148

NR_024760.1 GTGGGCAATCTGCCCTGCACTCTGGGACAAGCCCTGGA-AACGGGGTCTAATACCGGATA 153

AJ007399.1 GTGGGCAATCTGCCCTGCACTCTGGGACAAGCCCTGGA-AACGGGGTCTAATACCGGATA 147

AB184480.1 GTGGGCAATCTGCCCTGCACTCTGGGACAAGCCCTGGA-AACGGGGTCTAATACCGGATA 146

MH021965.1 GTGGGCAATCTGCCCTGCACTCTGGGACAAGCCCTGGA-AACGGGGTCTAATACCGGATA 153

KT781120.1 GTGGGCAATCTGCCCTGCACTCTGGGACAAGCCCTGGA-AACGGGGTCTAATACCGGATA 143

NR_025622.1 GTGGGCAATCTGCCCTGCACTCTGGGACAAGCCCTGGA-AACGGGGTCTAATACCGGATA 143

NR_025621.1 GTGGGCAATCTGCCCTGCACTCTGGGACAAGCCCTGGA-AACGGGGTCTAATACCGGATA 143

EU741199.1 GTGGGCAATCTGCCCTGCACTCTGGGACAAGCCCTGGA-AACGGGGTCTAATACCGGATA 156

NR_112400.1 GTGGGCAATCTGCCCTGCACTCTGGGACAAGCCCTGGA-AACGGGGTCTAATACCGGATA 146

11993_B; ------------------------------------------------------------ 0

11993_A; cgacacgggatcgcatgatctccgtgtggaaagctccggcggtgcaggatgagcccgcgg 212

KY318506.1 CGACCATCTGAGG-CATCTTGGATGGTGGAAAGCTCCGGCGGTGCAGGATGAGCCCGCGG 226

MG198705.1 CGACTGTCTGAGG-CATCTTGGATGGTGGAAAGCTCCGGCGGTGCAGGATGAGCCCGCGG 230

KY523106.1 CGACTGCCTGAGG-CATCTCGGGTGGTGGAAAGCTCCGGCGGTGCAGGATGAGCCCGCGG 171

MH430523.2 CGACTGCCTGAGG-CATCTCGGGTGGTGGAAAGCTCCGGCGGTGCAGGATGAGCCCGCGG 236

DQ026648.1 CGACTGCCTGAGG-CATCTCGGGTGGTGGAAAGCTCCGGCGGTGCAGGATGAGCCCGCGG 236

MT669274.1 CGACTGCCTGAGG-CATCTCGGGTGGTGGAAAGCTCCGGCGGTGCAGGATGAGCCCGCGG 176

MF077006.1 CGACTGCCTGAGG-CATCTCGGGTGGTGGAAAGCTCCGGCGGTGCAGGATGAGCCCGCGG 173

EU603342.1 CGACACGGGGTCGCATGACCTCCGTGTGGAAAGCTCCGGCGGTGAAGGATGAGCCCGCGG 197

JF827350.1 CGACACGGGGTCGCATGACCTCCGTGTGGAAAGCTCCGGCGGTGAAGGATGAGCCCGCGG 227

NR_112352.1 CGACACGGGGTCGCATGACCTCCGTGTGGAAAGCTCCGGCGGTGAAGGATGAGCCCGCGG 208

NR_024760.1 CGACACGGGATCGCATGATCTCCGTGTGGAAAGCTCCGGCGGTGCAGGATGAGCCCGCGG 213

AJ007399.1 CGACACGGGATCGCATGATCTCCGTGTGGAAAGCTCCGGCGGTGCAGGATGAGCCCGCGG 207

AB184480.1 CGACACACGACCGCATGGTCTGTGTGTGGAAAGCTCCGGCGGTGCAGGATGAGCCCGCGG 206

MH021965.1 CGACACGGGATCGCATGGTCTCCGTGTGGAAAGCTCCGGCGGTGCAGGATGAGCCCGCGG 213

KT781120.1 CGACACGGGATCGCATGATCTCCGTGTGGAAAGCTCCGGCGGTGCAGGATGAGCCCGCGG 203

NR_025622.1 CGACACGGGATCGCATGATCTCCGTGTGGAAAGCTCCGGCGGTGCAGGATGAGCCCGCGG 203

NR_025621.1 CGACACGGGATCGCATGATCTCCGTGTGGAAAGCTCCGGCGGTGCAGGATGAGCCCGCGG 203

EU741199.1 CGACACGGGATCGCATGATCTCCGTGTGGAAAGCTCCGGCGGTGCAGGATGAGCCCGCGG 216

NR_112400.1 CGACACGGGATCGCATGATCTCCGTGTGGAAAGCTCCGGCGGTGCAGGATGAGCCCGCGG 206

11993_B; ------------------------------------------------------------ 0

11993_A; cctatcagcttgttggtggggtaatggcctaccacaaggcgacgacgggtagccggcctg 272

KY318506.1 CCTATCAGCTTGTTGGTGGGGTAATGGCCTACCA--AGGCGACGACGGGTAGCCGGCCTG 284

MG198705.1 CCTATCAGCTTGTTGGTGGGGTAATGGCCTACCA--AGGCGACGACGGGTAGCCGGCCTG 288

KY523106.1 CCTATCAGCTTGTTGGTGGGGTAATGGCCTACCA--AGGCGACGACGGGTAGCCGGCCTG 229

MH430523.2 CCTATCAGCTTGTTGGTGGGGTAATGGCCTACCA--AGGCGACGACGGGTAGCCGGCCTG 294

DQ026648.1 CCTATCAGCTTGTTGGTGGGGTAATGGCCTACCA--AGGCGACGACGGGTAGCCGGCCTG 294

MT669274.1 CCTATCAGCTTGTTGGTGGGGTAATGGCCTACCA--AGGCGACGACGGGTAGCCGGCCTG 234

MF077006.1 CCTATCAGCTTGTTGGTGGGGTAATGGCCTACCA--AGGCGACGACGGGTAGCCGGCCTG 231

EU603342.1 CCTATCAGCTTGTTGGTGGGGTGATGGCCTACCA--AGGCGACGACGGGTAGCCGGCCTG 255

JF827350.1 CCTATCAGCTTGTTGGTGGGGTGATGGCCTACCA--AGGCGACGACGGGTAGCCGGCCTG 285

NR_112352.1 CCTATCAGCTTGTTGGTGGGGTGATGGCCTACCA--AGGCGACGACGGGTAGCCGGCCTG 266

NR_024760.1 CCTATCAGCTTGTTGGTGGGGTAATGGCCTACCA--AGGCGACGACGGGTAGCCGGCCTG 271

AJ007399.1 CCTATCAGCTTGTTGGTGGGGTAATGGCCTACCA--AGGCGACGACGGGTAGCCGGCCTG 265

AB184480.1 CCTATCAGCTTGTTGGTGGGGTAATGGCCTACCA--AGGCGACGACGGGTAGCCGGCCTG 264

MH021965.1 CCTATCAGCTTGTTGGTGGGGTAATGGCCTACCA--AGGCGACGACGGGTAGCCGGCCTG 271

KT781120.1 CCTATCAGCTTGTTGGTGGGGTAATGGCCTACCA--AGGCGACGACGGGTAGCCGGCCTG 261

NR_025622.1 CCTATCAGCTTGTTGGTGGGGTAATGGCCTACCA--AGGCGACGACGGGTAGCCGGCCTG 261

NR_025621.1 CCTATCAGCTTGTTGGTGGGGTAATGGCCTACCA--AGGCGACGACGGGTAGCCGGCCTG 261

EU741199.1 CCTATCAGCTTGTTGGTGGGGTAATGGCCTACCA--AGGCGACGACGGGTAGCCGGCCTG 274

NR_112400.1 CCTATCAGCTTGTTGGTGGGGTAATGGCCTACCA--AGGCGACGACGGGTAGCCGGCCTG 264

11993_B; ------------------------------------------------------------ 0

11993_A; agagggcgaccggccacactgggactgagacacggcccagactcctacgggaggcagcag 332

KY318506.1 AGAGGGCGACCGGCCACACTGGGACTGAGACACGGCCCAGACTCCTACGGGAGGCAGCAG 344

MG198705.1 AGAGGGCGACCGGCCACACTGGGACTGAGACACGGCCCAGACTCCTACGGGAGGCAGCAG 348

KY523106.1 AGAGGGCGACCGGCCACACTGGGACTGAGACACGGCCCAGACTCCTACGGGAGGCAGCAG 289

MH430523.2 AGAGGGCGACCGGCCACACTGGGACTGAGACACGGCCCAGACTCCTACGGGAGGCAGCAG 354

DQ026648.1 AGAGGGCGACCGGCCACACTGGGACTGAGACACGGCCCAGACTCCTACGGGAGGCAGCAG 354

MT669274.1 AGAGGGCGACCGGCCACACTGGGACTGAGACACGGCCCAGACTCCTACGGGAGGCAGCAG 294

MF077006.1 AGAGGGCGACCGGCCACACTGGGACTGAGACACGGCCCAGACTCCTACGGGAGGCAGCAG 291

EU603342.1 AGAGGGCGACCGGCCACACTGGGACTGAGACACGGCCCAGACTCCTACGGGAGGCAGCAG 315

JF827350.1 AGAGGGCGACCGGCCACACTGGGACTGAGACACGGCCCAGACTCCTACGGGAGGCAGCAG 345

NR_112352.1 AGAGGGCGACCGGCCACACTGGGACTGAGACACGGCCCAGACTCCTACGGGAGGCAGCAG 326

NR_024760.1 AGAGGGCGACCGGCCACACTGGGACTGAGACACGGCCCAGACTCCTACGGGAGGCAGCAG 331

AJ007399.1 AGAGGGCGACCGGCCACACTGGGACTGAGACACGGCCCAGACTCCTACGGGAGGCAGCAG 325

AB184480.1 AGAGGGCGACCGGCCACACTGGGACTGAGACACGGCCCAGACTCCTACGGGAGGCAGCAG 324

MH021965.1 AGAGGGCGACCGGCCACACTGGGACTGAGACACGGCCCAGACTCCTACGGGAGGCAGCAG 331

KT781120.1 AGAGGGCGACCGGCCACACTGGGACTGAGACACGGCCCAGACTCCTACGGGAGGCAGCAG 321

NR_025622.1 AGAGGGCGACCGGCCACACTGGGACTGAGACACGGCCCAGACTCCTACGGGAGGCAGCAG 321

NR_025621.1 AGAGGGCGACCGGCCACACTGGGACTGAGACACGGCCCAGACTCCTACGGGAGGCAGCAG 321

EU741199.1 AGAGGGCGACCGGCCACACTGGGACTGAGACACGGCCCAGACTCCTACGGGAGGCAGCAG 334

NR_112400.1 AGAGGGCGACCGGCCACACTGGGACTGAGACACGGCCCAGACTCCTACGGGAGGCAGCAG 324

11993_B; ------------------------------------------------------------ 0

11993_A; tggggaatattgcacaatgggcgaaagcctgatgcagcgacgccgcgtgagggatgacgg 392

KY318506.1 TGGGGAATATTGCACAATGGGCGAAAGCCTGATGCAGCGACGCCGCGTGAGGGATGACGG 404

MG198705.1 TGGGGAATATTGCACAATGGGCGAAAGCCTGATGCAGCGACGCCGCGTGAGGGATGACGG 408

KY523106.1 TGGGGAATATTGCACAATGGGCGAAAGCCTGATGCAGCGACGCCGCGTGAGGGATGACGG 349

MH430523.2 TGGGGAATATTGCACAATGGGCGAAAGCCTGATGCAGCGACGCCGCGTGAGGGATGACGG 414

DQ026648.1 TGGGGAATATTGCACAATGGGCGAAAGCCTGATGCAGCGACGCCGCGTGAGGGATGACGG 414

MT669274.1 TGGGGAATATTGCACAATGGGCGAAAGCCTGATGCAGCGACGCCGCGTGAGGGATGACGG 354

MF077006.1 TGGGGAATATTGCACAATGGGCGAAAGCCTGATGCAGCGACGCCGCGTGAGGGATGACGG 351

EU603342.1 TGGGGAATATTGCACAATGGGCGAAAGCCTGATGCAGCGACGCCGCGTGAGGGATGACGG 375

JF827350.1 TGGGGAATATTGCACAATGGGCGAAAGCCTGATGCAGCGACGCCGCGTGAGGGATGACGG 405

NR_112352.1 TGGGGAATATTGCACAATGGGCGAAAGCCTGATGCAGCGACGCCGCGTGAGGGATGACGG 386

NR_024760.1 TGGGGAATATTGCACAATGGGCGCAAGCCTGATGCAGCGACGCCGCGTGAGGGATGACGG 391

AJ007399.1 TGGGGAATATTGCACAATGGGCG-AAGCCTGATGCAGCGACGCCGCGTGAGGGATGACGG 384

AB184480.1 TGGGGAATATTGCACAATGGGCGCAAGCCTGATGCAGCGACGCCGCGTGAGGGATGACGG 384

MH021965.1 TGGGGAATATTGCACAATGGGCGAAAGCCTGATGCAGCGACGCCGCGTGAGGGATGACGG 391

KT781120.1 TGGGGAATATTGCACAATGGGCGCAAGCCTGATGCAGCGACGCCGCGTGAGGGATGACGG 381

NR_025622.1 TGGGGAATATTGCACAATGGGCGCAAGCCTGATGCAGCGACGCCGCGTGAGGGATGACGG 381

NR_025621.1 TGGGGAATATTGCACAATGGGCGCAAGCCTGATGCAGCGACGCCGCGTGAGGGATGACGG 381

EU741199.1 TGGGGAATATTGCACAATGGGCGAAAGCCTGATGCAGCGACGCCGCGTGAGGGATGACGG 394

NR_112400.1 TGGGGAATATTGCACAATGGGCGNAAGCCTGATGCAGCGACGCCGCGTGAGGGATGACGG 384

11993_B; ------------------------------------------------------------ 0

11993_A; ccttcgggttgtaaacctctttcagcagggaagaagcgcaagtgacggtacctgcagaag 452

KY318506.1 CCTTCGGGTTGTAAACCTCTTTCAGTAGGGAAGAAGCGCAAGTGACGGTACCTACAGAAG 464

MG198705.1 CCTTCGGGTTGTAAACCTCTTTCAGTAGGGAAGAAGCGCAAGTGACGGTACCTACAGAAG 468

KY523106.1 CCTTCGGGTTGTAAACCTCTTTCAGTAGGGAAGAAGCGCAAGTGACGGTACCTACAGAAG 409

MH430523.2 CCTTCGGGTTGTAAACCTCTTTCAGTAGGGAAGAAGCGCAAGTGACGGTACCTACAGAAG 474

DQ026648.1 CCTTCGGGTTGTAAACCTCTTTCAGTAGGGAAGAAGCGCAAGTGACGGTACCTACAGAAG 474

MT669274.1 CCTTCGGGTTGTAAACCTCTTTCAGTAGGGAAGAAGCGCAAGTGACGGTACCTACAGAAG 414

MF077006.1 CCTTCGGGTTGTAAACCTCTTTCAGTAGGGAAGAAGCGCAAGTGACGGTACCTACAGAAG 411

EU603342.1 CCTTCGGGTTGTAAACCTCTTTCAGCAGGGAAGAAGCGAGAGTGACGGTACCTGCAGAAG 435

JF827350.1 CCTTCGGGTTGTAAACCTCTTTCAGCAGGGAAGAAGCGAGAGTGACGGTACCTGCAGAAG 465

NR_112352.1 CCTTCGGGTTGTAAACCTCTTTCAGCAGGGAAGAAGCGAGAGTGACGGTACCTGCAGAAG 446

NR_024760.1 CCTTCGGGTTGTAAACCTCTTTCAGCAGGGAAGAAGCGCAAGTGACGGTACCTGCAGAAG 451

AJ007399.1 CCTTCGGGTTGTAAACCTCTTTCAGCAGGGAAGAAGCGCAAGTGACGGTACCTGCAGAAG 444

AB184480.1 CCTTCGGGTTGTAAACCTCTTTCAGCAGGGAAGAAGCGCAAGTGACGGTACCTGCAGAAG 444

MH021965.1 CCTTCGGGTTGTAAACCTCTTTCAGCAGGGAAGAAGCGCAAGTGACGGTACCTGCAGAAG 451

KT781120.1 CCTTCGGGTTGTAAACCTCTTTCAGCAGGGAAGAAGCGCAAGTGACGGTACCTGCAGAAG 441

NR_025622.1 CCTTCGGGTTGTAAACCTCTTTCAGCAGGGAAGAAGCGCAAGTGACGGTACCTGCAGAAG 441

NR_025621.1 CCTTCGGGTTGTAAACCTCTTTCAGCAGGGAAGAAGCGCAAGTGACGGTACCTGCAGAAG 441

EU741199.1 CCTTCGGGTTGTAAACCTCTTTCAGCAGGGAAGAAGCGCAAGTGACGGTACCTGCAGAAG 454

NR_112400.1 CCTTCGGGTTGTAAACCTCTTTCAGCAGGGAAGAAGCGCAAGTGACGGTACCTGCAGAAG 444

11993_B; ------------------------------------------------------------ 0

11993_A; aagcgccggctaactacgtgccagcagccgcggtaatacgtagggcgcaagcgttgtccg 512

KY318506.1 AAGCACCGGCTAACTACGTGCCAGCAGCCGCGGTAATACGTAGGGTGCGAGCGTTGTCCG 524

MG198705.1 AAGCACCGGCTAACTACGTGCCAGCAGCCGCGGTAATACGTAGGGTGCGAGCGTTGTCCG 528

KY523106.1 AAGCACCGGCTAACTACGTGCCAGCAGCCGCGGTAATACGTAGGGTGCGAGCGTTGTCCG 469

MH430523.2 AAGCACCGGCTAACTACGTGCCAGCAGCCGCGGTAATACGTAGGGTGCGAGCGTTGTCCG 534

DQ026648.1 AAGCACCGGCTAACTACGTGCCAGCAGCCGCGGTAATACGTAGGGTGCGAGCGTTGTCCG 534

MT669274.1 AAGCACCGGCTAACTACGTGCCAGCAGCCGCGGTAATACGTAGGGTGCGAGCGTTGTCCG 474

MF077006.1 AAGCACCGGCTAACTACGTGCCAGCAGCCGCGGTAATACGTAGGGTGCGAGCGTTGTCCG 471

EU603342.1 AAGCGCCGGCTAACTACGTGCCAGCAGCCGCGGTAATACGTAGGGCGCAAGCGTTGTCCG 495

JF827350.1 AAGCGCCGGCTAACTACGTGCCAGCAGCCGCGGTAATACGTAGGGCGCAAGCGTTGTCCG 525

NR_112352.1 AAGCGCCGGCTAACTACGTGCCAGCAGCCGCGGTAATACGTAGGGCGCAAGCGTTGTCCG 506

NR_024760.1 AAGCGCCGGCTAACTACGTGCCAGCAGCCGCGGTAATACGTAGGGCGCAAGCGTTGTCCG 511

AJ007399.1 AAGCGCCGGCTAACTACGTGCCAGCAGCCGCGGTAATACGTAGGGCGCAAGCGTTGTCCG 504

AB184480.1 AAGCGCCGGCTAACTACGTGCCAGCAGCCGCGGTAATACGTAGGGCGCAAGCGTTGTCCG 504

MH021965.1 AAGCGCCGGCTAACTACGTGCCAGCAGCCGCGGTAATACGTAGGGCGCAAGCGTTGTCCG 511

KT781120.1 AAGCGCCGGCTAACTACGTGCCAGCAGCCGCGGTAATACGTAGGGCGCAAGCGTTGTCCG 501

NR_025622.1 AAGCGCCGGCTAACTACGTGCCAGCAGCCGCGGTAATACGTAGGGCGCAAGCGTTGTCCG 501

NR_025621.1 AAGCGCCGGCTAACTACGTGCCAGCAGCCGCGGTAATACGTAGGGCGCAAGCGTTGTCCG 501

EU741199.1 AAGCGCCGGCTAACTACGTGCCAGCAGCCGCGGTAATACGTAGGGCGCAAGCGTTGTCCG 514

NR_112400.1 AAGCGCCGGCTAACTACGTGCCAGCAGCCGCGGTAATACGTAGGGCGCAAGCGTTGTCCG 504

11993_B; ------------------------------------------------------------ 0

11993_A; gaattattgggcgtaaagagctcgtaggcggcttgtcacgtcggatgtgaaagcccgggg 572

KY318506.1 GAATTATTGGGCGTAAAGAGCTCGTAGGCGGCTTGTCACGTCGGATGTGAAAGCCCGGGG 584

MG198705.1 GAATTATTGGGCGTAAAGAGCTCGTAGGCGGCTTGTCACGTCGGATGTGAAAGCCCGGGG 588

KY523106.1 GAATTATTGGGCGTAAAGAGCTCGTAGGCGGCTTGTCACGTCGGATGTGAAAGCCCGGGG 529

MH430523.2 GAATTATTGGGCGTAAAGAGCTCGTAGGCGGCTTGTCACGTCGGATGTGAAAGCCCGGGG 594

DQ026648.1 GAATTATTGGGCGTAAAGAGCTCGTAGGCGGCTTGTCACGTCGGATGTGAAAGCCCGGGG 594

MT669274.1 GAATTATTGGGCGTAAAGAGCTCGTAGGCGGCTTGTCACGTCGGATGTGAAAGCCCGGGG 534

MF077006.1 GAATTATTGGGCGTAAAGAGCTCGTAGGCGGCTTGTCACGTCGGATGTGAAAGCCCGGGG 531

EU603342.1 GAATTATTGGGCGTAAAGAGCTCGTAGGCGGCTTGTCACGTCGGATGTGAAAGCCCGGGG 555

JF827350.1 GAATTATTGGGCGTAAAGAGCTCGTAGGCGGCTTGTCACGTCGGATGTGAAAGCCCGGGG 585

NR_112352.1 GAATTATTGGGCGTAAAGAGCTCGTAGGCGGCTTGTCACGTCGGATGTGAAAGCCCGGGG 566

NR_024760.1 GAATTATTGGGCGTAAAGAGCTCGTAGGCGGCTTGTCGCGTCGGATGTGAAAGCCCGGGG 571

AJ007399.1 GAATTATTGGGCGTAAAGAGCTCGTAGGCGGCTTGTCACGTCGGATGTGAAAGCCCGGGG 564

AB184480.1 GAATTATTGGGCGTAAAGAGCTCGTAGGCGGCCTGTCACGTCGGATGTGAAAGCCCGGGG 564

MH021965.1 GAATTATTGGGCGTAAAGAGCTCGTAGGCGGCTTGTCGCGTCGGATGTGAAAGCCCGGGG 571

KT781120.1 GAATTATTGGGCGTAAAGAGCTCGTAGGCGGCTTGTCACGTCGGATGTGAAAGCCCGGGG 561

NR_025622.1 GAATTATTGGGCGTAAAGAGCTCGTAGGCGGCTTGTCACGTCGGATGTGAAAGCCCGGGG 561

NR_025621.1 GAATTATTGGGCGTAAAGAGCTCGTAGGCGGCTTGTCACGTCGGATGTGAAAGCCCGGGG 561

EU741199.1 GAATTATTGGGCGTAAAGAGCTCGTAGGCGGCTTGTCACGTCGGATGTGAAAGCCCGGGG 574

NR_112400.1 GAATTATTGGGCGTAAAGAGCTCGTAGGCGGCTTGTCACGTCGGATGTGAAAGCCCGGGG 564

11993_B; ------------------------------------------------------------ 0

11993_A; cttaaccccgggtctgcattcgatacgggcaggctagagttcggtaggggagatcggaat 632

KY318506.1 CTTAACCCCGGGTCTGCATTCGATACGGGCAGGCTAGAGTTCGGTAGGGGAGATCGGAAT 644

MG198705.1 CTTAACCCCGGGTCTGCATTCGATACGGGCAGGCTAGAGTTCGGTAGGGGAGATCGGAAT 648

KY523106.1 CTTAACCCCGGGTCTGCATTCGATACGGGCAGGCTAGAGTTCGGTAGGGGAGATCGGAAT 589

MH430523.2 CTTAACCCCGGGTCTGCATTCGATACGGGCAGGCTAGAGTTCGGTAGGGGAGATCGGAAT 654

DQ026648.1 CTTAACCCCGGGTCTGCATTCGATACGGGCAGGCTAGAGTTCGGTAGGGGAGATCGGAAT 654

MT669274.1 CTTAACCCCGGGTCTGCATTCGATACGGGCAGGCTAGAGTTCGGTAGGGGAGATCGGAAT 594

MF077006.1 CTTAACCCCGGGTCTGCATTCGATACGGGCAGGCTAGAGTTCGGTAGGGGAGATCGGAAT 591

EU603342.1 CTTAACCCCGGGTCTGCATTCGATACGGGCAGGCTAGAGTTCGGTAGGGGAGATCGGAAT 615

JF827350.1 CTTAACCCCGGGTCTGCATTCGATACGGGCAGGCTAGAGTTCGGTAGGGGAGATCGGAAT 645

NR_112352.1 CTTAACCCCGGGTCTGCATTCGATACGGGCAGGCTAGAGTTCGGTAGGGGAGATCGGAAT 626

NR_024760.1 CTTAACCCCGGGTCTGCATTCGATACGGGCAGGCTAGAGTTCGGTAGGGGAGATCGGAAT 631

AJ007399.1 CTTAACCCCGGGTCTGCATTCGATACGGGCAGGCTAGAGTTCGGTAGGGGAGATCGGAAT 624

AB184480.1 CTTAACCCCGGGTCTGCATTCGATACGGGCAGGCTAGAGTTCGGTAGGGGAGATCGGAAT 624

MH021965.1 CTTAACCCCGGGTCTGCATTCGATACGGGCAGGCTAGAGTTCGGTAGGGGAGATCGGAAT 631

KT781120.1 CTTAACCCCGGGTCTGCATTCGATACGGGCAGGCTAGAGTTCGGTAGGGGAGATCGGAAT 621

NR_025622.1 CTTAACCCCGGGTCTGCATTCGATACGGGCAGGCTAGAGTTCGGTAGGGGAGATCGGAAT 621

NR_025621.1 CTTAACCCCGGGTCTGCATTCGATACGGGCAGGCTAGAGTTCGGTAGGGGAGATCGGAAT 621

EU741199.1 CTTAACCCCGGGTCTGCATTCGATACGGGCAGGCTAGAGTTCGGTAGGGGAGATCGGAAT 634

NR_112400.1 CTTAACCCCGGGTCTGCATTCGATACGGGCAGGCTAGAGTTCGGTAGGGGAGATCGGAAT 624

11993_B; ------------------------------------------------------------ 0

11993_A; tcctggtgtagcggtgaaatgcgcagatatcaggaggaacaccggtggcgaaggcggatc 692

KY318506.1 TCCTGGTGTAGCGGTGAAATGCGCAGATATCAGGAGGAACACCGGTGGCGAAGGCGGACC 704

MG198705.1 TCCTGGTGTAGCGGTGAAATGCGCAGATATCAGGAGGAACACCGGTGGCGAAGGCGGATC 708

KY523106.1 TCCTGGTGTAGCGGTGAAATGCGCAGATATCAGGAGGAACACCGGTGGCGAAGGCGGATC 649

MH430523.2 TCCTGGTGTAGCGGTGAAATGCGCAGATATCAGGAGGAACACCGGTGGCGAAGGCGGATC 714

DQ026648.1 TCCTGGTGTAGCGGTGAAATGCGCAGATATCAGGAGGAACACCGGTGGCGAAGGCGGATC 714

MT669274.1 TCCTGGTGTAGCGGTGAAATGCGCAGATATCAGGAGGAACACCGGTGGCGAAGGCGGATC 654

MF077006.1 TCCTGGTGTAGCGGTGAAATGCGCAGATATCAGGAGGAACACCGGTGGCGAAGGCGGATC 651

EU603342.1 TCCTGGTGTAGCGGTGAAATGCGCAGATATCAGGAGGAACACCGGTGGCGAAGGCGGATC 675

JF827350.1 TCCTGGTGTAGCGGTGAAATGCGCAGATATCAGGAGGAACACCGGTGGCGAAGGCGGATC 705

NR_112352.1 TCCTGGTGTAGCGGTGAAATGCGCAGATATCAGGAGGAACACCGGTGGCGAAGGCGGATC 686

NR_024760.1 TCCTGGTGTAGCGGTGAAATGCGCAGATATCAGGAGGAACACCGGTGGCGAAGGCGGATC 691

AJ007399.1 TCCTGGTGTAGCGGTGAAATGCGCAGATATCAGGAGGAACACCGGTGGCGAAGGCGGATC 684

AB184480.1 TCCTGGTGTAGCGGTGAAATGCGCAGATATCAGGAGGAACACCGGTGGCGAAGGCGGATC 684

MH021965.1 TCCTGGTGTAGCGGTGAAATGCGCAGATATCAGGAGGAACACCGGTGGCGAAGGCGGATC 691

KT781120.1 TCCTGGTGTAGCGGTGAAATGCGCAGATATCAGGAGGAACACCGGTGGCGAAGGCGGATC 681

NR_025622.1 TCCTGGTGTAGCGGTGAAATGCGCAGATATCAGGAGGAACACCGGTGGCGAAGGCGGATC 681

NR_025621.1 TCCTGGTGTAGCGGTGAAATGCGCAGATATCAGGAGGAACACCGGTGGCGAAGGCGGATC 681

EU741199.1 TCCTGGTGTAGCGGTGAAATGCGCAGATATCAGGAGGAACACCGGTGGCGAAGGCGGATC 694

NR_112400.1 TCCTGGTGTAGCGGTGAAATGCGCAGATATCAGGAGGAACACCGGTGGCGAAGGCGGATC 684

11993_B; ------------------------------------------------------------ 0

11993_A; tctgggccgatactgacgctgaggagcgaaagcgtggggagcgaacaggattagataccc 752

KY318506.1 TCTGGGCCGATACTGACGCTGAGGAGCGAAAGCGTGGGGAGCGAACAGGATTAGATACCC 764

MG198705.1 TCTGGGCCGATACTGACGCTGAGGAGCGAAAGCGTGGGGAGCGAACAGGATTAGATACCC 768

KY523106.1 TCTGGGCCGATACTGACGCTGAGGAGCGAAAGCGTGGGGAGCGAACAGGATTAGATACCC 709

MH430523.2 TCTGGGCCGATACTGACGCTGAGGAGCGAAAGCGTGGGGAGCGAACAGGATTAGATACCC 774

DQ026648.1 TCTGGGCCGATACTGACGCTGAGGAGCGAAAGCGTGGGGAGCGAACAGGATTAGATACCC 774

MT669274.1 TCTGGGCCGATACTGACGCTGAGGAGCGAAAGCGTGGGGAGCGAACAGGATTAGATACCC 714

MF077006.1 TCTGGGCCGATACTGACGCTGAGGAGCGAAAGCGTGGGGAGCGAACAGGATTAGATACCC 711

EU603342.1 TCTGGGCCGATACTGACGCTGAGGAGCGAAAGCGTGGGGAGCGAACAGGATTAGATACCC 735

JF827350.1 TCTGGGCCGATACTGACGCTGAGGAGCGAAAGCGTGGGGAGCGAACAGGATTAGATACCC 765

NR_112352.1 TCTGGGCCGATACTGACGCTGAGGAGCGAAAGCGTGGGGAGCGAACAGGATTAGATACCC 746

NR_024760.1 TCTGGGCCGATACTGACGCTGAGGAGCGAAAGCGTGGGGAGCGAACAGGATTAGATACCC 751

AJ007399.1 TCTGGGCCGATACTGACGCTGAGGAGCGAAAGCGTGGGGAGCGAACAGGATTAGATACCC 744

AB184480.1 TCTGGGCCGATACTGACGCTGAGGAGCGAAAGCGTGGGGAGCGAACAGGATTAGATACCC 744

MH021965.1 TCTGGGCCGATACTGACGCTGAGGAGCGAAAGCGTGGGGAGCGAACAGGATTAGATACCC 751

KT781120.1 TCTGGGCCGATACTGACGCTGAGGAGCGAAAGCGTGGGGAGCGAACAGGATTAGATACCC 741

NR_025622.1 TCTGGGCCGATACTGACGCTGAGGAGCGAAAGCGTGGGGAGCGAACAGGATTAGATACCC 741

NR_025621.1 TCTGGGCCGATACTGACGCTGAGGAGCGAAAGCGTGGGGAGCGAACAGGATTAGATACCC 741

EU741199.1 TCTGGGCCGATACTGACGCTGAGGAGCGAAAGCGTGGGGAGCGAACAGGATTAGATACCC 754

NR_112400.1 TCTGGGCCGATACTGACGCTGAGGAGCGAAAGCGTGGGGAGCGAACAGGATTAGATACCC 744

11993_B; ------------------------------------------------------------ 0

11993_A; tggtagtccacgccgtaaacgttgggaactaggtgtgggcgacattccacgtcgtccgtg 812

KY318506.1 TGGTAGTCCACGCCGTAAACGTTGGGAACTAGGTGTGGGCGACATTCCACGTCGTCCGTG 824

MG198705.1 TGGTAGTCCACGCCGTAAACGTTGGGAACTAGGTGTGGGCGACATTCCACGTTGTCCGTG 828

KY523106.1 TGGTAGTCCACGCCGTAAACGTTGGGAACTAGGTGTGGGCGACATTCCACGTTGTCCGTG 769

MH430523.2 TGGTAGTCCACGCCGTAAACGTTGGGAACTAGGTGTGGGCGACATTCCACGTTGTCCGTG 834

DQ026648.1 TGGTAGTCCACGCCGTAAACGTTGGGAACTAGGTGTGGGCGACATTCCACGTTGTCCGTG 834

MT669274.1 TGGTAGTCCACGCCGTAAACGTTGGGAACTAGGTGTGGGCGACATTCCACGTTGTCCGTG 774

MF077006.1 TGGTAGTCCACGCCGTAAACGTTGGGAACTAGGTGTGGGCGACATTCCACGTTGTCCGTG 771

EU603342.1 TGGTAGTCCACGCCGTAAACGTTGGGAACTAGGTGTGGGCGACATTCCACGTCGTCCGTG 795

JF827350.1 TGGTAGTCCACGCCGTAAACGTTGGGAACTAGGTGTGGGCGACATTCCACGTCGTCCGTG 825

NR_112352.1 TGGTAGTCCACGCCGTAAACGTTGGGAACTAGGTGTGGGCGACATTCCACGTCGTCCGTG 806

NR_024760.1 TGGTAGTCCACGCCGTAAACGTTGGGAACTAGGTGTGGGCGACATTCCACGTCGTCCGTG 811

AJ007399.1 TGGTAGTCCACGCCGTAAACGTTGGGAACTAGGTGTGGGCGACATTCCACGTCGTCCGTG 804

AB184480.1 TGGTAGTCCACGCCGTAAACGTTGGGAACTAGGTGTGGGCGACATTCCACGTCGTCCGTG 804

MH021965.1 TGGTAGTCCACGCCGTAAACGTTGGGAACTAGGTGTGGGCGACATTCCACGTCGTCCGTG 811

KT781120.1 TGGTAGTCCACGCCGTAAACGTTGGGAACTAGGTGTGGGCGACATTCCACGTCGTCCGTG 801

NR_025622.1 TGGTAGTCCACGCCGTAAACGTTGGGAACTAGGTGTGGGCGACATTCCACGTCGTCCGTG 801

NR_025621.1 TGGTAGTCCACGCCGTAAACGTTGGGAACTAGGTGTGGGCGACATTCCACGTCGTCCGTG 801

EU741199.1 TGGTAGTCCACGCCGTAAACGTTGGGAACTAGGTGTGGGCGACATTCCACGTCGTCCGTG 814

NR_112400.1 TGGTAGTCCACGCCGTAAACGTTGGGAACTAGGTGTGGGCGACATTCCACGTCGTCCGTG 804

11993_B; ------------------------------------------------------------ 0

11993_A; ccgcagctaacgcattaagttccccgcctggggagtacggccgcaaggctaaaactcaaa 872

KY318506.1 CCGCAGCTAACGCATTAAGTTCCCCGCCTGGGGAGTACGGCCGCAAGGCTAAAACTCAAA 884

MG198705.1 CCGCAGCTAACGCATTAAGTTCCCCGCCTGGGGAGTACGGCCGCAAGGCTAAAACTCAAA 888

KY523106.1 CCGCAGCTAACGCATTAAGTTCCCCGCCTGGGGAGTACGGCCGCAAGGCTAAAACTCAAA 829

MH430523.2 CCGCAGCTAACGCATTAAGTTCCCCGCCTGGGGAGTACGGCCGCAAGGCTAAAACTCAAA 894

DQ026648.1 CCGCAGCTAACGCATTAAGTTCCCCGCCTGGGGAGTACGGCCGCAAGGCTAAAACTCAAA 894

MT669274.1 CCGCAGCTAACGCATTAAGTTCCCCGCCTGGGGAGTACGGCCGCAAGGCTAAAACTCAAA 834

MF077006.1 CCGCAGCTAACGCATTAAGTTCCCCGCCTGGGGAGTACGGCCGCAAGGCTAAAACTCAAA 831

EU603342.1 CCGCAGCTAACGCATTAAGTTCCCCGCCTGGGGAGTACGGCCGCAAGGCTAAAACTCAAA 855

JF827350.1 CCGCAGCTAACGCATTAAGTTCCCCGCCTGGGGAGTACGGCCGCAAGGCTAAAACTCAAA 885

NR_112352.1 CCGCAGCTAACGCATTAAGTTCCCCGCCTGGGGAGTACGGCCGCAAGGCTAAAACTCAAA 866

NR_024760.1 CCGCAGCTAACGCATTAAGTTCCCCGCCTGGGGAGTACGGCCGCAAGGCTAAAACTCAAA 871

AJ007399.1 CCGCAGCTAACGCATTAAGTTCCCCGCCTGGGGAGTACGGCCGCAAGGCTAAAACTCAAA 864

AB184480.1 CCGCAGCTAACGCATTAAGTTCCCCGCCTGGGGAGTACGGCCGCAAGGCTAAAACTCAAA 864

MH021965.1 CCGCAGCTAACGCATTAAGTTCCCCGCCTGGGGAGTACGGCCGCAAGGCTAAAACTCAAA 871

KT781120.1 CCGCAGCTAACGCATTAAGTTCCCCGCCTGGGGAGTACGGCCGCAAGGCTAAAACTCAAA 861

NR_025622.1 CCGCAGCTAACGCATTAAGTTCCCCGCCTGGGGAGTACGGCCGCAAGGCTAAAACTCAAA 861

NR_025621.1 CCGCAGCTAACGCATTAAGTTCCCCGCCTGGGGAGTACGGCCGCAAGGCTAAAACTCAAA 861

EU741199.1 CCGCAGCTAACGCATTAAGTTCCCCGCCTGGGGAGTACGGCCGCAAGGCTAAAACTCAAA 874

NR_112400.1 CCGCAGCTAACGCATTAAGTTCCCCGCCTGGGGAGTACGGCCGCAAGGCTAAAACTCAAA 864

11993_B; ------------------------------------------------------------ 0

11993_A; ggaattgacgggggcccgcacaagcggcggagcatgtggcttaattcgacgcaacgcgaa 932

KY318506.1 GGAATTGACGGGGGCCCGCACAAGCGGCGGAGCATGTGGCTTAATTCGACGCAACGCGAA 944

MG198705.1 GGAATTGACGGGGGCCCGCACAAGCGGCGGAGCATGTGGCTTAATTCGACGCAACGCGAA 948

KY523106.1 GGAATTGACGGGGGCCCGCACAAGCGGCGGAGCATGTGGCTTAATTCGACGCAACGCGAA 889

MH430523.2 GGAATTGACGGGGGCCCGCACAAGCGGCGGAGCATGTGGCTTAATTCGACGCAACGCGAA 954

DQ026648.1 GGAATTGACGGGGGCCCGCACAAGCGGCGGAGCATGTGGCTTAATTCGACGCAACGCGAA 954

MT669274.1 GGAATTGACGGGGGCCCGCACAAGCGGCGGAGCATGTGGCTTAATTCGACGCAACGCGAA 894

MF077006.1 GGAATTGACGGGGGCCCGCACAAGCGGCGGAGCATGTGGCTTAATTCGACGCAACGCGAA 891

EU603342.1 GGAATTGACGGGGGCCCGCACAAGCAGCGGAGCATGTGGCTTAATTCGACGCAACGCGAA 915

JF827350.1 GGAATTGACGGGGGCCCGCACAAGCAGCGGAGCATGTGGCTTAATTCGACGCAACGCGAA 945

NR_112352.1 GGAATTGACGGGGGCCCGCACAAGCAGCGGAGCATGTGGCTTAATTCGACGCAACGCGAA 926

NR_024760.1 GGAATTGACGGGGGCCCGCACAAGCGGCGGAGCATGTGGCTTAATTCGACGCAACGCGAA 931

AJ007399.1 GGAATTGACGGGGGCCCGCACAAGCGGCGGAGCATGTGGCTTAATTCGACGCAACGCGAA 924

AB184480.1 GGAATTGACGGGGGCCCGCACAAGCGGCGGAGCATGTGGCTTAATTCGACGCAACGCGAA 924

MH021965.1 GGAATTGACGGGGGCCCGCACAAGCGGCGGAGCATGTGGCTTAATTCGACGCAACGCGAA 931

KT781120.1 GGAATTGACGGGGGCCCGCACAAGCGGCGGAGCATGTGGCTTAATTCGACGCAACGCGAA 921

NR_025622.1 GGAATTGACGGGGGCCCGCACAAGCGGCGGAGCATGTGGCTTAATTCGACGCAACGCGAA 921

NR_025621.1 GGAATTGACGGGGGCCCGCACAAGCGGCGGAGCATGTGGCTTAATTCGACGCAACGCGAA 921

EU741199.1 GGAATTGACGGGGGCCCGCACAAGCGGCGGAGCATGTGGCTTAATTCGACGCAACGCGAA 934

NR_112400.1 GGAATTGACGGGGGCCCGCACAAGCGGCGGAGCATGTGGCTTAATTCGACGCAACGCGAA 924

11993_B; ------------------------------------------------------------ 0

11993_A; gaaccttaccaaggcttgacatacaccggaaaaccctggagacagggtcccccttgtggt 992

KY318506.1 GAACCTTACCAAGGCTTGACATACACCGGAAAACCGTGGAGACACGGTCCCCCTTGTGGT 1004

MG198705.1 GAACCTTACCAAGGCTTGACATACACCGGAAAACCGTGGAGACACGGTCCCCCTTGTGGT 1008

KY523106.1 GAACCTTACCAAGGCTTGACATACACCGGAAAACCGTGGAGACACGGTCCCCCTTGTGGT 949

MH430523.2 GAACCTTACCAAGGCTTGACATACACCGGAAAACCGTGGAGACACGGTCCCCCTTGTGGT 1014

DQ026648.1 GAACCTTACCAAGGCTTGACATACACCGGAAAACCGTGGAGACACGGTCCCCCTTGTGGT 1014

MT669274.1 GAACCTTACCAAGGCTTGACATACACCGGAAAACCGTGGAGACACGGTCCCCCTTGTGGT 954

MF077006.1 GAACCTTACCAAGGCTTGACATACACCGG------------------------------- 920

EU603342.1 GAACCTTACCAAGGCTTGACATACACCGGAAAACCCTGGAGACAGGGTCCCCCTTGTGGT 975

JF827350.1 GAACCTTACCAAGGCTTGACATACACCGGAAAACCCTGGAGACAGGGTCCCCCTTGTGGT 1005

NR_112352.1 GAACCTTACCAAGGCTTG------------------------------------------ 944

NR_024760.1 GAACCTTACCAAGGCTTGACATACACCGGAAAACCCTGGAGACAGGGTCCCCCTTGTGGT 991

AJ007399.1 GAACCTTACCAAGGCTTGACATACACCGGAAAACCCTGGAGACAGGGTCCCCCTTGTGGT 984

AB184480.1 GAACCTTACCAAGGCTTGACATACACCGGAAAACCCTGGAGACAGGGTCCCCCTTGTGGT 984

MH021965.1 GAACCTTACCAAGGCTTGACATACACCGGAAACGTCTGGAGACAGGCGCCCCCTTGTGGT 991

KT781120.1 GAACCTTACCAAGGCTTGACATACACCGGAAAACCCTGGAGACAGGGTCCCCCTTGTGGT 981

NR_025622.1 GAACCTTACCAAGGCTTGACATACACCGGAAAACCCTGGAGACAGGGTCCCCCTTGTGGT 981

NR_025621.1 GAACCTTACCAAGGCTTGACATACACCGGAAAACCCTGGAGACAGGGTCCCCCTTGTGGT 981

EU741199.1 GAACCTTACCAAGGCTTGACATACACCGGAAAACCCTGGAGACAGGGTCCCCCTTGTGGT 994

NR_112400.1 GAACCTTACCAAGGCTTGACATACACCGGAAAACCCTGGAGACAGGGTCCCCCTTGTGGT 984

11993_B; ------------------------------------------------------------ 0

11993_A; cggtgtacaggtggtgcatggctgtcgtcagctcgtgtcgtgagatgttgggttaagtcc 1052

KY318506.1 CGGTGTACAGGTGGTGCATGGCTGTCGTCAGCTCGTGTCGTGAGATGTTGGGTTAAGTCC 1064

MG198705.1 CGGTGTACAGGTGGTGCATGGCTGTCGTCAGCTCGTGTCGTGAGATGTTGGGTTAAGTCC 1068

KY523106.1 CGGTGTACAGGTGGTGCATGGCTGTCGTCAGCTCGTGTCGTGAGATGTTGGGTTAAGTCC 1009

MH430523.2 CGGTGTACAGGTGGTGCATGGCTGTCGTCAGCTCGTGTCGTGAGATGTTGGGTTAAGTCC 1074

DQ026648.1 CGGTGTACAGGTGGTGCATGGCTGTCGTCAGCTCGTGTCGTGAGATGTTGGGTTAAGTCC 1074

MT669274.1 CGGTGTACAGGTGGTGCATGGCTGTCGTCAGCTCGTGTCGTGAGATGTTGGGTTAAGTCC 1014

MF077006.1 ------------------------------------------------------------ 920

EU603342.1 CGGTGTACAGGTGGTGCATGGCTGTCGTCAGCTCGTGTCGTGAGATGTTGGGTTAAGTCC 1035

JF827350.1 CGGTGTACAGGTGGTGCATGGCTGTCGTCAGCTCGTGTCGTGAGATGTTGGGTTAAGTCC 1065

NR_112352.1 ------------------------------------------------------------ 944

NR_024760.1 CGGTGTACAGGTGGTGCATGGCTGTCGTCAGCTCGTGTCGTGAGATGTTGGGTTAAGTCC 1051

AJ007399.1 CGGTGTACAGGTGGTGCATGGCTGTCGTCAGCTCGTGTCGTGAGATGTTGGGTTAAGTCC 1044

AB184480.1 CGGTGTACAGGTGGTGCATGGCTGTCGTCAGCTCGTGTCGTGAGATGTTGGGTTAAGTCC 1044

MH021965.1 CGGTGTACAGGTGGTGCATGGCTGTCGTCAGCTCGTGTCGTGAGATGTTGGGTTAAGTCC 1051

KT781120.1 CGGTGTACAGGTGGTGCATGGCTGTCGTCAGCTCGTGTCGTGAGATGTTGGGTTAAGTCC 1041

NR_025622.1 CGGTGTACAGGTGGTGCATGGCTGTCGTCAGCTCGTGTCGTGAGATGTTGGGTTAAGTCC 1041

NR_025621.1 CGGTGTACAGGTGGTGCATGGCTGTCGTCAGCTCGTGTCGTGAGATGTTGGGTTAAGTCC 1041

EU741199.1 CGGTGTACAGGTGGTGCATGGCTGTCGTCAGCTCGTGTCGTGAGATGTTGGGTTAAGTCC 1054

NR_112400.1 CGGTGTACAGGTGGTGCATGGCTGTCGTCAGCTCGTGTCGTGAGATGTTGGGTTAAGTCC 1044

11993_B; ------------------------------------------------------------ 0

11993_A; cgcaacgagcgcaacccttgttctgtgttgccagcatgcccttc--ggggtgatggggac 1110

KY318506.1 CGCAACGAGCGCAACCCTTGTTCTGTGTTGCCAGCATGCCCTTC--GGGGTGATGGGGAC 1122

MG198705.1 CGCAACGAGCGCAACCCTTGTTCTGTGTTGCCAGCATGCCTTTC--GGGGTGATGGGGAC 1126

KY523106.1 CGCAACGAGCGCAACCCTTGTTCTGTGTTGCCAGCATGCCTTTC--GGGGTGATGGGGAC 1067

MH430523.2 CGCAACGAGCGCAACCCTTGTTCTGTGTTGCCAGCATGCCTTTC--GGGGTGATGGGGAC 1132

DQ026648.1 CGCAACGAGCGCAACCCTTGTTCTGTGTTGCCAGCATGCCTTTC--GGGGTGATGGGGAC 1132

MT669274.1 CGCAACGAGCGCAACCCTTGTTCTGTGTTGCCAGCATGCCTTTC--GGGGTGATGGGGAC 1072

MF077006.1 ------------------------------------------------------------ 920

EU603342.1 CGCAACGAGCGCAACCCTTGTTCTGTGTTGCCAGCATGCCCTTC--GGGGTGATGGGGAC 1093

JF827350.1 CGCAACGAGCGCAACCCTTGTTCTGTGTTGCCAGCATGCCCTTC--GGGGTGATGGGGAC 1123

NR_112352.1 ------------------------------------------------------------ 944

NR_024760.1 CGCAACGAGCGCAACCCTTGTTCTGTGTTGCCAGCACGTCCTTTCGGGGATGGTGGGGAC 1111

AJ007399.1 CGCAACGAGCGCAACCCTTGTTCTGTGTTGCCAGCATGCCCTTC--GGGGTGATGGGGAC 1102

AB184480.1 CGCAACGAGCGCAACCCTTGTTCTGTGTTGCCAGCATGCCCTTC--GGGGTGATGGGGAC 1102

MH021965.1 CGCAACGAGCGCAACCCTTGTTCTGTGTTGCCAGCATGCCCTTC--GGGGTGATGGGGAC 1109

KT781120.1 CGCAACGAGCGCAACCCTTGTTCTGTGTTGCCAGCATGCCCTTC--GGGGTGATGGGGAC 1099

NR_025622.1 CGCAACGAGCGCAACCCTTGTTCTGTGTTGCCAGCATGCCCTTC--GGGGTGATGGGGAC 1099

NR_025621.1 CGCAACGAGCGCAACCCTTGTTCTGTGTTGCCAGCATGCCCTTC--GGGGTGATGGGGAC 1099

EU741199.1 CGCAACGAGCGCAACCCTTGTTCTGTGTTGCCAGCATGCCCTTC--GGGGTGATGGGGAC 1112

NR_112400.1 CGCAACGAGCGCAACCCTTGTTCTGTGTTGCCAGCATGCCCTTC--GGGGTGATGGGGAC 1102

11993_B; ------------------------------------------------------------ 0

11993_A; tcacaggagactgccggggtcaactcggaggaaggtggggacgacgtcaagtcatcatgc 1170

KY318506.1 TCACAGGAGACTGCCGGGGTCAACTCGGAGGAAGGTGGGGACGACGTCAAGTCATCATGC 1182

MG198705.1 TCACAGGAGACTGCCGGGGTCAACTCGGAGGAAGGTGGGGACGACGTCAAGTCATCATGC 1186

KY523106.1 TCACAGGAGACTGCCGGGGTCAACTCGGAGGAAGGTGGGGACGACGTCAAGTCATCATGC 1127

MH430523.2 TCACAGGAGACTGCCGGGGTCAACTCGGAGGAAGGTGGGGACGACGTCAAGTCATCATGC 1192

DQ026648.1 TCACAGGAGACTGCCGGGGTCAACTCGGAGGAAGGTGGGGACGACGTCAAGTCATCATGC 1192

MT669274.1 TCACAGGAGACTGCCGGGGTCAACTCGGAGGAAGGTGGGGACGACGTCAAGTCATCATGC 1132

MF077006.1 ------------------------------------------------------------ 920

EU603342.1 TCACAGGAGACTGCCGGGGTCAACTCGGAGGAAGGTGGGGACGACGTCAAGTCATCATGC 1153

JF827350.1 TCACAGGAGACTGCCGGGGTCAACTCGGAGGAAGGTGGGGACGACGTCAAGTCATCATGC 1183

NR_112352.1 ------------------------------------------------------------ 944

NR_024760.1 TCACAGGAGACTGCCGGGGTCAACTCGGAGGAAGGTGGGGACGACGTCAAGTCATCATGC 1171

AJ007399.1 TCACAGGAGACTGCCGGGGTCAACTCGGAGGAAGGTGGGGACGACGTCAAGTCATCATGC 1162

AB184480.1 TCACAGGAGACTGCCGGGGTCAACTCGGAGGAAGGTGGGGACGACGTCAAGTCATCATGC 1162

MH021965.1 TCACAGGAGACTGCCGGGGTCAACTCGGAGGAAGGTGGGGACGACGTCAAGTCATCATGC 1169

KT781120.1 TCACAGGAGACTGCCGGGGTCAACTCGGAGGAAGGTGGGGACGACGTCAAGTCATCATGC 1159

NR_025622.1 TCACAGGAGACTGCCGGGGTCAACTCGGAGGAAGGTGGGGACGACGTCAAGTCATCATGC 1159

NR_025621.1 TCACAGGAGACTGCCGGGGTCAACTCGGAGGAAGGTGGGGACGACGTCAAGTCATCATGC 1159

EU741199.1 TCACAGGAGACTGCCGGGGTCAACTCGGAGGAAGGTGGGGACGACGTCAAGTCATCATGC 1172

NR_112400.1 TCACAGGAGACTGCCGGGGTCAACTCGGAGGAAGGTGGGGACGACGTCAAGTCATCATGC 1162

11993_B; ------------------------------------------------------------ 0

11993_A; cccttatgtcttgggctgcacacgtgctacaatggccggtacaatgagctgcgataccgc 1230

KY318506.1 CCCTTATGTCTTGGGCTGCACACGTGCTACAATGGCCGGTACAATGAGCTGCGATACCGC 1242

MG198705.1 CCCTTATGTCTTGGGCTGCACACGTGCTACAATGGCCGGTACAATGAGCTGCGATACCGC 1246

KY523106.1 CCCTTATGTCTTGGGCTGCACACGTGCTACAATGGCCGGTACAATGAGCTGCGATACCGT 1187

MH430523.2 CCCTTATGTCTTGGGCTGCACACGTGCTACAATGGCCGGTACAATGAGCTGCGATACCGT 1252

DQ026648.1 CCCTTATGTCTTGGGCTGCACACGTGCTACAATGGCCGGTACAATGAGCTGCGATACCGC 1252

MT669274.1 CCCTTATGTCTTGGGCTGCACACGTGCTACAATGGCCGGTACAATGAGCTGCGATACCGC 1192

MF077006.1 ------------------------------------------------------------ 920

EU603342.1 CCCTTATGTCTTGGGCTGCACACGTGCTACAATGGCCGGTACAATGAGCTGCGATACCGC 1213

JF827350.1 CCCTTATGTCTTGGGCTGCACACGTGCTACAATGGCCGGTACAATGAGCTGCGATACCGC 1243

NR_112352.1 ------------------------------------------------------------ 944

NR_024760.1 CCCTTATGTCTTGGGCTGCACACGTGCTACAATGGCCGGTACAATGAGCTGCGATACCGC 1231

AJ007399.1 CCCTTATGTCTTGGGCTGCACACGTGCTACAATGGCCGGTACAATGAGCTGCGATACCGC 1222

AB184480.1 CCCTTATGTCTTGGGCTGCACACGTGCTACAATGGCCGGTACAATGAGCTGCGATACCGC 1222

MH021965.1 CCCTTATGTCTTGGGCTGCACACGTGCTACAATGGCCGGTACAATGAGCTGCGATACCGC 1229

KT781120.1 CCCTTATGTCTTGGGCTGCACACGTGCTACAATGGCCGGTACAATGAGCTGCGATACCGC 1219

NR_025622.1 CCCTTATGTCTTGGGCTGCACACGTGCTACAATGGCCGGTACAATGAGCTGCGATACCGC 1219

NR_025621.1 CCCTTATGTCTTGGGCTGCACACGTGCTACAATGGCCGGTACAATGAGCTGCGATACCGC 1219

EU741199.1 CCCTTATGTCTTGGGCTGCACACGTGCTACAATGGCCGGTACAATGAGCTGCGATACCGC 1232

NR_112400.1 CCCTTATGTCTTGGGCTGCACACGTGCTACAATGGCCGGTACAATGAGCTGCGATACCGC 1222

11993_B; ------------------------------------------------------------ 0

11993_A; gaggtggagcgaatctcaaaaagccggtctcagttcggattggggtctgcaactcgaccc 1290

KY318506.1 GAGGTGGAGCGAATCTCAAAAAGCCGGTCTCAGTTCGGATTGGGGTCTGCAACTCGACCC 1302

MG198705.1 GAGGTGGAGCGAATCTCAAAAAGCCGGTCTCAGTTCGGATTGGGGTCTGCAACTCGACCC 1306

KY523106.1 GAGGTGGAGCGAATCTCAAAAAGCCGGTCTCAGTTCGGATTGGGGTCTGCAACTCGACCC 1247

MH430523.2 GAGGTGGAGCGAATCTCAAAAAGCCGGTCTCAGTTCGGATTGGGGTCTGCAACTCGACCC 1312

DQ026648.1 GAGGTGGAGCGAATCTCAAAAAGCCGGTCTCAGTTCGGATTGGGGTCTGCAACTCGACCC 1312

MT669274.1 GAGGTGGAGCGAATCTCAAAAAGCCGGTCTCAGTTCGGATTGGGGTCTGCAACTCGACCC 1252

MF077006.1 ------------------------------------------------------------ 920

EU603342.1 GAGGTGGAGCGAATCTCAAAAAGCCGGTCTCAGTTCGGATTGGGGTCTGCAACTCGACCC 1273

JF827350.1 GAGGTGGAGCGAATCTCAAAAAGCCGGTCTCAGTTCGGATTGGGGTCTGCAACTCGACCC 1303

NR_112352.1 ------------------------------------------------------------ 944

NR_024760.1 GAGGTGGAGCGAATCTCAAAAAGCCGGTCTCAGTTCGGATTGGGGTCTGCAACTCGACCC 1291

AJ007399.1 GAGGTGGAGCGAATCTCAAAAAGCCGGTCTCAGTTCGGATTGGGGTCTGCAACTCGACCC 1282

AB184480.1 GAGGTGGAGCGAATCTCAAAAAGCCGGTCTCAGTTCGGATTGGGGTCTGCAACTCGACCC 1282

MH021965.1 GAGGTGGAGCGAATCTCAAAAAGCCGGTCTCAGTTCGGATTGGGGTCTGCAACTCGACCC 1289

KT781120.1 GAGGTGGAGCGAATCTCAAAAAGCCGGTCTCAGTTCGGATTGGGGTCTGCAACTCGACCC 1279

NR_025622.1 GAGGTGGAGCGAATCTCAAAAAGCCGGTCTCAGTTCGGATTGGGGTCTGCAACTCGACCC 1279

NR_025621.1 GAGGTGGAGCGAATCTCAAAAAGCCGGTCTCAGTTCGGATTGGGGTCTGCAACTCGACCC 1279

EU741199.1 GAGGTGGAGCGAATCTCAAAAAGCCGGTCTCAGTTCGGATTGGGGTCTGCAACTCGACCC 1292

NR_112400.1 GAGGTGGAGCGAATCTCAAAAAGCCGGTCTCAGTTCGGATTGGGGTCTGCAACTCGACCC 1282

11993_B; ------------------------------------------------------------ 0

11993_A; catgaagtcggagtcgctagtaatcgcagatcagcattgctgcggtgaatacgttcccgg 1350

KY318506.1 CATGAAGTCGGAGTCGCTAGTAATCGCAGATCAGCATTGCTGCGGTGAATACGTTCCCGG 1362

MG198705.1 CATGAAGTCGGAGTCGCTAGTAATCGCAGATCAGCATTGCTGCGGTGAATACGTTCCCGG 1366

KY523106.1 CATGAAGTCGGAGTCGCTAGTAATCGCAGATCAGCATTGCTGCGGTGAATACGTTCCCGG 1307

MH430523.2 CATGAAGTCGGAGTCGCTAGTAATCGCAGATCAGCATTGCTGCGGTGAATACGTTCCCGG 1372

DQ026648.1 CATGAAGTCGGAGTCGCTAGTAATCGCAGATCAGCATTGCTGCGGTGAATACGTTCCCGG 1372

MT669274.1 CATGAAGTCGGAGTCGCTAGTAATCGCAGATCAGCATTGCTGCGGTGAATACGTTCCCGG 1312

MF077006.1 ------------------------------------------------------------ 920

EU603342.1 CATGAAGTCGGAGTTGCTAGTAATCGCAGATCAGCATTGCTGCGGTGAATACGTTCCCGG 1333

JF827350.1 CATGAAGTCGGAGTCGCTAGTAATCGCAGATCAGCATTGCTGCGGTGAATACGTTCCCGG 1363

NR_112352.1 ------------------------------------------------------------ 944

NR_024760.1 CATGAAGTCGGAGTCGCTAGTAATCGCAGATCAGCATTGCTGCGGTGAATACGTTCCCGG 1351

AJ007399.1 CATGAAGTCGGAGTCGCTAGTAATCGCAGATCAGCATTGCTGCGGTGAATACGTTCCCGG 1342

AB184480.1 CATGAAGTCGGAGTCGCTAGTAATCGCAGATCAGCATTGCTGCGGTGAATACGTTCCCGG 1342

MH021965.1 CATGAAGTCGGAGTCGCTAGTAATCGCAGATCAGCATTGCTGCGGTGAATACGTTCCCGG 1349

KT781120.1 CATGAAGTCGGAGTCGCTAGTAATCGCAGATCAGCATTGCTGCGGTGAATACGTTCCCGG 1339

NR_025622.1 CATGAAGTCGGAGTCGCTAGTAATCGCAGATCAGCATTGCTGCGGTGAATACGTTCCCGG 1339

NR_025621.1 CATGAAGTCGGAGTCGCTAGTAATCGCAGATCAGCATTGCTGCGGTGAATACGTTCCCGG 1339

EU741199.1 CATGAAGTCGGAGTCGCTAGTAATCGCAGATCAGCATTGCTGCGGTGAATACGTTCCCGG 1352

NR_112400.1 CATGAAGTCGGAGTCGCTAGTAATCGCAGATCAGCATTGCTGCGGTGAATACGTTCCCGG 1342

11993_B; ------------------------------------------------------------ 0

11993_A; gccttgtacacaccgcccgtcacgtcacgaaagtcggtaacacccgaagccggtggccca 1410

KY318506.1 GCCTTGTACACACCGCCCGTCACGTCACGAAAGTCGGTAACACCCGAAGCCGGTGGCCCA 1422

MG198705.1 GCCTTGTACACACCGCCCGTCACGTCACGAAAGTCGGTAACACCCGAAGCCGGTGGCCAA 1426

KY523106.1 GCCTTGTACACACCGCCCGTCACGTCACGAAAGTCGGTAACACCCGAAGCCGGTGGCCCA 1367

MH430523.2 GCCTTGTACACACCGCCCGTCACGTCACGAAAGTCGGTAACACCCGAAGCCGGTGGCCCA 1432

DQ026648.1 GCCTTGTACACACCGCCCGTCACGTCACGAAAGTCGGTAACACCCGAAGCCGGTGGCCCA 1432

MT669274.1 GCCTTGTACACACCGCCCGTCACGTCACGAAAGTCGGTAACACCCGAAGCCGGTGGCCCA 1372

MF077006.1 ------------------------------------------------------------ 920

EU603342.1 GCCTTGTACACACCGCCCGTCACGTCACGAAAGTCGGTAACACCCGAAGCCGGTGGCCCA 1393

JF827350.1 GCCTTGTACACACCGCCCGTCACGTCACGAAAGTCGGTAACACCCGAAGCCGGTGGCCCA 1423

NR_112352.1 ------------------------------------------------------------ 944

NR_024760.1 GCCTTGTACACACCGCCCGTCACGTCACGAAAGTCGGTAACACCCGAAGCCGGTGGCCCA 1411

AJ007399.1 GCCTTGTACACACCGCCCGTCACGTCACGAAAGTCGGTAACACCCGAAGCCGGTGGCCCA 1402

AB184480.1 GCCTTGTACACACCGCCCGTCACGTCACGAAAGTCGGTAACACCCGAAGCCGGTGGCCCA 1402

MH021965.1 GCCTTGTACACACCGCCCGTCACGTCACGAAAGTCGGTAACACCCGAAGCCGGTGGCCCA 1409

KT781120.1 GCCTTGTACACACCGCCCGTCACGTCACGAAAGTCGGTAACACCCGAAGCCGGTGGCCCA 1399

NR_025622.1 GCCTTGTACACACCGCCCGTCACGTCACGAAAGTCGGTAACACCCGAAGCCGGTGGCCCA 1399

NR_025621.1 GCCTTGTACACACCGCCCGTCACGTCACGAAAGTCGGTAACACCCGAAGCCGGTGGCCCA 1399

EU741199.1 GCCTTGTACACACCGCCCGTCACGTCACGAAAGTCGGTAACACCCGAAGCCGGTGGCCCA 1412

NR_112400.1 GCCTTGTACACACCGCCCGTCACGTCACGAAAGTCGGTAACACCCGAAGCCGGTGGCCCA 1402

11993_B; ---------------------acccacagtagctgctcaggacgaacgctggcggcgtgc 39

11993_A; accccttg-tgaggactgtgBacccacagtagctgctcaggacgaacgctggcggcgtgc 1469

KY318506.1 ACCCCTTG-TGGGAGGGAATCGTCGAAGGTGGGACTGGCGATTGGGA--------CGAAG 1473

MG198705.1 CCCTCGGG-ACGGATGTCGAGTGAGCTT-------------------------------- 1453

KY523106.1 ACCCCTTG-TGGGAGGGAATCGTCGAAGGTGGGACTGGCGATTGGAC--------G---- 1414

MH430523.2 ACCCCTTG-TGGGAGGGAATCGTCGAAGGTGGGACTGGCGATTGGGA--------CGAAG 1483

DQ026648.1 ACCCCTTG-TGGGAGGGAATCGTCGAAGGTGGGACTGGCGATTGGGA--------CGAAG 1483

MT669274.1 ACCCCTTG-TGGGAGGGAATCGTCG----------------------------------- 1396

MF077006.1 ------------------------------------------------------------ 920

EU603342.1 ACCC-CTTGTGGGAGGGAATCGTCGAAGGTGAC--------------------------- 1425

JF827350.1 ACCC-CTTGTGGGAGGGAATCGTCGAAGGTGGGACTGGCGATTGGGA--------CGAAG 1474

NR_112352.1 ------------------------------------------------------------ 944

NR_024760.1 ACCC-CTTGTGGGAGGGAATCGTCGAAGGTGGGACTGGCGATTGGGA--------CGAAG 1462

AJ007399.1 ACCC-CTTGTGGGAGGGAGCTGTCGAAGGTGGGACTGGCGATTGGGA--------CGAAG 1453

AB184480.1 ACCC-CTTGTGGGAGGGAATCGTCGAAGGTGGGACTGGCGATTGGGA--------CGAAG 1453

MH021965.1 ACCC-CTTGTGGGAGGGAATCGTCGAAGGTGGGACTGGCGATTGGGA--------CGAAG 1460

KT781120.1 ACCCCTTTGTGGGAGGGAATCGTCGAAGGTGGGACTGGCGATTGGGA--------CGAAG 1451

NR_025622.1 ACCC-CTTGTGGGAGGGAATCGTCGAAGGTGGGACTGGCGATTGGGA--------CGAAG 1450

NR_025621.1 ACCC-CTTGTGGGAGGGAATCGTCGAAGGTGGGACTGGCGATTGGGA--------CGAAG 1450

EU741199.1 ACCC-CTTGTGGGAGGGAATCGTCGAAGGTGGGACTGGCGATTGGGA--------CGAAG 1463

NR_112400.1 ACCC-CTTGTGGGAGGGAATCGTCGAAGGTGGGACTGGCGATTGGGA--------CGAAG 1453

11993_B; ttaacacatgcaagtcga-----acgatgaagccgcttcggtggtggattagtggcgaac 94

11993_A; ttaacacatgcaagtcga-----acgatgaagccgcttcggtggtggattagtggcgaac 1524

KY318506.1 TCGTAACAAGGTAGCCGTACCGGAAGGTGCGGCTGGATCACCT---CCTT---------- 1520

MG198705.1 ------------------------------------------------------------ 1453

KY523106.1 ------------------------------------------------------------ 1414

MH430523.2 TCGTAACAAGGTAGCCGTACCGGAAGGTGCGGCTGGATCACCT---CCTTTCTA------ 1534

DQ026648.1 TCGTAACAAGGTAGCCGTACCGGAAGGTGC------------------------------ 1513

MT669274.1 ------------------------------------------------------------ 1396

MF077006.1 ------------------------------------------------------------ 920

EU603342.1 ------------------------------------------------------------ 1425

JF827350.1 TCGTAACAAGGTA----------------------------------------------- 1487

NR_112352.1 ------------------------------------------------------------ 944

NR_024760.1 TCGTAACAAGGTAGCCGTACCGGAAGG--------------------------------- 1489

AJ007399.1 TCGTAACAAGGTAGCCGTACCCGAAGGTGC------------------------------ 1483

AB184480.1 TCGTAACAAGGTAGCCGTACCGGAAG---------------------------------- 1479

MH021965.1 TCGTAACAAGGTA----------------------------------------------- 1473

KT781120.1 TCGTAACAAGGTAGCCGTACCGGAAGGTGCGGCTGGATCACCTCCTT------------- 1498

NR_025622.1 TCGTAACAAGGTAGCCGTACCGGAAGGTGCGGCTGGATCACCTCCT-------------- 1496

NR_025621.1 TCGTAACAAGGTAGCCGTACCGGAAGGTGCGGCTGGATCACCTCCTT------------- 1497

EU741199.1 TCGTAACAAGGTAGCCGTACCGGAAGGTGCGGCTGG------------------------ 1499

NR_112400.1 TCGTAACAAGGTAGCCGTACCGGAAGG--------------------------------- 1480

11993_B; gggtgagtaacacgtgggcaatctgccctgcactctgggacaagccctggaaacggggtc 154

11993_A; gggtgagtaacacgtgggcaatctgccctgcactctgggacaagccctggaaacggggtc 1584

KY318506.1 ------------------------------------------------------------ 1520

MG198705.1 ------------------------------------------------------------ 1453

KY523106.1 ------------------------------------------------------------ 1414

MH430523.2 ------------------------------------------------------------ 1534

DQ026648.1 ------------------------------------------------------------ 1513

MT669274.1 ------------------------------------------------------------ 1396

MF077006.1 ------------------------------------------------------------ 920

EU603342.1 ------------------------------------------------------------ 1425

JF827350.1 ------------------------------------------------------------ 1487

NR_112352.1 ------------------------------------------------------------ 944

NR_024760.1 ------------------------------------------------------------ 1489

AJ007399.1 ------------------------------------------------------------ 1483

AB184480.1 ------------------------------------------------------------ 1479

MH021965.1 ------------------------------------------------------------ 1473

KT781120.1 ------------------------------------------------------------ 1498

NR_025622.1 ------------------------------------------------------------ 1496

NR_025621.1 ------------------------------------------------------------ 1497

EU741199.1 ------------------------------------------------------------ 1499

NR_112400.1 ------------------------------------------------------------ 1480

11993_B; taataccggatacgactgcctgaggcatctcgggtggtggaaagctccggcggtgcagga 214

11993_A; taataccggatacgactgcctgaggcatctcgggtggtggaaagctccggcggtgcagga 1644

KY318506.1 ------------------------------------------------------------ 1520

MG198705.1 ------------------------------------------------------------ 1453

KY523106.1 ------------------------------------------------------------ 1414

MH430523.2 ------------------------------------------------------------ 1534

DQ026648.1 ------------------------------------------------------------ 1513

MT669274.1 ------------------------------------------------------------ 1396

MF077006.1 ------------------------------------------------------------ 920

EU603342.1 ------------------------------------------------------------ 1425

JF827350.1 ------------------------------------------------------------ 1487

NR_112352.1 ------------------------------------------------------------ 944

NR_024760.1 ------------------------------------------------------------ 1489

AJ007399.1 ------------------------------------------------------------ 1483

AB184480.1 ------------------------------------------------------------ 1479

MH021965.1 ------------------------------------------------------------ 1473

KT781120.1 ------------------------------------------------------------ 1498

NR_025622.1 ------------------------------------------------------------ 1496

NR_025621.1 ------------------------------------------------------------ 1497

EU741199.1 ------------------------------------------------------------ 1499

NR_112400.1 ------------------------------------------------------------ 1480

11993_B; tgagcccgcggcctatcagcttgttggtggggtaatggcctaccaaggcgacgacgggta 274

11993_A; tgagcccgcggcctatcagcttgttggtggggtaatggcctaccaaggcgacgacgggta 1704

KY318506.1 ------------------------------------------------------------ 1520

MG198705.1 ------------------------------------------------------------ 1453

KY523106.1 ------------------------------------------------------------ 1414

MH430523.2 ------------------------------------------------------------ 1534

DQ026648.1 ------------------------------------------------------------ 1513

MT669274.1 ------------------------------------------------------------ 1396

MF077006.1 ------------------------------------------------------------ 920

EU603342.1 ------------------------------------------------------------ 1425

JF827350.1 ------------------------------------------------------------ 1487

NR_112352.1 ------------------------------------------------------------ 944

NR_024760.1 ------------------------------------------------------------ 1489

AJ007399.1 ------------------------------------------------------------ 1483

AB184480.1 ------------------------------------------------------------ 1479

MH021965.1 ------------------------------------------------------------ 1473

KT781120.1 ------------------------------------------------------------ 1498

NR_025622.1 ------------------------------------------------------------ 1496

NR_025621.1 ------------------------------------------------------------ 1497

EU741199.1 ------------------------------------------------------------ 1499

NR_112400.1 ------------------------------------------------------------ 1480

11993_B; gccggcctgagagggcgaccggccacactgggactgagacacggcccagactcctacggg 334

11993_A; gccggcctgagagggcgaccggccacactgggactgagacacggcccagactcctacggg 1764

KY318506.1 ------------------------------------------------------------ 1520

MG198705.1 ------------------------------------------------------------ 1453

KY523106.1 ------------------------------------------------------------ 1414

MH430523.2 ------------------------------------------------------------ 1534

DQ026648.1 ------------------------------------------------------------ 1513

MT669274.1 ------------------------------------------------------------ 1396

MF077006.1 ------------------------------------------------------------ 920

EU603342.1 ------------------------------------------------------------ 1425

JF827350.1 ------------------------------------------------------------ 1487

NR_112352.1 ------------------------------------------------------------ 944

NR_024760.1 ------------------------------------------------------------ 1489

AJ007399.1 ------------------------------------------------------------ 1483

AB184480.1 ------------------------------------------------------------ 1479

MH021965.1 ------------------------------------------------------------ 1473

KT781120.1 ------------------------------------------------------------ 1498

NR_025622.1 ------------------------------------------------------------ 1496

NR_025621.1 ------------------------------------------------------------ 1497

EU741199.1 ------------------------------------------------------------ 1499

NR_112400.1 ------------------------------------------------------------ 1480

11993_B; aggcagcagtggggaatattgcacaatgggcgaaagcctgatgcagcgacgccgcgtgag 394

11993_A; aggcagcagtggggaatattgcacaatgggcgaaagcctgatgcagcgacgccgcgtgag 1824

KY318506.1 ------------------------------------------------------------ 1520

MG198705.1 ------------------------------------------------------------ 1453

KY523106.1 ------------------------------------------------------------ 1414

MH430523.2 ------------------------------------------------------------ 1534

DQ026648.1 ------------------------------------------------------------ 1513

MT669274.1 ------------------------------------------------------------ 1396

MF077006.1 ------------------------------------------------------------ 920

EU603342.1 ------------------------------------------------------------ 1425

JF827350.1 ------------------------------------------------------------ 1487

NR_112352.1 ------------------------------------------------------------ 944

NR_024760.1 ------------------------------------------------------------ 1489

AJ007399.1 ------------------------------------------------------------ 1483

AB184480.1 ------------------------------------------------------------ 1479

MH021965.1 ------------------------------------------------------------ 1473

KT781120.1 ------------------------------------------------------------ 1498

NR_025622.1 ------------------------------------------------------------ 1496

NR_025621.1 ------------------------------------------------------------ 1497

EU741199.1 ------------------------------------------------------------ 1499

NR_112400.1 ------------------------------------------------------------ 1480

11993_B; ggatgacggccttcgggttgtaaacctctttcagtagggaagaagcgcaagtgacggtac 454

11993_A; ggatgacggccttcgggttgtaaacctctttcagtagggaagaagcgcaagtgacggtac 1884

KY318506.1 ------------------------------------------------------------ 1520

MG198705.1 ------------------------------------------------------------ 1453

KY523106.1 ------------------------------------------------------------ 1414

MH430523.2 ------------------------------------------------------------ 1534

DQ026648.1 ------------------------------------------------------------ 1513

MT669274.1 ------------------------------------------------------------ 1396

MF077006.1 ------------------------------------------------------------ 920

EU603342.1 ------------------------------------------------------------ 1425

JF827350.1 ------------------------------------------------------------ 1487

NR_112352.1 ------------------------------------------------------------ 944

NR_024760.1 ------------------------------------------------------------ 1489

AJ007399.1 ------------------------------------------------------------ 1483

AB184480.1 ------------------------------------------------------------ 1479

MH021965.1 ------------------------------------------------------------ 1473

KT781120.1 ------------------------------------------------------------ 1498

NR_025622.1 ------------------------------------------------------------ 1496

NR_025621.1 ------------------------------------------------------------ 1497

EU741199.1 ------------------------------------------------------------ 1499

NR_112400.1 ------------------------------------------------------------ 1480

11993_B; ctacagaagaagcaccggctaactacgtgccagcagccgcggtaatacgtagggtgcgag 514

11993_A; ctacagaagaagcaccggctaactacgtgccagcagccgcggtaatacgtagggtgcgag 1944

KY318506.1 ------------------------------------------------------------ 1520

MG198705.1 ------------------------------------------------------------ 1453

KY523106.1 ------------------------------------------------------------ 1414

MH430523.2 ------------------------------------------------------------ 1534

DQ026648.1 ------------------------------------------------------------ 1513

MT669274.1 ------------------------------------------------------------ 1396

MF077006.1 ------------------------------------------------------------ 920

EU603342.1 ------------------------------------------------------------ 1425

JF827350.1 ------------------------------------------------------------ 1487

NR_112352.1 ------------------------------------------------------------ 944

NR_024760.1 ------------------------------------------------------------ 1489

AJ007399.1 ------------------------------------------------------------ 1483

AB184480.1 ------------------------------------------------------------ 1479

MH021965.1 ------------------------------------------------------------ 1473

KT781120.1 ------------------------------------------------------------ 1498

NR_025622.1 ------------------------------------------------------------ 1496

NR_025621.1 ------------------------------------------------------------ 1497

EU741199.1 ------------------------------------------------------------ 1499

NR_112400.1 ------------------------------------------------------------ 1480

11993_B; cgttgtccggaattattgggcgtaaagagctcgtaggcggcttgtcacgtcggatgtgaa 574

11993_A; cgttgtccggaattattgggcgtaaagagctcgtaggcggcttgtcacgtcggatgtgaa 2004

KY318506.1 ------------------------------------------------------------ 1520

MG198705.1 ------------------------------------------------------------ 1453

KY523106.1 ------------------------------------------------------------ 1414

MH430523.2 ------------------------------------------------------------ 1534

DQ026648.1 ------------------------------------------------------------ 1513

MT669274.1 ------------------------------------------------------------ 1396

MF077006.1 ------------------------------------------------------------ 920

EU603342.1 ------------------------------------------------------------ 1425

JF827350.1 ------------------------------------------------------------ 1487

NR_112352.1 ------------------------------------------------------------ 944

NR_024760.1 ------------------------------------------------------------ 1489

AJ007399.1 ------------------------------------------------------------ 1483

AB184480.1 ------------------------------------------------------------ 1479

MH021965.1 ------------------------------------------------------------ 1473

KT781120.1 ------------------------------------------------------------ 1498

NR_025622.1 ------------------------------------------------------------ 1496

NR_025621.1 ------------------------------------------------------------ 1497

EU741199.1 ------------------------------------------------------------ 1499

NR_112400.1 ------------------------------------------------------------ 1480

11993_B; agcccggggcttaaccccgggtctgcattcgatacgggcaggctagagttcggtagggga 634

11993_A; agcccggggcttaaccccgggtctgcattcgatacgggcaggctagagttcggtagggga 2064

KY318506.1 ------------------------------------------------------------ 1520

MG198705.1 ------------------------------------------------------------ 1453

KY523106.1 ------------------------------------------------------------ 1414

MH430523.2 ------------------------------------------------------------ 1534

DQ026648.1 ------------------------------------------------------------ 1513

MT669274.1 ------------------------------------------------------------ 1396

MF077006.1 ------------------------------------------------------------ 920

EU603342.1 ------------------------------------------------------------ 1425

JF827350.1 ------------------------------------------------------------ 1487

NR_112352.1 ------------------------------------------------------------ 944

NR_024760.1 ------------------------------------------------------------ 1489

AJ007399.1 ------------------------------------------------------------ 1483

AB184480.1 ------------------------------------------------------------ 1479

MH021965.1 ------------------------------------------------------------ 1473

KT781120.1 ------------------------------------------------------------ 1498

NR_025622.1 ------------------------------------------------------------ 1496

NR_025621.1 ------------------------------------------------------------ 1497

EU741199.1 ------------------------------------------------------------ 1499

NR_112400.1 ------------------------------------------------------------ 1480

11993_B; gatcggaattcctggtgtagcggtgaaatgcgcagatatcaggaggaacaccggtggcga 694

11993_A; gatcggaattcctggtgtagcggtgaaatgcgcagatatcaggaggaacaccggtggcga 2124

KY318506.1 ------------------------------------------------------------ 1520

MG198705.1 ------------------------------------------------------------ 1453

KY523106.1 ------------------------------------------------------------ 1414

MH430523.2 ------------------------------------------------------------ 1534

DQ026648.1 ------------------------------------------------------------ 1513

MT669274.1 ------------------------------------------------------------ 1396

MF077006.1 ------------------------------------------------------------ 920

EU603342.1 ------------------------------------------------------------ 1425

JF827350.1 ------------------------------------------------------------ 1487

NR_112352.1 ------------------------------------------------------------ 944

NR_024760.1 ------------------------------------------------------------ 1489

AJ007399.1 ------------------------------------------------------------ 1483

AB184480.1 ------------------------------------------------------------ 1479

MH021965.1 ------------------------------------------------------------ 1473

KT781120.1 ------------------------------------------------------------ 1498

NR_025622.1 ------------------------------------------------------------ 1496

NR_025621.1 ------------------------------------------------------------ 1497

EU741199.1 ------------------------------------------------------------ 1499

NR_112400.1 ------------------------------------------------------------ 1480

11993_B; aggcggatctctgggccgatactgacgctgaggagcgaaagcgtggggagcgaacaggat 754

11993_A; aggcggatctctgggccgatactgacgctgaggagcgaaagcgtggggagcgaacaggat 2184

KY318506.1 ------------------------------------------------------------ 1520

MG198705.1 ------------------------------------------------------------ 1453

KY523106.1 ------------------------------------------------------------ 1414

MH430523.2 ------------------------------------------------------------ 1534

DQ026648.1 ------------------------------------------------------------ 1513

MT669274.1 ------------------------------------------------------------ 1396

MF077006.1 ------------------------------------------------------------ 920

EU603342.1 ------------------------------------------------------------ 1425

JF827350.1 ------------------------------------------------------------ 1487

NR_112352.1 ------------------------------------------------------------ 944

NR_024760.1 ------------------------------------------------------------ 1489

AJ007399.1 ------------------------------------------------------------ 1483

AB184480.1 ------------------------------------------------------------ 1479

MH021965.1 ------------------------------------------------------------ 1473

KT781120.1 ------------------------------------------------------------ 1498

NR_025622.1 ------------------------------------------------------------ 1496

NR_025621.1 ------------------------------------------------------------ 1497

EU741199.1 ------------------------------------------------------------ 1499

NR_112400.1 ------------------------------------------------------------ 1480

11993_B; tagataccctggtagtccacgccgtaaacgttgggaactaggtgtgggcgacattccacg 814

11993_A; tagataccctggtagtccacgccgtaaacgttgggaactaggtgtgggcgacattccacg 2244

KY318506.1 ------------------------------------------------------------ 1520

MG198705.1 ------------------------------------------------------------ 1453

KY523106.1 ------------------------------------------------------------ 1414

MH430523.2 ------------------------------------------------------------ 1534

DQ026648.1 ------------------------------------------------------------ 1513

MT669274.1 ------------------------------------------------------------ 1396

MF077006.1 ------------------------------------------------------------ 920

EU603342.1 ------------------------------------------------------------ 1425

JF827350.1 ------------------------------------------------------------ 1487

NR_112352.1 ------------------------------------------------------------ 944

NR_024760.1 ------------------------------------------------------------ 1489

AJ007399.1 ------------------------------------------------------------ 1483

AB184480.1 ------------------------------------------------------------ 1479

MH021965.1 ------------------------------------------------------------ 1473

KT781120.1 ------------------------------------------------------------ 1498

NR_025622.1 ------------------------------------------------------------ 1496

NR_025621.1 ------------------------------------------------------------ 1497

EU741199.1 ------------------------------------------------------------ 1499

NR_112400.1 ------------------------------------------------------------ 1480

11993_B; ttgtccgtgccgcagctaacgcattaagttccccgcctggggagtacacggccgcaaggc 874

11993_A; ttgtccgtgccgcagctaacgcattaagttccccgcctggggagtacacggccgcaaggc 2304

KY318506.1 ------------------------------------------------------------ 1520

MG198705.1 ------------------------------------------------------------ 1453

KY523106.1 ------------------------------------------------------------ 1414

MH430523.2 ------------------------------------------------------------ 1534

DQ026648.1 ------------------------------------------------------------ 1513

MT669274.1 ------------------------------------------------------------ 1396

MF077006.1 ------------------------------------------------------------ 920

EU603342.1 ------------------------------------------------------------ 1425

JF827350.1 ------------------------------------------------------------ 1487

NR_112352.1 ------------------------------------------------------------ 944

NR_024760.1 ------------------------------------------------------------ 1489

AJ007399.1 ------------------------------------------------------------ 1483

AB184480.1 ------------------------------------------------------------ 1479

MH021965.1 ------------------------------------------------------------ 1473

KT781120.1 ------------------------------------------------------------ 1498

NR_025622.1 ------------------------------------------------------------ 1496

NR_025621.1 ------------------------------------------------------------ 1497

EU741199.1 ------------------------------------------------------------ 1499

NR_112400.1 ------------------------------------------------------------ 1480

11993_B; taaaactcaaaggaattgacgggggcccgcacaagcggcggagcatgtggcttaattcga 934

11993_A; taaaactcaaaggaattgacgggggcccgcacaagcggcggagcatgtggcttaattcga 2364

KY318506.1 ------------------------------------------------------------ 1520

MG198705.1 ------------------------------------------------------------ 1453

KY523106.1 ------------------------------------------------------------ 1414

MH430523.2 ------------------------------------------------------------ 1534

DQ026648.1 ------------------------------------------------------------ 1513

MT669274.1 ------------------------------------------------------------ 1396

MF077006.1 ------------------------------------------------------------ 920

EU603342.1 ------------------------------------------------------------ 1425

JF827350.1 ------------------------------------------------------------ 1487

NR_112352.1 ------------------------------------------------------------ 944

NR_024760.1 ------------------------------------------------------------ 1489

AJ007399.1 ------------------------------------------------------------ 1483

AB184480.1 ------------------------------------------------------------ 1479

MH021965.1 ------------------------------------------------------------ 1473

KT781120.1 ------------------------------------------------------------ 1498

NR_025622.1 ------------------------------------------------------------ 1496

NR_025621.1 ------------------------------------------------------------ 1497

EU741199.1 ------------------------------------------------------------ 1499

NR_112400.1 ------------------------------------------------------------ 1480

11993_B; cgcaacgcgaagaaccttaccaaggcttgacatacaccggaaaaccgtggagacacggtc 994

11993_A; cgcaacgcgaagaaccttaccaaggcttgacatacaccggaaaaccgtggagacacggtc 2424

KY318506.1 ------------------------------------------------------------ 1520

MG198705.1 ------------------------------------------------------------ 1453

KY523106.1 ------------------------------------------------------------ 1414

MH430523.2 ------------------------------------------------------------ 1534

DQ026648.1 ------------------------------------------------------------ 1513

MT669274.1 ------------------------------------------------------------ 1396

MF077006.1 ------------------------------------------------------------ 920

EU603342.1 ------------------------------------------------------------ 1425

JF827350.1 ------------------------------------------------------------ 1487

NR_112352.1 ------------------------------------------------------------ 944

NR_024760.1 ------------------------------------------------------------ 1489

AJ007399.1 ------------------------------------------------------------ 1483

AB184480.1 ------------------------------------------------------------ 1479

MH021965.1 ------------------------------------------------------------ 1473

KT781120.1 ------------------------------------------------------------ 1498

NR_025622.1 ------------------------------------------------------------ 1496

NR_025621.1 ------------------------------------------------------------ 1497

EU741199.1 ------------------------------------------------------------ 1499

NR_112400.1 ------------------------------------------------------------ 1480

11993_B; ccccttgtggtcggtgtacaggtggtgcatggctgtcgtcagctcgtgatcgtgagatgt 1054

11993_A; ccccttgtggtcggtgtacaggtggtgcatggctgtcgtcagctcgtgatcgtgagatgt 2484

KY318506.1 ------------------------------------------------------------ 1520

MG198705.1 ------------------------------------------------------------ 1453

KY523106.1 ------------------------------------------------------------ 1414

MH430523.2 ------------------------------------------------------------ 1534

DQ026648.1 ------------------------------------------------------------ 1513

MT669274.1 ------------------------------------------------------------ 1396

MF077006.1 ------------------------------------------------------------ 920

EU603342.1 ------------------------------------------------------------ 1425

JF827350.1 ------------------------------------------------------------ 1487

NR_112352.1 ------------------------------------------------------------ 944

NR_024760.1 ------------------------------------------------------------ 1489

AJ007399.1 ------------------------------------------------------------ 1483

AB184480.1 ------------------------------------------------------------ 1479

MH021965.1 ------------------------------------------------------------ 1473

KT781120.1 ------------------------------------------------------------ 1498

NR_025622.1 ------------------------------------------------------------ 1496

NR_025621.1 ------------------------------------------------------------ 1497

EU741199.1 ------------------------------------------------------------ 1499

NR_112400.1 ------------------------------------------------------------ 1480

11993_B; tgggttaagtcccgcaacgagcgcaacccttgttctgtgttgccagcatgcctttcgggg 1114

11993_A; tgggttaagtcccgcaacgagcgcaacccttgttctgtgttgccagcatgcctttcgggg 2544

KY318506.1 ------------------------------------------------------------ 1520

MG198705.1 ------------------------------------------------------------ 1453

KY523106.1 ------------------------------------------------------------ 1414

MH430523.2 ------------------------------------------------------------ 1534

DQ026648.1 ------------------------------------------------------------ 1513

MT669274.1 ------------------------------------------------------------ 1396

MF077006.1 ------------------------------------------------------------ 920

EU603342.1 ------------------------------------------------------------ 1425

JF827350.1 ------------------------------------------------------------ 1487

NR_112352.1 ------------------------------------------------------------ 944

NR_024760.1 ------------------------------------------------------------ 1489

AJ007399.1 ------------------------------------------------------------ 1483

AB184480.1 ------------------------------------------------------------ 1479

MH021965.1 ------------------------------------------------------------ 1473

KT781120.1 ------------------------------------------------------------ 1498

NR_025622.1 ------------------------------------------------------------ 1496

NR_025621.1 ------------------------------------------------------------ 1497

EU741199.1 ------------------------------------------------------------ 1499

NR_112400.1 ------------------------------------------------------------ 1480

11993_B; tgatggggactcacaggagactgccggggtcaactcggaggaaggcatggggacgacgtc 1174

11993_A; tgatggggactcacaggagactgccggggtcaactcggaggaaggcatggggacgacgtc 2604

KY318506.1 ------------------------------------------------------------ 1520

MG198705.1 ------------------------------------------------------------ 1453

KY523106.1 ------------------------------------------------------------ 1414

MH430523.2 ------------------------------------------------------------ 1534

DQ026648.1 ------------------------------------------------------------ 1513

MT669274.1 ------------------------------------------------------------ 1396

MF077006.1 ------------------------------------------------------------ 920

EU603342.1 ------------------------------------------------------------ 1425

JF827350.1 ------------------------------------------------------------ 1487

NR_112352.1 ------------------------------------------------------------ 944

NR_024760.1 ------------------------------------------------------------ 1489

AJ007399.1 ------------------------------------------------------------ 1483

AB184480.1 ------------------------------------------------------------ 1479

MH021965.1 ------------------------------------------------------------ 1473

KT781120.1 ------------------------------------------------------------ 1498

NR_025622.1 ------------------------------------------------------------ 1496

NR_025621.1 ------------------------------------------------------------ 1497

EU741199.1 ------------------------------------------------------------ 1499

NR_112400.1 ------------------------------------------------------------ 1480

11993_B; aagtcatcatgccccttatgtcttgggctgcacacgtgctacaatgacagccggtacaat 1234

11993_A; aagtcatcatgccccttatgtcttgggctgcacacgtgctacaatgacagccggtacaat 2664

KY318506.1 ------------------------------------------------------------ 1520

MG198705.1 ------------------------------------------------------------ 1453

KY523106.1 ------------------------------------------------------------ 1414

MH430523.2 ------------------------------------------------------------ 1534

DQ026648.1 ------------------------------------------------------------ 1513

MT669274.1 ------------------------------------------------------------ 1396

MF077006.1 ------------------------------------------------------------ 920

EU603342.1 ------------------------------------------------------------ 1425

JF827350.1 ------------------------------------------------------------ 1487

NR_112352.1 ------------------------------------------------------------ 944

NR_024760.1 ------------------------------------------------------------ 1489

AJ007399.1 ------------------------------------------------------------ 1483

AB184480.1 ------------------------------------------------------------ 1479

MH021965.1 ------------------------------------------------------------ 1473

KT781120.1 ------------------------------------------------------------ 1498

NR_025622.1 ------------------------------------------------------------ 1496

NR_025621.1 ------------------------------------------------------------ 1497

EU741199.1 ------------------------------------------------------------ 1499

NR_112400.1 ------------------------------------------------------------ 1480

11993_B; gagctgcgataccgcgaggtggagcgaatctcaaaaagccggtctcagttcggattgggg 1294

11993_A; gagctgcgataccgcgaggtggagcgaatctcaaaaagccggtctcagttcggattgggg 2724

KY318506.1 ------------------------------------------------------------ 1520

MG198705.1 ------------------------------------------------------------ 1453

KY523106.1 ------------------------------------------------------------ 1414

MH430523.2 ------------------------------------------------------------ 1534

DQ026648.1 ------------------------------------------------------------ 1513

MT669274.1 ------------------------------------------------------------ 1396

MF077006.1 ------------------------------------------------------------ 920

EU603342.1 ------------------------------------------------------------ 1425

JF827350.1 ------------------------------------------------------------ 1487

NR_112352.1 ------------------------------------------------------------ 944

NR_024760.1 ------------------------------------------------------------ 1489

AJ007399.1 ------------------------------------------------------------ 1483

AB184480.1 ------------------------------------------------------------ 1479

MH021965.1 ------------------------------------------------------------ 1473

KT781120.1 ------------------------------------------------------------ 1498

NR_025622.1 ------------------------------------------------------------ 1496

NR_025621.1 ------------------------------------------------------------ 1497

EU741199.1 ------------------------------------------------------------ 1499

NR_112400.1 ------------------------------------------------------------ 1480

11993_B; tctgcaactcgaccccatgaagtcggagtcgctagtaatcgcagatcagcattgctgcgg 1354

11993_A; tctgcaactcgaccccatgaagtcggagtcgctagtaatcgcagatcagcattgctgcgg 2784

KY318506.1 ------------------------------------------------------------ 1520

MG198705.1 ------------------------------------------------------------ 1453

KY523106.1 ------------------------------------------------------------ 1414

MH430523.2 ------------------------------------------------------------ 1534

DQ026648.1 ------------------------------------------------------------ 1513

MT669274.1 ------------------------------------------------------------ 1396

MF077006.1 ------------------------------------------------------------ 920

EU603342.1 ------------------------------------------------------------ 1425

JF827350.1 ------------------------------------------------------------ 1487

NR_112352.1 ------------------------------------------------------------ 944

NR_024760.1 ------------------------------------------------------------ 1489

AJ007399.1 ------------------------------------------------------------ 1483

AB184480.1 ------------------------------------------------------------ 1479

MH021965.1 ------------------------------------------------------------ 1473

KT781120.1 ------------------------------------------------------------ 1498

NR_025622.1 ------------------------------------------------------------ 1496

NR_025621.1 ------------------------------------------------------------ 1497

EU741199.1 ------------------------------------------------------------ 1499

NR_112400.1 ------------------------------------------------------------ 1480

11993_B; tgaatacgttcccgggccttgtacacaccgcccgtcacgtaacgatcg 1402

11993_A; tgaatacgttcccgggccttgtacacaccgcccgtcacgtaacgatcg 2832

KY318506.1 ------------------------------------------------ 1520

MG198705.1 ------------------------------------------------ 1453

KY523106.1 ------------------------------------------------ 1414

MH430523.2 ------------------------------------------------ 1534

DQ026648.1 ------------------------------------------------ 1513

MT669274.1 ------------------------------------------------ 1396

MF077006.1 ------------------------------------------------ 920

EU603342.1 ------------------------------------------------ 1425

JF827350.1 ------------------------------------------------ 1487

NR_112352.1 ------------------------------------------------ 944

NR_024760.1 ------------------------------------------------ 1489

AJ007399.1 ------------------------------------------------ 1483

AB184480.1 ------------------------------------------------ 1479

MH021965.1 ------------------------------------------------ 1473

KT781120.1 ------------------------------------------------ 1498

NR_025622.1 ------------------------------------------------ 1496

NR_025621.1 ------------------------------------------------ 1497

EU741199.1 ------------------------------------------------ 1499

NR_112400.1 ------------------------------------------------ 1480

**
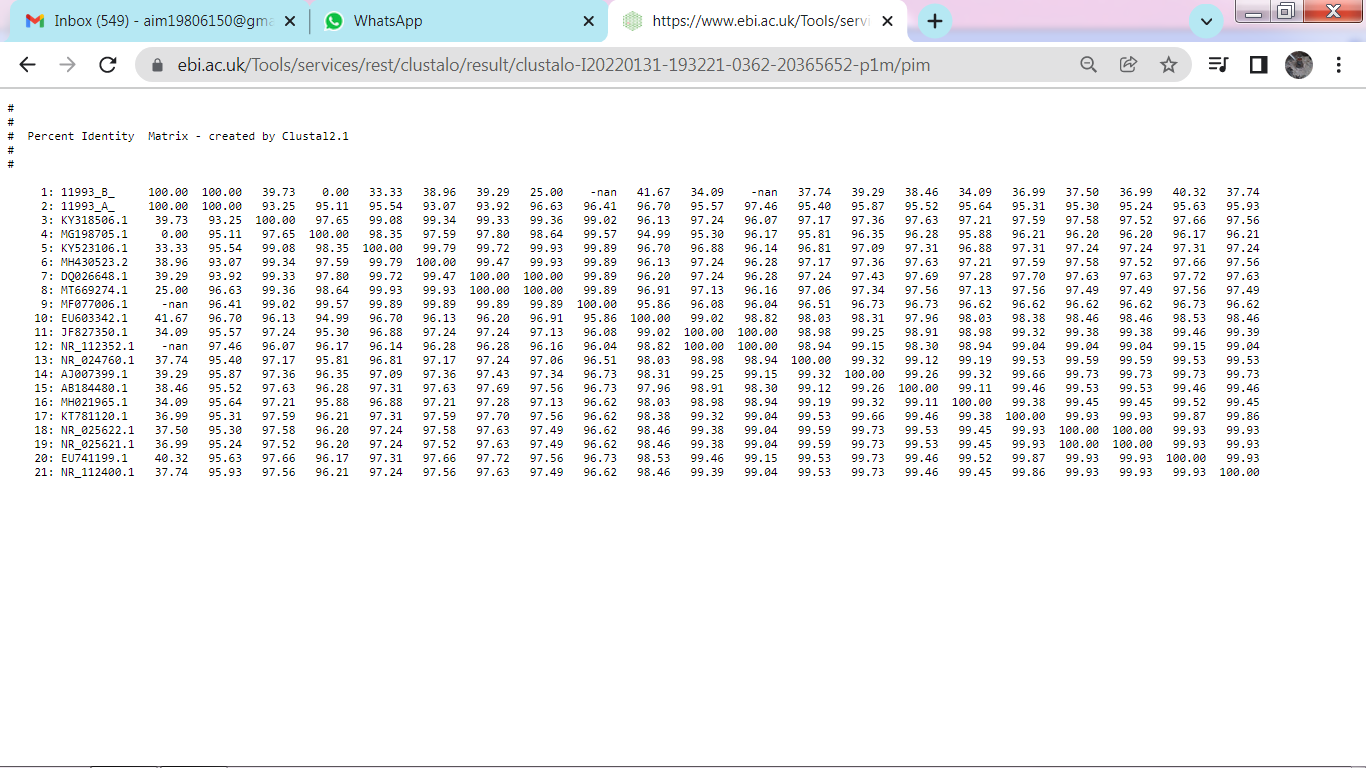
**

**Supplementary Table S1: Identification of acetaminophen/paracetamol bio-degradation products extracts 1a, 1b, 2a and 2b MTT assay cytotoxicity against acetaminophen/paracetamol using 2 HepG2 and MCF7 cancer cell lines**

| **Cell line** | **HepG2** | | | | **+ve**  **control**  **APAP** | **-ve**  **control**  **DMSO** | **MCF7** | | | | **+ve**  **control**  **APAP** | **-ve**  **control**  **DMSO** |
| --- | --- | --- | --- | --- | --- | --- | --- | --- | --- | --- | --- | --- |
| **Conc.** | **Extract samples** | | | |  |  | **Extract samples** | | | |  |  |
| **µg/ml** | **1a SH** | **1b ST** | **2a SH** | **2b ST** |  |  | **1a SH** | **1b ST** | **2a SH** | **2b ST** |  |  |
| 5000 | 28.17 | 28.83 | 28.89 | 30.19 | 23.94 | 24.09 | 25.67 | 25.50 | 28.25 | 26.67 | 26.85 | 26.11 |
| 2500 | 28.83 | 31.17 | 30.93 | 32.59 | 24.09 | 26.36 | 28.17 | 27.00 | 32.28 | 28.42 | 28.33 | 27.04 |
| 1250 | 30.50 | 33.50 | 32.78 | 38.70 | 24.24 | 34.39 | 32.17 | 28.67 | 40.70 | 29.47 | 28.33 | 28.15 |
| 625 | 31.17 | 35.17 | 35.93 | 38.89 | 26.36 | 46.21 | 40.17 | 36.67 | 41.75 | 32.28 | 29.81 | 31.11 |
| 312.50 | 31.67 | 39.83 | 38.52 | 38.89 | 27.12 | 50.30 | 59.00 | 56.83 | 42.28 | 49.82 | 32.22 | 36.85 |
| 156.25 | 66.67 | 57.00 | 43.70 | 47.78 | 48.33 | 55.00 | 89.00 | 93.00 | 73.33 | 81.75 | 42.78 | 65.19 |
| 78.13 | 86.33 | 83.50 | 62.96 | 75.00 | 59.39 | 81.97 | 90.33 | 93.67 | 83.51 | 96.14 | 70.19 | 82.96 |
| 39.06 | 105.00 | 108.00 | 79.44 | 84.44 | 61.97 | 90.45 | 93.67 | 99.17 | 88.77 | 103.68 | 71.85 | 83.33 |
| 19.53 | 108.17 | 109.50 | 91.48 | 95.93 | 88.48 | 107.12 | 96.50 | 99.67 | 100.70 | 111.23 | 72.04 | 91.30 |
| 9.77 | 108.50 | 110.17 | 84.44 | 102.41 | 91.52 | 128.03 | 101.67 | 101.00 | 103.86 | 111.58 | 76.30 | 95.00 |
| 4.88 | 111.67 | 111.50 | 89.63 | 112.04 | 104.24 | 138.48 | 102.67 | 103.33 | 105.79 | 112.98 | 77.78 | 106.85 |
| 2.44 | 113.00 | 112.33 | 100.93 | 113.89 | 106.82 | 104.85 | 107.00 | 103.83 | 105.79 | 117.54 | 98.15 | 113.70 |
| **IC50 µg/ml** | **192.28** | **200.60** | **126.64** | **119.93** | **108.60** | **-** | **441.76** | **370.06** | **285.04** | **305.00** | **108.46** | **-** |

[Extract 1a; M33 strain extract shaking, Extract 1b; M33 strain extract static, Extract 2a; RS2 strain extract shaking, Extract 2b; Rs2 strain extract static, paracetamol is the positive control, % cell viability = (mean absorbance of treated sample/mean absorbance of negative control sample) × 100, % death rate = 100 – (% cell viability), and the inhibitory concentration of 50% (IC50) was measured from the exponential curve of viability against concentration (dose–response curve); using Master –plex-2010 program, DMSO is the negative control, paracetamol/APAP is the positive control.]

**HepG2 and MCF7 IHC photo-micrographs imaged by an Inverted Microscope (Supplementary Figures S2)**

Morphological changes visualization in cancer cell lines by Inverted Microscopy (Phase Contrast) to confirm apoptotic cells morphological alterations of shrinkage, nuclear condensation, and fragmentation, and apoptotic bodies formation as well as loss of attachment to neighboring cells, all confirming apoptosis.

<https://drive.google.com/folderview?id=1XYW6tBq3g9Vw78Q7hQlIuK0ZOHDraPBm>

Para: paracetamol/acetaminophen,

W1: extract 1 shaking (1a),

W2: extract 1 static (1b),

W3: extract 2 shaking (2a),

and W4: extract 2 static (2b).

**Supplementary Table S2: Effect of acetaminophen acute single oral dose (200 mg/k.g BW) and the acetaminophen bio-degradation products IC50 on blood liver function tests, liver tissue oxidative stress markers (SOD and MDA), liver tissue antioxidant levels (TAC and CAT) as well as liver IL-6 and caspase-9, as an *in vivo* acute single oral toxicity test**

| **Biochemical parameters** | **Serum LFTs** | | | **Tissue oxidative stress** | |  | **Liver tissue homogenate** | | | | | |  |
| --- | --- | --- | --- | --- | --- | --- | --- | --- | --- | --- | --- | --- | --- |
|  | **ALT** | **AST** | **GGT** | **SOD** | **MDA** |  | **TAC** | | **CAT** | | **IL-6** | **Caspase-9** | |
| **Groups/Unit** | **u/L** | | | **u/gm tissue** | **nmol/gm tissue** |  |  |  | | **pg/ml** | | | **ng/ml** |
| **1** | 29.5^b^ ± 2.3 | 20^c^ ± 1.5 | 10.5^c^ ± 0.5 | 433.6^b^ ± 20.2 | 54.7^c^ ± 1.8 |  | 11^a^ ± 0.6 | 1236^b^ ± 87 | | 174^c^ ± 10.4 | | | 0.9^d^ ± 0.06 |
| **2** | 28^b^ ± 2.14 | 20.6^b,c^ ± 1.3 | 6.5^d^ ± 0.3 | 522.2^a^ ± 22.4 | 74.4^b^ ± 2.5 |  | 2.8^b^ ± 0.14 | 1407^a,b^ ± 87 | | 254^b^ ± 15 | | | 1.6^c^ ± 0.07 |
| **3** | 70^a^ ± 2.1 | 41^a^ ± 1.7 | 16.5^a^ ± 0.8 | 383.2^b^ ± 14.7 | 76.3^b^ ± 4 |  | 2.3^b^ ± 0.06 | 1194^b^ ± 52 | | 408.6^a^ ± 16.6 | | | 4.72^a^ ± 0.4 |
| **4** | 20^c^ ± 0.7 | 19^c^ ± 1.3 | 12.5^b^ ± 0.4 | 394^b^ ± 27.7 | 151^a^ ± 5.4 |  | 2.9^b^ ± 0.2 | 1515^a^ ± 77 | | 257^b^ ± 14.7 | | | 2.3^b^ ± 0.14 |
| **5** | 19.5^c^ ± 1.2 | 25^b^ ± 1.6 | 7^d^ ± 0.5 | 522.6^a^ ± 44.8 | 79.3^b^ ± 4.3 |  | 2.6^b^ ± 0.14 | 1262^b^ ± 74 | | 429.4^a^ ± 15 | | | 4.27^a^ ± 0.2 |

Data are as mean ± SEM in sera or per gram tissue protein in the tissue homogenate to total protein conc. Calculated as ng/ml, experiments were performed in triplicate and repeated twice, significance criterion set to 0.05 level of probability *p*, a>b>c>d significant mean difference by ANOVA followed by Duncan post hoc test. [Group 1; normal control, group 2; DMSO negative control, group 3; acetaminophen/paracetamol positive control, group 4; Extract 1b (M33 bio-degradation products in static condition); group5; extract 1a (M33 bio-degradation products in shaking condition).
